# The urothelial cell line UROtsa transformed by arsenite and cadmium display basal characteristics associated with muscle invasive urothelial cancers

**DOI:** 10.1371/journal.pone.0207877

**Published:** 2018-12-14

**Authors:** Zachary E. Hoggarth, Danyelle B. Osowski, Brooke A. Freeberg, Scott H. Garrett, Donald A. Sens, Mary Ann Sens, Xu Dong Zhou, Ke K. Zhang, Seema Somji

**Affiliations:** Department of Pathology, School of Medicine and Health Sciences, University of North Dakota, Grand Forks, North Dakota, United States of America; Thomas Jefferson University, UNITED STATES

## Abstract

Muscle invasive urothelial carcinomas are divided into various molecular subtypes with basal and luminal subtypes being the prominent ones. The basal muscle-invasive urothelial carcinomas are generally more aggressive at presentation and significantly enriched with squamous features. Our laboratory has developed an *in-vitro* model of urothelial cancer by transforming the immortalized cell line UROtsa with arsenite (As^3+^) and cadmium (Cd^2+^). In this study, we characterized the tumors formed by these transformed cell lines as more basal-like based on their gene expression patterns with increased expression of KRT1, KRT5, KRT6, KRT14, KRT16, KRT17 and CD44. In addition, histological examination of these tumor transplants showed squamous features enriched in basal muscle invasive urothelial carcinomas. The expression of these genes increased in the transformed cell lines as well as in the urospheres, which are putative cancer initiating cells/stem cells derived from the cell lines. There was also increased expression of these genes in the urospheres derived from the parent UROtsa cell line. Thus, our data shows that the As^3+^ and Cd^2+^-transformed cell lines and their derived tumor transplants have gene expression profiles similar to the basal subtype of muscle invasive bladder carcinomas with tumors having enriched squamous features. The increased expression of basal markers in the urospheres suggests that stem cells may be involved in the development of squamous differentiation seen in some of the muscle invasive bladder carcinomas.

## Introduction

Gene expression profiling has been successfully employed in patients with breast cancer to identify molecular subtypes (basal/triple negative, HER2^+^, luminal A, and luminal B) that behave clinically as distinct disease entities [1]. Within this profile, luminal breast cancers respond to estrogen receptor targeted therapy, HER2^+^ tumors to Herceptin and other ErbB2-blocking agents, and basal tumors to chemotherapy only. This success in subtyping breast cancers has led to similar efforts in a variety of other organ-specific cancers. For urothelial carcinoma (UC), three investigative teams (Lund University, MD Anderson Cancer Center and University of North Carolina) have advanced the existence of intrinsic subtypes of UC [2–4]. The investigative group associated with Lund University initially defined three UC molecular subtypes: urobasal (Uro), genomically unstable (GU), and squamous-cell-carcinoma-like (SCCL) [2]. The study showed that the three subtypes did not correspond directly to pathological stage and grade, and differed in fundamental biological aspects and survival patterns [2, 5]. A subsequent study employing 167 primary stage T1 tumors demonstrated that rapidly progressing T1 tumors were of subtype GU or SCCL, possessing either a high progression risk score or an elevated CD3^+^ cell count [6]. Ultimately, the subtypes defined by the Lund investigators were expanded to six categories due to the complexity of muscle-invasive urothelial carcinoma (MIUC) [7–9]. The investigative group associated with MD Anderson Cancer Center focused only on samples from MIUC [3]. The study identified that the muscle invasive tumors segregated into distinct basal and luminal subtypes. The basal MIUCs were more aggressive at presentation and significantly enriched with squamous features. Investigators at the University of North Carolina found similar results on muscle invasive tumors with segregation into basal and luminal subtypes [4]. A general finding in the above studies was the association of more basal subtypes with aggressive disease, including the enrichment of genes and features associated with squamous differentiation.

This laboratory developed and advanced the UROtsa cell line as a model system for the study of environmentally induced human urothelial cancer [10–14]. The laboratory demonstrated that the UROtsa cells, an immortalized but non-tumorigenic model of human urothelium, could be malignantly transformed by both cadmium (Cd^2+^) and arsenite (As^3+^) as evidenced by tumor formation in immune compromised mice [11]. In total, the laboratory produced seven independently generated Cd^2+^-transformed cell lines and six independently generated As^3+^- transformed cell lines, all of which form subcutaneous tumors in immune compromised mice [12–15]. The histology of subcutaneous tumor transplants produced by all the above Cd^2+^-and As^3+^-transformed UROtsa cells displayed histologic features of human urothelial carcinoma, each with variable focal areas of prominent squamous differentiation [11–13]. The presence of focal areas of squamous differentiation were of initial concern for the general applicability of this model system, since UC’s with clear histologic evidence of squamous differentiation are generally viewed as being quite rare for UC’s in the western world. However, the above referenced molecular sub-tying of UC’s clearly shows that squamous features are associated with the basal subtype classification of UC’s, possibly indicating a higher prevalence than recognized by histologic examination. In the molecular subtyping of muscle-invasive UC’s, basal UC’s with squamous features are associated with an aggressive phenotype [3]. These findings provide initial evidence that the UROtsa model of UC might represent an aggressive basal phenotype with prominent squamous differentiation. The present study has several goals. The first is to determine if tumors generated from the UROtsa cell culture model reflect the basal subtype of UC associated with muscle-invasive and more aggressive UCs. The second is to determine if the As^3+^ and Cd^2+^-transformed UROtsa cell lines have a gene signature associated with the basal subtype of MIBCs, and if this signature is enhanced in cancer-initiating-cells (CICs) isolated from these cell lines. The last goal is to determine if the parental cell line contains a population of progenitor/stem like cells enriched in some of the basal markers found in MIBCs.

## Materials and methods

### Animals

Athymic nude (NCR-*nu/nu*) mice were used in these studies. The mice were housed four to a cage at 22°C under a 12-hour light/dark cycle. Food and water was available *ad libitum*. Mouse heterotransplants of the UROtsa transformed cell cultures were produced by subcutaneous injection at a dose of 1 X 10^6^ cells in the dorsal thoracic midline of athymic nude (NCR-*nu/nu*) mice as described previously [11]. This study adhered to all recommendation dictated in the Guide for the Care and Use of Laboratory Animals of the NIH. The size of the tumors were assessed weekly using a ruler and the animals were sacrificed when the size of the tumor was approximately 1.5–1.8 cm or when dictated by clinical conditions. The animals were euthanized by CO_2_ asphyxiation and conformed to American Veterinary Medical Association Guideline on Euthanasia. Death was confirmed by ascertaining cardiac and respiratory arrest following which the organs and tumor were harvested. Care of taken to ensure that there was no distress to the animals during the procedure. The protocol was approved by The University of North Dakota Animal care Committee (IACUC#1612-2c)

### Cell culture

The UROtsa parent cells and the six independent As^3+^-transformed isolates and the seven independent Cd^+2^-transformed isolates were cultured in 75 cm^2^ tissue culture flasks in Dulbeco’s modified Eagle’s medium (DMEM) supplemented with 5% vol/vol fetal bovine serum as described previously [10, 11]. The As^3+^ as well as the Cd^2+^-transformed were not cloned and are being referred to as isolates in the study. The cells were sub-cultured at a 1:4 ratio using trypsin-EDTA and the cultures were fed fresh growth medium every three days.

Urospheres (spheroids) were generated by seeding the UROtsa parent and the As^3+^-and Cd^2+^-transformed cell lines at a density of 10^5^ cells in T-25 cm^2^ Ultra-low attachment flasks (Corning Inc., Corning NY). The growth medium consisted of a 1:1 mixture of Dulbecco’s modified Eagles’ medium and Hams’s F-12 growth medium supplemented with selenium (5 ng/ml), insulin (5 μg/ml), transferrin (5 μg/ml), hydrocortisone (36 ng/ml), triiodothyronine (4 pg/ml), and epidermal growth factor (10 ng/ml). The urospheres were harvested after 8 days by centrifugation for RNA isolation, and for injection into immune compromised mice.

### Immunohistochemistry

Tumor tissue from mouse tumor transplants was fixed in 10% neutral-buffered formalin for 16–18 h. The fixed tissue samples were transferred to 70% ethanol and dehydrated in 100% ethanol. The dehydrated tissue samples were cleared in xylene, infiltrated, and embedded in paraffin. Serial sections were cut at 3–5 μm for use in immunohistochemical protocols. Prior to immunostaining, sections were immersed in preheated Target Retrieval Solution (Dako, Carpinteria, CA) and heated in a steamer for 20 min. The sections were allowed to cool to room temperature and immersed into TBS-T for 5 min. The list of antibodies along with their catalogue numbers are included in supplemental materials (S1 Table). The primary antibodies were localized using Dako peroxidase conjugated EnVision plus for rabbit primary antibodies or a Dako peroxidase conjugated EnVision plus dual link antibody for mouse primary antibodies at room temperature for 30 min. Liquid diaminobenzidine (Dako) was used for visualization. Counter staining was performed for 15–30 sec. at room temperature using Ready-to-use Hematoxylin (Dako). Slides were rinsed in distilled water, dehydrated in graded ethanol, cleared in xylene, and cover-slipped. Two pathologists judged the presence and degree of immune-reactivity in the specimens. The scale used was 0 to +3 with 0 indicating no staining, +1 staining of mild intensity, +2 staining of moderate intensity, and +3 staining of strong intensity.

### RNA isolation and real-time PCR analysis

Total RNA was isolated using Tri Reagent (Molecular Research Center, Inc., Cincinnati, OH) as described previously [16]. The expression of various genes was assessed with real-time reverse transcription polymerase chain reaction (RT-PCR) using primers that were purchased from Bio-Rad Laboratories (Hercules, CA) or Qiagen (Valencia, CA). The genes along with the catalog number of the primers are listed in supplemental material (S2 Table). Total RNA was purified from the cells lines and 0.1 μg was subjected to cDNA synthesis using the iScript cDNA synthesis kit (Bio-Rad Laboratories), in a total volume of 20 μL. Real-time RT-PCR was performed using the SYBR Green kit (Bio-Rad Laboratories) with 2 μL cDNA and 0.2 μM primers in a total volume of 20 μL in an iCycler iQ real-time detection system (Bio-Rad Laboratories). Amplification was monitored by SYBR Green fluorescence. Cycling parameters consisted of denaturation at 95°C for 15 sec, annealing at 60°C for 30 sec, and extension at 72°C, which gave optimal amplification efficiency. The resulting levels were normalized to the change in β-actin expression.

### Western blot analysis

Cell culture monolayers were washed with ice cold phosphate-buffered saline. 1X Radio-immunoassay Precipitation Assay (RIPA) lysis buffer supplemented with PMSF, protease inhibitor cocktail and sodium orthovandate (Santa Cruz Biotechnology) was added to the flask and the cells were incubated on ice for five minutes. Following incubation, the cells were scrapped and transferred to a conical tube. The cell suspension was sonicated and the lysate was centrifuged to remove cellular debris. Total cellular protein was separated on a TGX AnyKd SDS-polyacrylamide gel and transferred to a hybond-P polyvinylidene difluoride membrane. Blocking of the membranes were done in TBS-T containing 5% bovine serum albumin (BSA) for 1 hour at room temperature. After blocking, the membranes were probed overnight with the primary antibody diluted in blocking buffer. After washing 3 times in TBS-T, the membranes were incubated with the anti-mouse or anti-rabbit secondary antibody (1:2000) for 1 hour. The blots were visualizes using the Phototope-HRP Western blot detection system (Cell Signaling Technology). The primary antibodies along with their catalogue numbers and dilutions are listed in supplemental material (S3 Table).

### Statistics

Statistical analysis consisted of ANOVA with Tukey *post-hoc* testing performed by Graphpad PRISM 4. All experiments were done in triplicates and the data is plotted as the mean ±SD of triplicate determinations.

## Results

### Expression of basal and luminal markers in tumor transplants generated from As^3+^ and Cd^2+^-transformed UROtsa cells

**Fig 1:**
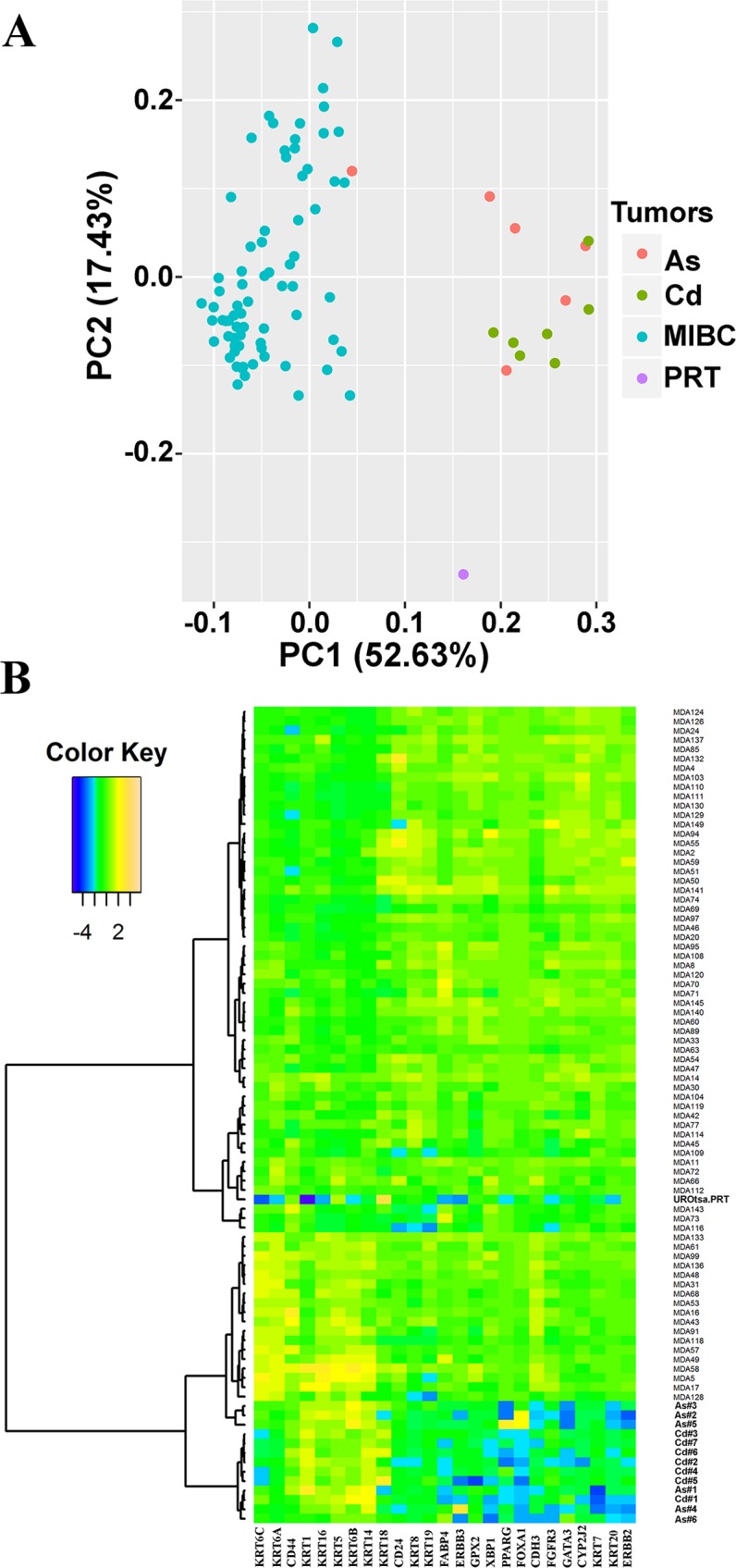
Analysis of gene expression patterns in As^3+^ and Cd^2+^ tumor transplants. (A). Principal Components Analysis (PCA) plot of the MIBCs and UROtsa Cd^2+^ and As^3+^ tumor transplants. Seventy-three muscle invasive bladder cancers (MIBCs), six As^3+^-transformed UROtsa transplants and seven Cd^2+^-transformed UROtsa transplants were plotted onto the first two principal components, PC1 and PC2. The principal components were calculated from the 25 MIBC marker genes. (B). Heat map and the dendrogram on the 25 MIBC markers. Each row of the heat map represents a tumor sample, and each column represents one of the 25 marker genes. The dendrogram was generated using hierarchical clustering based on Pearson’s dissimilarity and Ward’s linkage.

The study by Choi and coworkers [3] identified 25 marker genes that defined basal and luminal genotypes for MIBC. Nine genes were identified to associate with the basal type of MIBC and 16 genes with the luminal type of MIBC (S2 Table). These 25 genes were used to determine if the tumor transplants generated from the UROtsa cells transformed by Cd^2+^ and As^3+^ had gene expression patterns that associated with the basal or luminal subtypes of MIBC. Total RNA was isolated from the tumors generated from seven Cd^2+^ and six As^3+^ independently transformed UROtsa cell lines and analyzed using real time PCR for the 25 marker genes identified by Choi and coworkers [3]. These PCR results were compared to the mRNA expression profiles of the 73 primary MIBCs selected for analysis in the study by Choi and co-workers [3]. To provide a base for comparison of the two datasets, they were both normalized to five housekeeping genes, ACTB (β-actin), B2M (β-2-microglobulin), HPRT1 (hypoxanthine phosphoribosyltransferase 1), RPLP0 (60S acidic ribosomal protein P0), and UBC (ubiquitin C). Following normalization, Principal Component Analysis (PCA) was performed on the integrated data. The first two principal components explained 70.05% of the total variation. As visualized in the PCA plot (Fig 1A), the transplanted tumors generated from the As^3+^ and Cd^2+^-transformed UROtsa cells had similar expression patterns in terms of the 25 marker genes, with all except one UROtsa tumor sample (As#5) that separated from the MIBCs in the first principal component, PC1. In addition to the tumors generated from the transformed UROtsa cells, total RNA isolated from the immortalized, but not tumorigenic, parental UROtsa cell line was also subjected to PCA. The parental UROtsa cell line (PRT) was distinct from all other samples in that it displayed a very low PC2 value. The tumor samples from the As^3+^ and Cd^2+^-transformed cell lines were shown to be at the same levels of the MIBCs in the PC2 axis.

Unsupervised hierarchical clustering was employed to determine the tumor subtypes of the UROtsa transplants. Analysis of the datasets demonstrated that the samples clustered into three distinct groups (Fig 1B). The group located at the top of the dendrogram showed high expression of luminal marker genes and low expression of basal marker genes. The group located at the bottom of the dendrogram was opposite, with a low expression of luminal marker genes and high expression of basal marker genes. The group located in the middle section of the dendrogram showed no obvious up- or down-regulation of either luminal or basal marker genes. The tumor transplants generated by the As^3+^ and Cd^2+^-transformed UROtsa cells all clustered with the basal subtype of MIBC. The basal cluster of the UROtsa tumors was further divided into two subtypes. One subtype consists of only tumors generated from the Cd^2+^ and As^3+^- transformed UROtsa cells. This group includes tumors generated from six isolates of Cd^2+^-transformed cells (Cd#2, Cd#3, Cd#4, Cd#5, Cd#6, and Cd#7) and three isolates of As^3+^-transformed cells (As#2, As#3 and Ad#5). The second subtype includes tumors formed by one isolate of the Cd^2+^-transformed cells (Cd#1) and three isolates of the As^3+^-transformed cells (As#1, As#4 and As#6) which cluster with 18 basal MIBCs. The sample generated from the parental UROtsa cell line have a distinct expression pattern that is not associated with any of the three subtypes.

### Expression and immunohistochemical localization of basal and luminal marker genes in tumors generated from As^3+^ and Cd^2+^-transformed UROtsa cells

**Fig 2:**
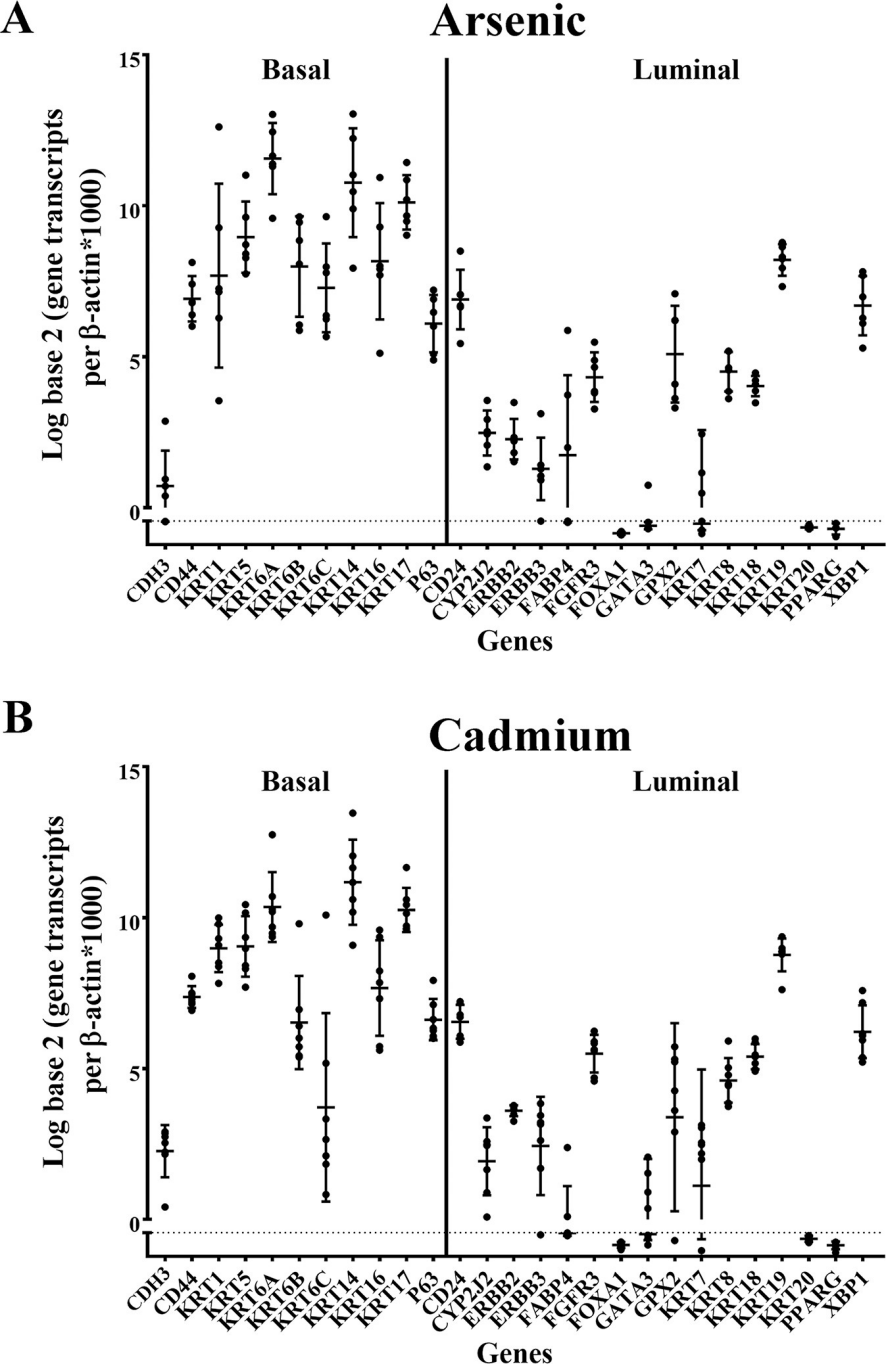
Gene expression analysis of basal and luminal marker genes in tumors produced by As^3+^ and Cd^2+^-transformed cells. Real-time PCR analysis of basal and luminal markers in As^3+^ (A) and Cd^2+^ (B) tumor transplants. The analysis was done in triplicates and is plotted as the mean ± SD. The data is plotted on a log base 2 scale per transcript of β-actin times 1000.

The variation in expression of the 25 MIBC marker genes plus p63 and KRT17 was determined for the tumor transplants generated from the six independent isolates of the As^3+^ and seven independent isolates of the Cd^2+^ -transformed UROtsa cells (Fig 2A and 2B). The expression of tumor protein 63 (TP63), also known as p63 was assessed since this was used in the MIBC study by Choi and co-workers [3] to define a p53-like subset of MIBC. KRT17 was added since it has been associated with basal urothelial cells [17]. Analysis of the gene expression profiles demonstrated a clear trend for higher expression of genes associated with the basal subtype of MIBC. Only a few exceptions were noted to occur for tumors generated from both the As^3+^ and Cd^2+^-transformed cells. The first was that all the tumors displayed a low expression of P-cadherin (CDH3), a marker of basal MIBC. The other was the elevated expression of KRT19 when compared to other markers in the luminal group of genes. While only a subjective observation by the authors’, the variation of expression for each gene within and between the As^3+^ and Cd^2+^ generated tumors was surprising low considering each tumor would be expected to have a variable content of stromal components.

**Fig 3:**
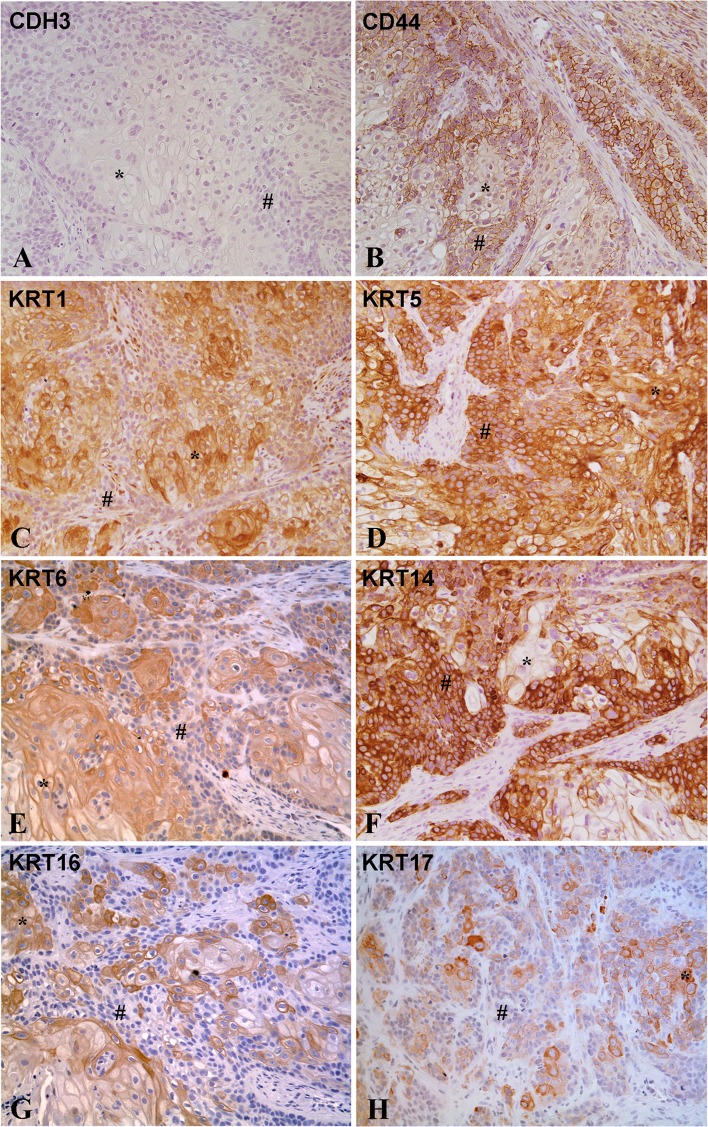
Immuno-histochemical staining for basal markers in tumor transplants generated from As^3+^ -transformed cell line (As#3). (A) CDH3. There is no staining in the differentiated (*) or the less differentiated areas (#) of the tumor transplants. (B). CD44. There is strong membranous staining for CD44 in the less differentiated cells located at the periphery of the tumor nests (#), whereas the well-differentiated cells located in the center of the tumor nests (*) show weak or no staining for CD44. (C). KRT1. The well-differentiated cells (*) in the center of tumor nests are strongly positive for KRT1, whereas the peripheral less differentiated cells (#) show weaker staining. (D). KRT5. The staining for KRT5 is diffuse with strong staining in the well-differentiated (*) as well as less differentiated (#) areas of the tumor. (E). KRT6. The staining for KRT6 is strong in the well-differentiated cells located in the center of the tumor nests (*), whereas the staining is weak to absent in the less differentiated cells (#) located at the periphery of the tumor nests. (F). KRT14. The well-differentiated cells in the center of the tumor nests (*) show weak or no staining for KRT14, whereas the less differentiated peripheral cells (#) are strongly positive. (G). KRT16. The staining for KRT16 is strong in the well-differentiated cells (*) located in the center of the tumor nests, whereas the staining is absent in the less differentiated cells (#) located at the periphery of the tumor nests. (H). KRT17. The staining for KRT17 is strong in the well-differentiated cells (*) located in the center of the tumor nests whereas the staining is weak to absent in the less differentiated cells (#) located at the periphery of the tumor nests. The brown color indicates the presence of the protein whereas the blue/purple color indicates the nuclei that are stained with the counterstain hematoxylin. All images are at a magnification of 200X.

**Fig 4:**
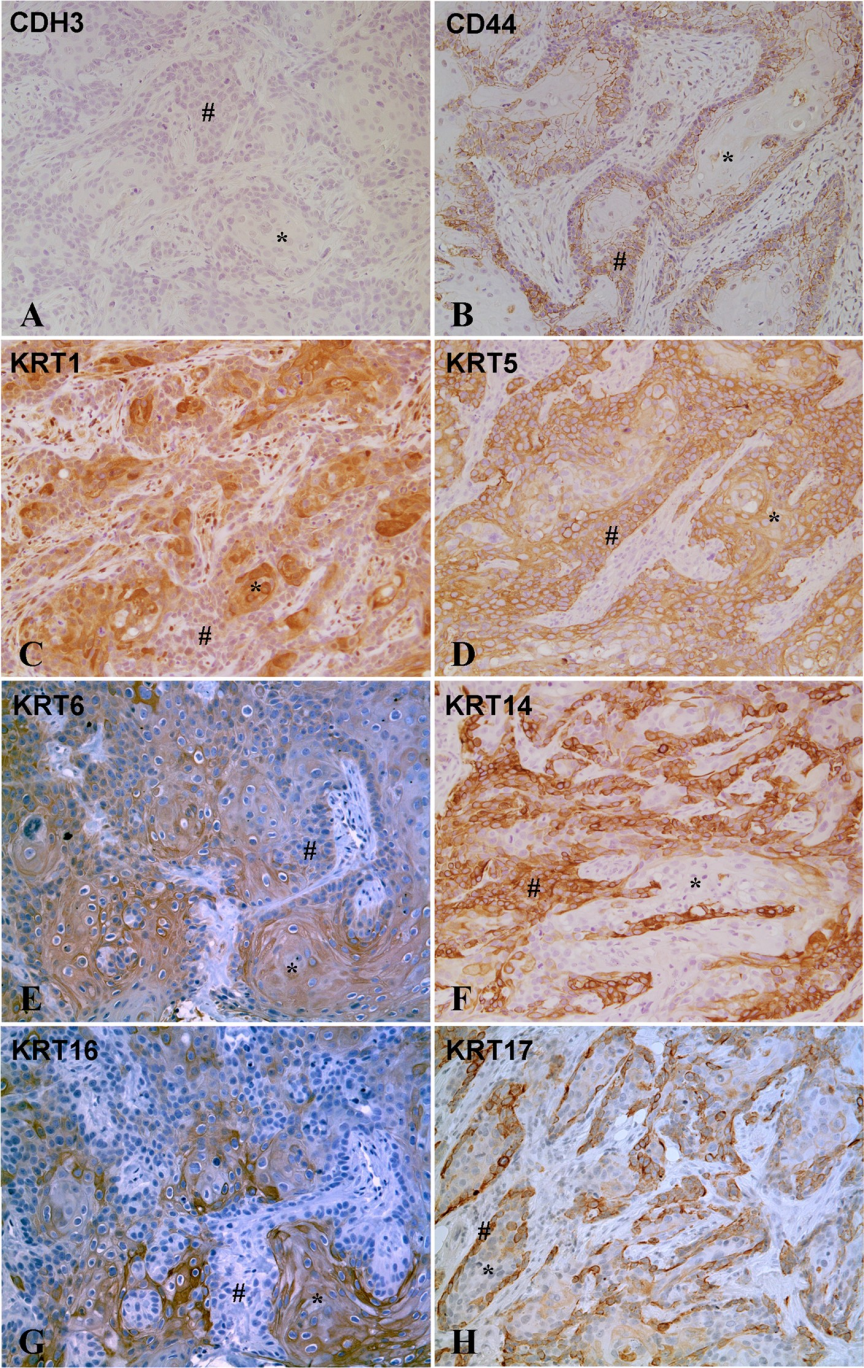
Immuno-histochemical staining for basal markers in tumor transplants generated from Cd^2+^ -transformed cell line (Cd#3). (A). CDH3. There is no staining in the differentiated (*) or the less differentiates areas (#) of the tumor transplants. (B). CD44. There is strong membranous staining for CD44 in the less differentiated cells (#) located at the periphery of the tumor nests, whereas the well-differentiated cells (*) located in the center of the tumor nests show weak or no staining for CD44. (C). KRT1. The well-differentiated cells (*) in the center of tumor nests are strongly positive for KRT1, whereas the peripheral less differentiated cells (#) show weaker staining of KRT1. (D). KRT5. The staining for KRT5 is diffuse with strong staining in the differentiated (*) as well as less differentiated (#) areas of the tumor. (E). KRT6. The staining for KRT6 is strong in the well-differentiated cells (*) located in the center of the tumor nests, whereas the staining is weak to absent in the less differentiated cells (#) located at the periphery of the tumor nests. (F). KRT14. The well-differentiated cells (*) in the center of the tumor nests show weak or no staining for KRT14, whereas the less differentiated peripheral cells (#) are strongly positive for KRT14. (G). KRT16. The staining for KRT16 is strong in the well-differentiated cells (*) located in the center of the tumor nests, whereas the staining is absent in the less differentiated cells (#) located at the periphery of the tumor nests. (H). KRT17. The staining for KRT17 is strong in the less differentiated cells (#) located at the periphery of the tumor nests while the center of the tumor nests containing well-differentiated cells are weakly positive (*). The brown color indicates the presence of the protein whereas the blue/purple color indicates the nuclei that are stained with the counterstain hematoxylin. All images are at a magnification of 200X.

Immunohistochemistry was performed to determine the localization and an estimate of the expression level of basal and luminal proteins in the tumors generated from the As^3+^ and Cd^2+^-transformed cell lines. The staining was positive in all tumors for all basal markers except CDH3, which showed no staining in any of the tumor samples. An example of staining for each basal marker is shown for one tumor generated from the As^3+^ (As#3) and Cd^2+^ (Cd#3)-transformed UROtsa cell line (Figs 3 and 4). The staining for all tumors formed by all the cell lines is shown in supplemental material (S1–S16 Figs). Two independent pathologists assessed the percent of the individual tumors staining positive for the expression of each basal marker as well as the level of expression as judged on a 0 to +3 scale (Table 1). The level of expression, judged as intensity of staining for the basal markers was strong for the tumors, with intensities between +2 and +3 for almost all of the markers. The sole exception among the basal markers was for one tumor (Cd#2) which showed weaker staining for KRT16. The range of staining in the tumors between +2 and +3 reflects the independent judgement of the two pathologists. The two pathologists also judged the percent of the tumor cells stained for each of the basal markers. The basal marker, which displayed the highest number of stained tumor cells, was KRT5, with between 70 and 90% of the tumor cells positive for KRT5. KRT16 showed the lowest number of stained tumor cells, with between 5 and 50% of the cells showing staining. The other basal markers were intermediate between the two extremes and generally followed the pattern of KRT5>KRT1>KRT14≥CD44>KRT6>KRT17>KRT16. The immuno-histochemical staining of the basal markers in the tumor samples reinforced the basal identity of the tumors derived from the As^3+^ and Cd^2+^-transformed UROtsa cells. No correlation was found between individual mRNA levels and the staining intensities or degree of staining of basal markers among the tumor isolates. The KRT6 antibody does not distinguish between the KRT6A, KRT6B and KRT6C isoforms and recognizes protein made by these three genes.

**Table 1:** Immunostaining of basal protein markers in As^3+^ and Cd^2+^ tumor transplants.

Group	KRT1		KRT5		KRT6		KRT14		KRT16		KRT17		CD44	
	INT	%	INT	%	INT	%	INT	%	INT	%	INT	%	INT	%
As#1	3+	60	3+	90	3+	20	3+	90	3+	10	3+	50	3+	70
As#2	3+	50	3+	80	3+	60	3+	80	3+	40	3+	50	3+	60
As#3	3+	50	3+	90	3+	60	3+	80	3+	20	3+	30	3+	30
As#4	3+	40	3+	90	3+	60	3+	90	3+	50	3+	40	3+	70
As#5	3+	40	3+	80	2+	60	3+	30	3+	20	3+	30	3+	30
As#6	3+	70	3+	90	3+	40	3+	90	3+	40	3+	40	3+	50
Cd#1	3+	50	3+	90	3+	60	3+	90	3+	40	3+	70	3+	40
Cd#2	3+	50	3+	70	2+	20	3+	60	1+	<5	3+	30	3+	70
Cd#3	3+	70	3+	90	3+	40	3+	30	2+	10	3+	50	2–3+	40
Cd#4	3+	80	3+	90	3+	40	3+	50	2+	20	3+	40	2–3+	50
Cd#5	3+	80	3+	90	3+	40	3+	60	2+	20	3+	50	2–3+	30
Cd#6	3+	70	3+	90	2+	40	3+	60	2+	10	2–3+	20	2–3+	50
Cd#7	3+	80	3+	90	3+	20	3+	50	2+	10	2–3+	20	2–3+	50

INT; intensity of staining. %; % of cells staining for a marker. 3+; Strong staining. 2+: Moderate staining. 2–3+; Moderate to strong staining. 1+; Weak staining

The histology of the tumors generated from the As^+3^ and Cd^2+^-transformed UROtsa cells showed that the tumors grow as distinct nests of cells demarcated by stroma. These nests were composed of well-differentiated cells in the center with abundant cytoplasm, intercellular bridges and keratinization, and less differentiated basal-like cells at the periphery that were smaller with little cytoplasm and no keratinization. The intensity of staining for several of the basal markers was distinctly different between these areas of the tumors (Table 2 and Figs 3 and 4). KRT1, KRT6, KRT16 and KRT17 showed strong staining in the well-differentiated cells in the center of the tumor nests when compared to the staining in the peripheral tumor cells. This pattern was consistent for the staining of KRT1, KRT6 and KRT16 in all the tumors. The staining of KRT17 in the peripheral areas was similar to the well-differentiated areas in three tumor samples (+3 staining) with the rest of the ten tumors showing no staining for KRT17 in the peripheral less differentiated cells. The staining pattern for CD44 was opposite, with the well-differentiated cells showing only marginal staining for CD44, whereas, those at the periphery showed moderate to strong staining for CD44.

**Table 2:** Localization of basal protein markers in different histological areas of the As^3+^ and Cd^2+^ tumor transplants.

Group	Well-differentiated cells in the center of tumor nests							Peripheral less differentiated cells						
	KRT 1	KRT 5	KRT 6	KRT 14	KRT 16	KRT 17	CD 44	KRT1	KRT5	KRT6	KRT14	KRT16	KRT17	CD 44
As#1	3+	3+	3+	3+	3+	3+	0–1+	+1	3+	0–1+	3+	-	-	3+
As#2	3+	3+	3+	3+	3+	3+	0–1+	+1	3+	0–1+	3+	-	3+	3+
As#3	3+	3+	3+	0–1+	3+	3+	0–1+	+1	3+	0–1+	3+	-	-	3+
As#4	3+	3+	3+	3+	3+	3+	0–1+	+1	3+	0–1+	3+	-	-	3+
As#5	3+	3+	2+	0–1+	3+	3+	0–1+	+1	3+	0–1+	3+	-	3+	3+
As#6	3+	3+	3+	3+	3+	3+	0–1+	+1	3+	0–1+	3+	-	-	3+
Cd#1	3+	3+	3+	3+	3+	3+	0–1+	+1	3+	0–1+	3+	-	-	3+
Cd#2	3+	3+	2+	0–1+	1+	3+	0–1+	+1	3+	0–1+	3+	-	-	3+
Cd#3	3+	3+	3+	0–1+	2+	3+	0–1+	+1	3+	0–1+	3+	-	3+	2–3+
Cd#4	3+	3+	3+	0–1+	2+	3+	0–1+	+1	3+	0–1+	3+	-	-	2–3+
Cd#5	3+	3+	3+	0–1+	2+	3+	0–1+	+1	3+	0–1+	3+	-	-	2–3+
Cd#6	3+	3+	2+	0–1+	2+	3+	0–1+	+1	3+	0–1+	3+	-	-	2–3+
Cd#7	3+	3+	3+	0–1+	2+	3+	0–1+	+1	3+	0–1+	3+	-	-	2–3+

3+; Strong staining. 2+: Moderate staining. 2–3+; Moderate to strong staining. 1+; Weak staining. 0–1+; Negative to weak staining

**Fig 5:**
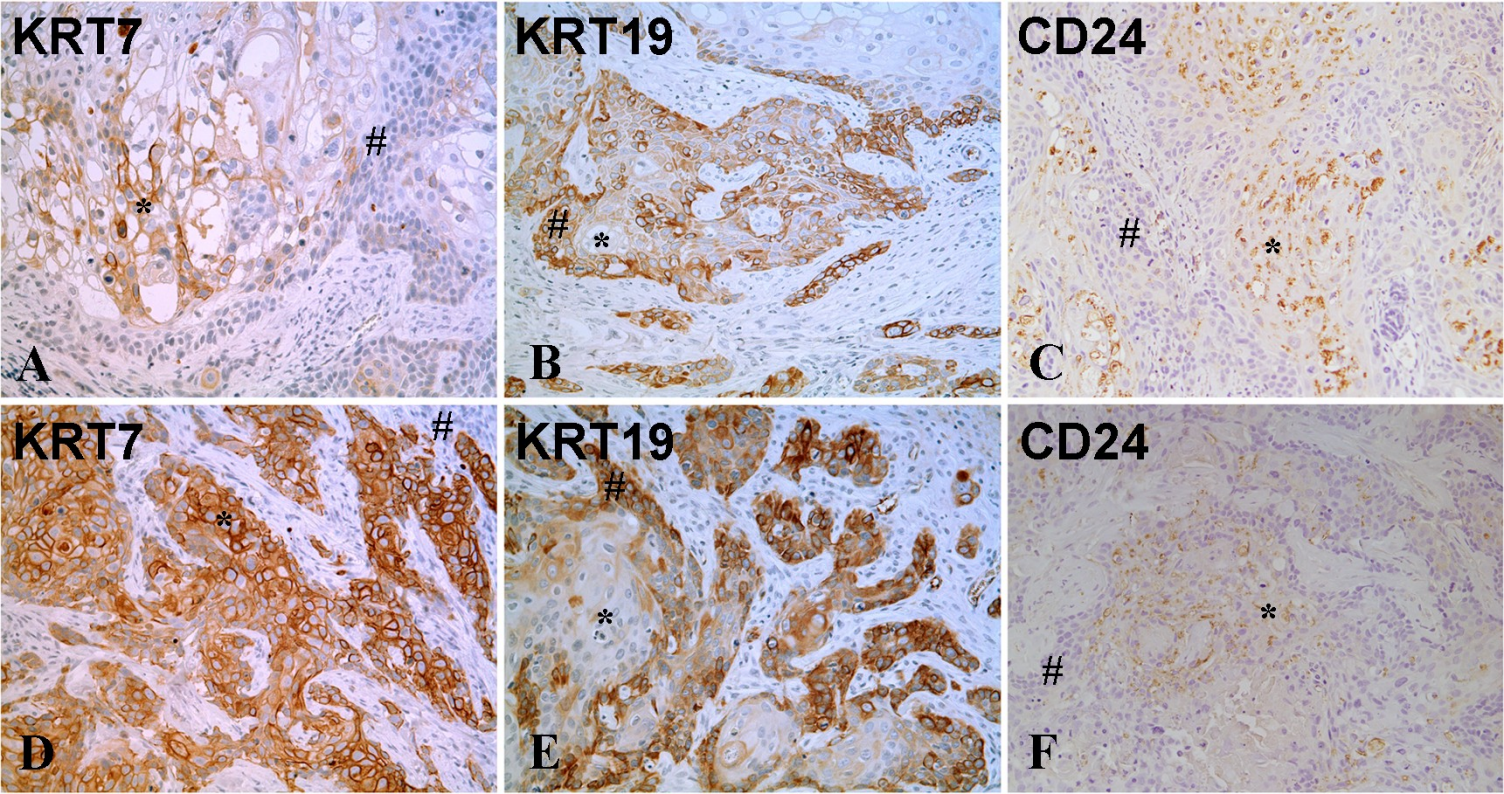
Immuno-histochemical staining for luminal markers in tumor transplants generated from the As^3+^-transformed cell line (As#2) and the Cd^2+^ -transformed cell line (Cd#3). (A-C). As#2. (A). KRT7. There is strong staining in the well-differentiated cells in the center of the tumor nests (*) whereas the peripheral less differentiated cells (#) are negative. (B). KRT19. There is strong staining in the less differentiated cells (#) located at the periphery while the center of the tumor nests with well-differentiated cells (*) are weakly positive. (C). CD24. There is moderate to strong granular staining in the well-differentiated cells (*) located in the center of the tumor nests whereas the peripheral less differentiated cells (#) are negative for CD24. (D-F). Cd#3. (D). KRT7. There is strong staining in the well-differentiated cells in the center of the tumor nests (*) whereas the peripheral less differentiated cells (#) are negative. (E). KRT19. There is strong staining in the less differentiated cells (#) located at the periphery while the center of the tumor nests with well-differentiated cells (*) are weakly positive or negative. (F). CD24. There is moderate to strong focal granular staining in some well-differentiated cells (*) in the center of tumor nests, whereas the peripheral less differentiated cells (#) are negative for CD24. The brown color indicates the presence of the protein whereas the blue/purple color indicates the nuclei that are stained with the counterstain hematoxylin. All images are at a magnification of 200X.

Three of the luminal markers KRT7, KRT19 and CD24 showed staining in the tumor transplants (Fig 5 and Table 3). The staining for all tumor transplants is included in supplemental material (S17–S22 Figs). In three of the tumors (As#1, As#4 and Cd#1), there was no staining for KRT7 and in the rest of the tumors, there was variable staining between 5 and 40%. The intensity of staining, when present, was between +2 and +3. The staining of KRT7 was similar in both the well-differentiated and less differentiated areas of tumor for the tumors generated from Cd^2+^- transformed UROtsa cells. In the As^+3^ derived tumors, there was variable staining for KRT7 between the well and less differentiated areas, with As#2, As#3 and As#6 showing staining in the well-differentiated cells, while As#5 showed staining in the less and well differentiated cells. The staining for KRT19 was present in all tumor samples, with strong staining in 20 to 80% of the cells. Strong staining of KRT19 was localized to the peripheral, less differentiated areas of the tumor nests. CD24 was detected in 1 to 40% of the cells comprising the tumors; with five tumors having 5 or less than 5% of the cells showing positive staining (As#4, Cd#1, Cd#2, Cd#3and Cd#6). The intensity of CD24 staining was between +2 and +3 for all the tumor samples and was present only in the well-differentiated areas of the tumors. There was no staining for the rest of the luminal markers in any of the tumors.

**Table 3:** Expression and localization of luminal protein markers in different histological areas of the As^3+^ and Cd^2+^ tumor transplants.

Group	KRT7		KRT19		CD24		Well differentiated cells in the center of tumor nests			Peripheral less differentiated cells		
							KRT7	KRT19	CD24	KRT7	KRT19	CD24
	INT	%	INT	%	INT	%	INT	INT	INT	INT	INT	INT
As#1	-	0	3+	40	2–3+	40	-	1+	2–3+	-	3+	-
As#2	2+	10	3+	20	2–3+	30	2+	1+	2–3+	-	3+	-
As#3	2+	5	3+	30	2–3+	40	2+	1+	2–3+	-	3+	-
As#4	-	0	3+	80	2–3+	5	-	1+	2–3+	-	3+	-
As#5	3+	40	3+	40	2–3+	30	3+	1+	2–3+	3+	3+	-
As#6	2+	10	3+	40	3+	30	2+	0–1+	3+	-	3+	-
Cd#1	-	0	3+	40	2–3+	2	-	0–1+	2–3+	-	3+	-
Cd#2	3+	40	3+	40	2–3+	1	3+	1+	2–3+	3+	3+	-
Cd#3	3+	40	3+	40	2–3+	5	3+	0–1+	2–3+	3+	3+	-
Cd#4	3+	40	3+	30	2–3+	10	3+	0–1+	2–3+	3+	3+	-
Cd#5	2–3+	30	3+	40	2–3+	20	2–3+	0–1+	2–3+	2–3+	3+	-
Cd#6	2–3+	30	3+	40	2–3+	5	2–3+	-	2–3+	2–3+	3+	-
Cd#7	3+	40	3+	30	2–3+	10	3+	0–1+	2–3+	2–3+	3+	-

INT; Intensity of staining. %; % of cells staining for a marker. 3+; Strong staining. 2+: Moderate staining. 2–3+; Moderate to strong staining. 1+; Weak staining. 0–1+; Negative to weak staining

**Fig 6:**
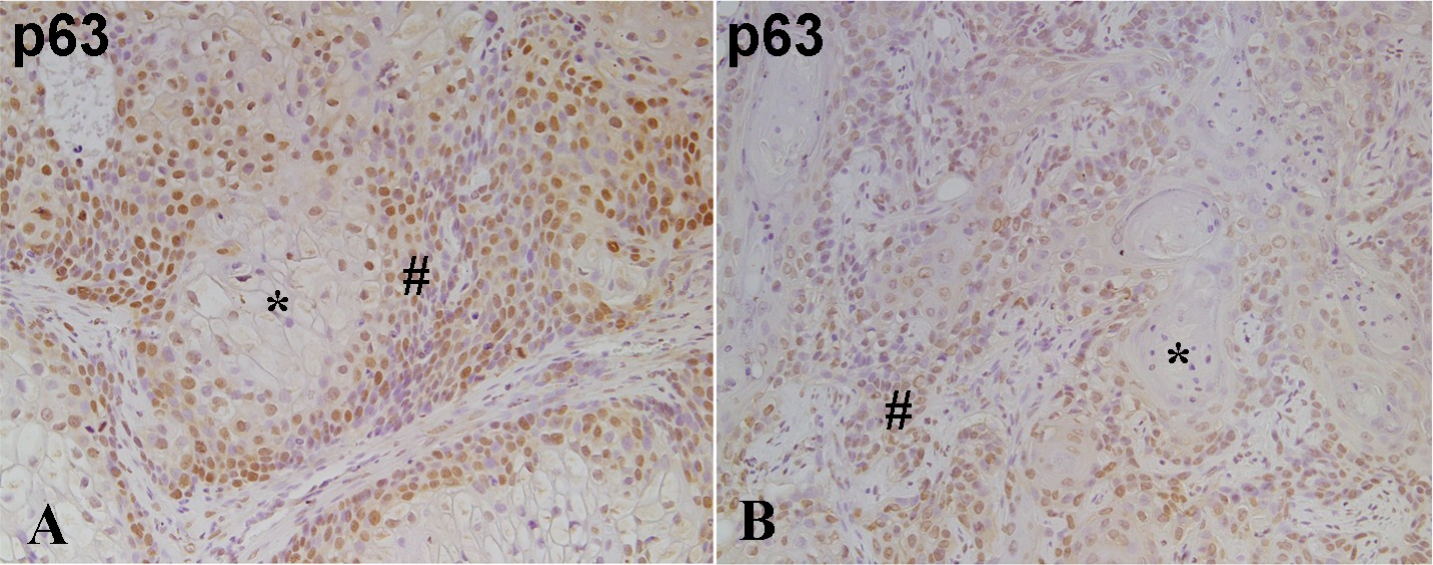
Immuno-histochemical staining for p63 in tumor transplants generated from the As^3+^ and Cd^2+^-transformed line. (A). As#2. There is moderate nuclear staining for p63 in the less differentiated peripheral tumor cells (#) whereas the well-differentiated cells (*) in the center of the tumor nests show no staining. (B). Cd#3. There is moderate nuclear staining for p63 in the less differentiated peripheral tumor cells (#) whereas the well-differentiated cells (*) in the center of the tumor nests show weak or no staining. The brown color indicates the presence of the protein whereas the blue/purple color indicates the nuclei that are stained with the counterstain hematoxylin. All images are at a magnification of 200X.

The expression of p63 was present in all the tumor samples, but varied in both staining intensity and the % of the tumor cells that stained positive. The staining was nuclear and present in 10 to 80% of the tumor cells (Fig 6 for As#2 and Cd#2). Staining for all tumor transplants is included in supplemental materials (S23 and S24 Figs). The majority of staining was in the range of +2 to +3 with the tumor having the lowest number of positive cells having staining at +1 intensity. The staining showed localization of p63 to the less differentiated, periphery areas of the tumor nests.

### Expression of basal and luminal markers in As^3+^ and Cd^2+^-transformed UROtsa cell lines and urospheres

**Fig 7:**
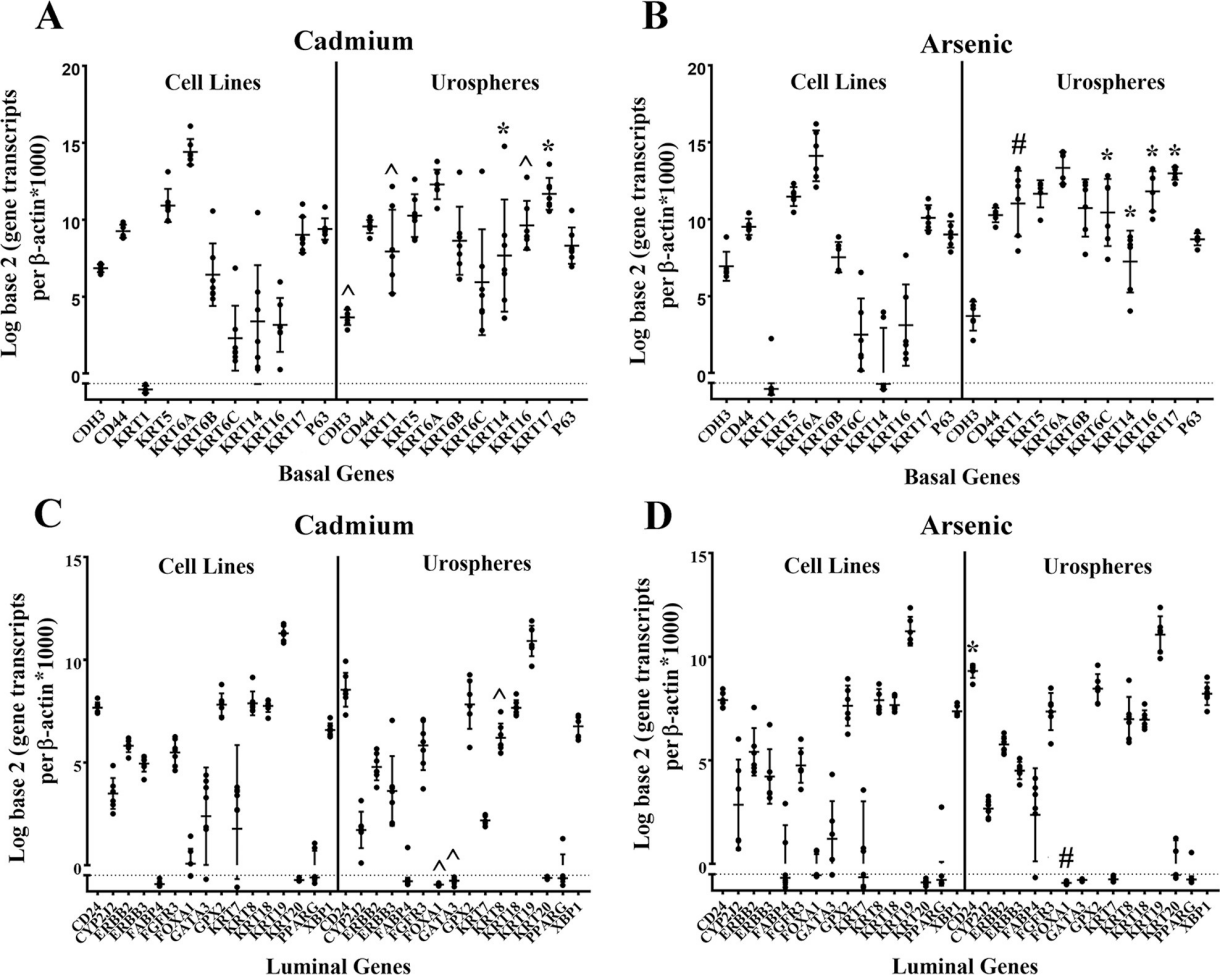
Gene expression analysis of basal and luminal marker genes in the As^3+^ and Cd^2+^-transformed cell lines and their corresponding urospheres. (A and B). Real-time PCR analysis of basal marker genes in Cd^2+^ (A) and As^3+^ (B) transformed cell lines and urospheres. (C and D). Real-time PCR analysis of luminal marker genes in Cd^2+^ (C) and As^3+^ (D)-transformed cell lines and urospheres. Analysis was done in triplicates and is plotted as the mean ± SD. The data is plotted on a log base 2 scale per transcript of β-actin times 1000. * indicates significantly different at *p* < 0.05 from transformed UROtsa cells. # indicates significantly different at *p* < 0.01 from transformed UROtsa cells. ^ indicates significantly different at *p* < 0.001 from transformed UROtsa cells.

The mRNA expression of basal and luminal markers were determined in the As^3+^ and Cd^2+^- transformed cell lines and urospheres isolated from these cell lines. The results of this analysis for 9 basal markers, KRT17 and p63 demonstrated that five markers were altered in the urospheres isolated from the Cd^2+^-transformed cell lines (Fig 7A). The expression of KRT 1 mRNA was below the level of detection in the Cd^2+^-transformed cell lines, but showed a significant increase in expression in the urospheres. The expression levels of KRT 14, 16 and 17 were also significantly increased in the urospheres isolated from the Cd^2+^-transformed cell lines when compared to the cell lines themselves. The expression of CDH3 was lower in the urospheres compared to the cell lines. Similar results were obtained for these markers in the As^3+^-transformed cell lines and urospheres with the exception being KTR6C, which was significantly increased in the urospheres when compared to the As^3+^-transformed cell lines (Fig 7B). An identical analysis for the 16 luminal markers in the Cd^2+^ and As^3+^-transformed cell lines and urospheres showed no significant increase in the expression for 15 of the 16 genes, the exception being CD24, which showed an increase in expression in the urospheres isolated from the As^3+^-transformed cell lines (Fig 7C and 7D). There was a significant decrease in expression of FOXA1, GATA3 and KRT8 in the urospheres isolated from the Cd^+2^-transformed cell line, whereas only FOXA1 was decreased in the urospheres isolated from the As^+3^-transformed cell lines.

The levels of gene expression were also compared among the seven Cd^2+^ and six As^3+^- transformed cell lines to determine if any cell line, or group of cell lines, exhibited a unique profile. The analysis showed that the expression of an individual gene could vary among the cell lines, but that no group of genes distinguished an individual cell line, or group of cell lines, within or between the group of As^3+^ or Cd^2+^- transformed cell lines.

Western blot analysis was also performed on the cell lysates prepared from the UROtsa parent and the As^3+^ and Cd^2+^-transformed cell lines to determine the differences in expression levels of the basal and luminal keratins and CDH3, CD24 and CD44. The data shows that there is a decrease in expression of some of the basal markers in the UROtsa parent cell line when compared to the As^3+^ and Cd^2+^-transformed cell lines (S27–S30 Figs). Among the transformed cell lines, there is a variation in expression with some lines expressing higher protein levels than others. The antibody used for KRT6 recognizes total protein due to sequence homology between the KRT6 isoforms and the protein levels of the individual genes cannot be assessed.

### Squamous differentiation of parent UROtsa cells

**Fig 8:**
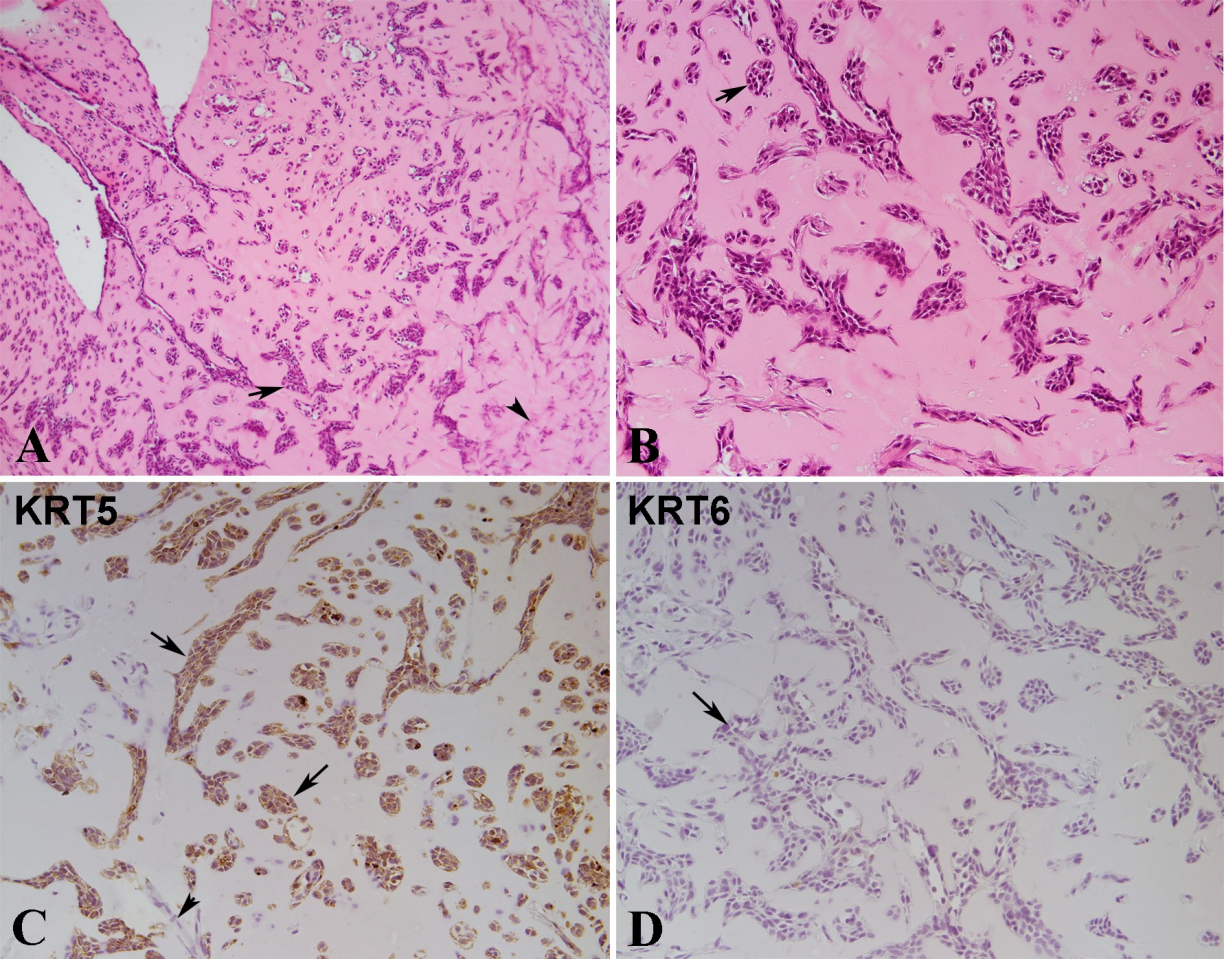
Histology and Immuno-histochemical staining of nodules formed by UROtsa cells injected with matrigel in immune compromised mice. (A). Histology of UROtsa nodule at lower magnification (100X). Epithelial nests in different size and shape and single cells seen in the matrigel (arrows). Spindle stroma cells are present in the right part of the image (arrowhead), mainly in the right part of the image. (B). Histology of UROtsa matrigel nodule at higher magnification (200X). Most of the epithelial nests are irregular in shape with branches or angular formation. A few of epithelial nests are oval or round in shape, similar to the von Brunn’s nests (arrow) in human bladder. Some smaller nests or clusters are composed of only a few epithelial cells. (C). Immunostaining for KRT5. The epithelial nests of various size and some single cells are strongly positive for KRT5 (arrow). The spindle stroma cells are negative for KRT5 (arrowhead). (D). Immunostaining for KRT6. The epithelial nests and single epithelial cells are negative for KRT6 (arrows). All images are at a magnification of 200X unless indicated.

The tumors produced by all the As^3+^ and Cd^2+^-transformed cell lines show prominent evidence of focal squamous differentiation within the more differentiated areas of the tumors [11, 13, 18]. The degree of squamous differentiation in individual tumors can vary from those showing swirls of filamentous material without squamous “pearls” or desmosomes to those showing punctate basophilic granules resembling keratohyaline, prominent intracellular connections and squamous “pearls”. The parental UROtsa cells grown on serum-containing growth media display a morphology resembling the basal layer of the urothelium and show no evidence of squamous differentiation [10]. The UROtsa cells, when grown on a serum-free growth medium, express differentiated features similar to the intermediate layers of stratified urothelium. It is unknown if parental UROtsa parental cells express squamous differentiation when placed *in vivo* under conditions similar to that used to generate tumors in immune compromised mice. To determine this, both the parental UROtsa cells and urospheres isolated from these cells were mixed with Corning matrigel and inoculated subcutaneously under the skin of immune compromised mice. Seven to 10 days later, the subcutaneous nodules were harvested, and processed. Microscopic examination of the H&E stained sections from nodules formed by UROtsa cells as well as the urospheres showed nests of cells within the matrigel with most nests having an irregular shape with branches or angular formations. A representative example of the histology of the nodule from the UROtsa parent cells is shown in Fig 8A and 8B. There was no evidence of squamous differentiation in the nests produced from either the parental UROtsa cells or urospheres. The cells stained positive for KRT 5 confirming their epithelial identity (Fig 8C) and were negative for KRT 6 (Fig 8D), a marker shown previously to identify UROtsa tumor cells with squamous differentiation [13, 18].

### Expression of basal and luminal markers in the parental UROtsa cell line and generated urospheres

**Fig 9:**
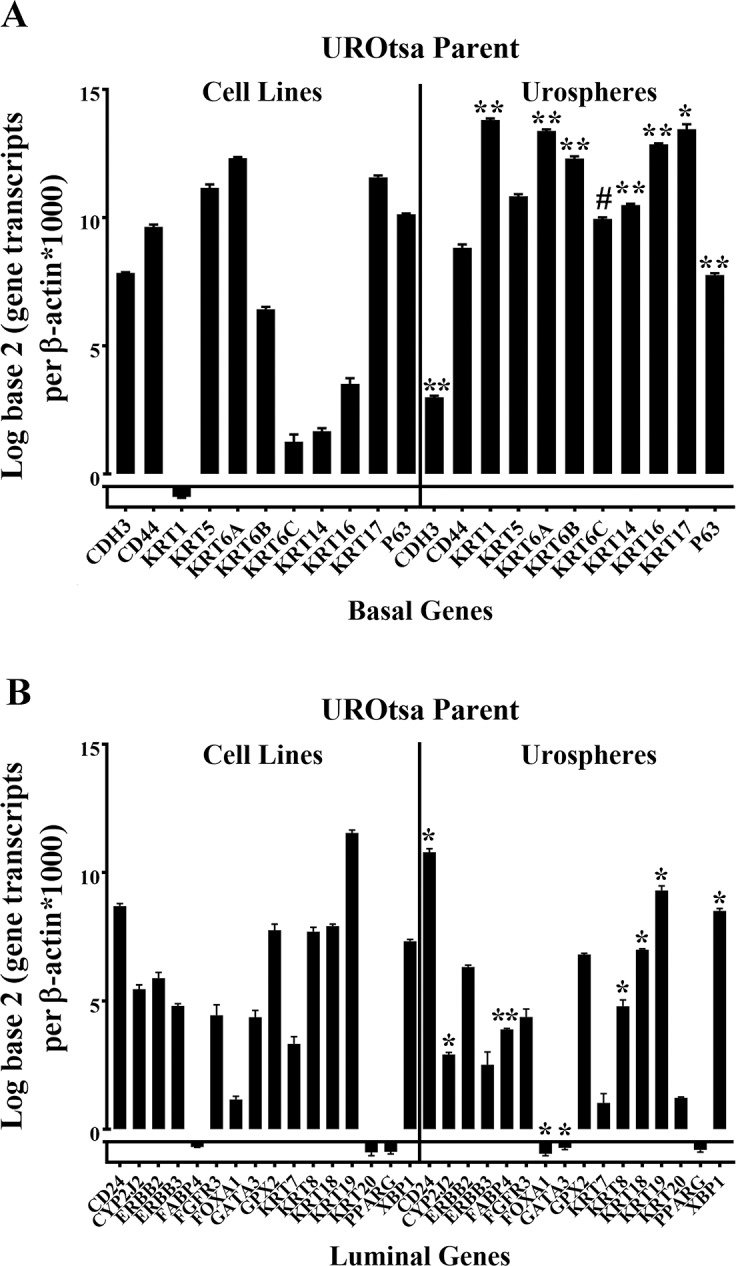
Gene expression analysis of basal and luminal marker genes in the UROtsa parent cell line and urospheres. (A and B). Real-time PCR analysis of basal marker genes (A) and luminal marker genes (B) in the UROtsa parent and the urospheres. The analysis was done in triplicates and is plotted as the mean ±SD. The data is plotted on a log base 2 scale per transcript of β-actin times 1000. * indicates significantly different at *p* < 0.05 from parent UROtsa cells. # indicates significantly different at *p* < 0.01 from parent UROtsa cells. ** indicates significantly different at *p* < 0.0001 from parent UROtsa cells.

The expression of 9 basal (plus KRT17 and P63) and 16 luminal markers was assessed in the parental UROtsa cells and urospheres isolated from the parental cell line. The results for the basal markers showed that seven genes (KRT1, KRT6A, KRT6B, KRT6C, KRT14, KRT16 and KRT17) were significantly elevated in the urospheres compared to the parental cell line (Fig 9A). Five of these seven basal markers were also those elevated in the urospheres isolated from the As^3+^ and Cd^2+^-transformed cell lines, the exception being the KRT6A and KRT6B genes, which showed no change in expression in the transformed cells (Figs 9A, 7A & 7B). There was a significant decrease in the expression levels of CDH3 and P63 in the parental urospheres when compared to the UROtsa cell line. For the luminal markers, there was a significant increase in the expression of CD24, FABP4 and XBP1 in the urospheres when compared to the UROtsa cell line (Fig 9B), whereas there was a significant decrease in the expression of CYP2J2, FOXA1, GATA3, KRT8, KRT18 and KRT19 in the urospheres when compared to the UROtsa cell line (Fig 9B). The expression of FOXA1, GATA3 and KRT8 was also decreased in the urospheres isolated from the transformed cell lines (Fig 7C and 7D).The expression of the rest of the markers was not significantly altered between the urospheres and the UROtsa parent cell line.

## Discussion

The first goal of this study was to determine if the tumor transplants generated from As^3+^ and Cd^2+^-transformed UROtsa cells display a gene expression pattern similar to the basal or luminal subtypes identified by Choi and coworkers [3] for MIBC. The expression of mRNA for the tumors derived from the six isolates of As^3+^ and seven isolates of Cd^2+^-transformed UROtsa cells indicated an identity with the basal subtype of MIBC using two different approaches based on mRNA expression. The first was to compare the expression of mRNA for basal and luminal markers in the UROtsa-derived tumor transplants with the same 25 marker genes in the 73 primary MIBCs selected for analysis in the study by Choi and coworkers [3]. Unsupervised hierarchical clustering demonstrated a strong identity of the UROtsa-derived tumors with the basal subtype. The second approach simply compared the expression of mRNA between the basal and luminal markers for the tumors derived from the six isolates of As^3+^ and seven isolates of Cd^2+^-transformed cells and demonstrated that the expression of basal markers was elevated compared to the luminal markers. While supporting the results from the hierarchical clustering, there is some concern generated due to the numerous keratin genes in the basal subtype and the number of transcription factors associated with the luminal subtype. The keratins may have high levels of expression due to their association with the cytoskeleton while transcription factors can exert their influence at low expression levels. However, two of the three keratins associated with the luminal subtype did show only modest (KRT7) or no expression (KRT20) in the tumor transplants. The final evidence that the UROtsa-derived tumors display a basal gene signature was the immunohistochemical expression of the basal and luminal markers. With the exception of CDH3, there was strong staining for all the other basal markers in the tumor transplants. In contrast, only three luminal markers showed immuno-staining in the tumor transplants (KRT7, KRT19, and CD24). Thus, tumors derived from As^3+^ and Cd^2+^-transformed UROtsa cells have an expression pattern consistent with that found for the basal subtype of MIBCs.

We compared the gene expression profiles of the UROtsa-derived tumors to the MIBC study since it divided the tumors into the basal and luminal subsets only, simplifying the comparison. The authors have no evidence at present to suggest that the UROtsa-derived tumors are muscle invasive. However, previous studies have shown that the UROtsa-derived tumors have a prominent focal component of squamous differentiation [11]. The study by Choi and coworkers [3] showed basal MIBCs with significantly enriched squamous features, while luminal tumors had no squamous features. The high molecular weight keratins (KRT5, KRT6 and KRT14) that characterize basal MIBCs are also enriched in a lethal “squamous cell carcinoma” MIBC subtype identified by another independent research group [2]. This lethal subtype of MIBCs identifies with the basal subtype in the study of Choi and coworkers [3]. The immunohistochemical study by Sjodahl and coworkers [19] divided urothelial carcinomas into 4 subgroups; urobasalA (uroA), urobasalB (uroB), squamous cell carcinoma-like (SCCL), and genomically unstable (GU). The tumors produces by the As^3+^ and Cd^2+^-transformed UROtsa cells showed the most similarity to the SCCL tumors with some shared characteristics with uroB tumors. Tumors produced by the transformed UROtsa cells express EGFR and E-cadherin [20, 21], markers expressed in the SCCL tumors. A similar elevated expression of high molecular weight keratins also characterized a bladder cancer “squamous cluster” [22] that was later shown by Choi and coworkers [3] to agree with their basal subset of MIBCs. The link between squamous features and aggressive behavior is consistent with other recent studies [23, 24]. Overall, the tumors produced by the transformed UROtsa cells appear to associate with the more aggressive types of urothelial cancer.

The association of the UROtsa-derived tumors with more aggressive types of urothelial cancer could be influenced by several factors. One factor is that there is limited influence of immune cells on the UROtsa-derived tumors since the tumors are transplanted using immune compromised mice, removing important stromal interaction with the tumors. The histology of the tumors is consistent with a stroma composed of fibroblasts. A second factor is that the tumors are derived from an UROtsa parent cell line that was immortalized using the SV40 large T-antigen [25, 26]. The effect of this on the character of the UROtsa-derived tumors is unknown, but may influence genomic stability due to its interaction with p53. The inhibition of p53 function may lead to a more aggressive tumor genotype. The expression of p63, a member of the p53 family [27] was determined in the UROtsa-derived tumors and was found to be expressed in all the tumors. The histology of the UROtsa-derived tumors was also informative, characterized by nests of tumor cells demarcated by surrounding mouse stromal cells. These tumor nests were composed of two distinct cell types, well-differentiated tumor cells in the center of the tumor nests and less differentiated cells at the periphery. The features of squamous differentiation were very pronounced in the center of the tumor nests and far less noticeable at the periphery of the tumors. Immunohistochemical localization showed that KRT1, KRT6, and KRT16 were highly elevated in the well-differentiated cells in the center of the tumor rests when compared to the less differentiated cells at the periphery. This could be due to hypoxia since the cells in the center of the nests may not be as vascularized as cells at the periphery of the nests. This may be of interest in studies on tumor progression since it provides a system composed of specifically located less- and well-differentiated tumor cells.

The expression of mRNA for the basal and luminal markers were also assessed in the As^3+^ and Cd^2+^-transformed cell lines and urospheres isolated from each of the cell lines. The gene expression signature of the transformed UROtsa cell lines identified the most with the basal gene signature, with this identity enhanced in urospheres isolated from the cell lines. There was increased expression of KRT1, KRT6C, KRT14 and KRT16 in the urospheres compared to the transformed cell lines and these keratin genes were also elevated in the MIBCs. These findings are consistent with the notion that the urospheres are the cancer initiating cells (CICs) that drive tumor formation in immune compromised mice. The final goal of the study was to determine the gene signature of the parental UROtsa cells and the urospheres derived from them and compare them with expression patterns seen in the transformed cell lines, tumors derived from these lines, and the MIBCs. The major finding from this analysis was that urospheres from the parental UROtsa cells showed increased expression of KRT1, KRT6, KRT14, KTR16 and KRT17 compared to the parental cell lines. The increased expression of these five keratins was also found in urospheres isolated from the transformed cells lines and in tumors derived from these cell lines. Taken together, these keratins might be an indicator of UCs with, or destined to progress to, tumors with squamous differentiation. The findings suggest that the urospheres isolated from the parental UROtsa cells are those that undergo transformation and gain the ability to form tumors after exposure to As^3+^ or Cd^2+^.

The study also demonstrated that the parental UROtsa cells injected subcutaneously with matrigel in immune compromised mice, formed clusters of epithelial cells with no evidence of squamous differentiation. Immuno-staining with an antibody to KRT5 confirmed their epithelial identity and a lack of staining with a KRT6 antibody was consistent with a lack of squamous differentiation. The clusters of cells formed by the UROtsa parental cells have a morphology similar to Von Brunn’s nest, which in the bladder are groups of urothelial cells within the lamina propria and submucosa that are formed from budding of the surface mucosa and are considered to be normal urothelium [28, 29]. The formation of the clusters of the urothelial cells following the injection of the parental UROtsa cells into immune compromised mice is not an indicator of tumor formation. The mixture of the matrigel and growth media supports the growth of the subcutaneously injected cells for up to 13 days, as noted by the formation of a visible nodule under the skin, after which the cells within the nodule become necrotic, and the nodule rapidly disappears. Mice injected with the UROtsa parent have been monitored for six months with no evidence of tumor formation or any evidence of cells at the injection site. In contrast, the As^3+^ and Cd^2+^-transformed UROtsa cells follow the same pattern over the initial 10 to 13 days, but following disappearance of the nodule, the nodule reforms and continues to grow into a tumor.

In conclusion, our results suggest that the As^3+^ and Cd^2+^ -transformed cell lines and their corresponding tumors express genes similar to the set expressed by the basal subtype of MIBC. The expression of these genes particularly the keratins is increased in the stem/progenitor cells isolated from these transformed cell lines, and may be involved in the development of squamous differentiation seen in some of the MIBCs.

## Supporting information

S1 Fig Immuno-histochemical staining for CDH3 in As^+3^-transformed subcutaneous tumor transplants. (A-F). Staining for As#1, As#2, As#3, As#4, As#5 and As#6 respectively. There is no staining for CDH3 in the well-differentiated cells (*) in the center of tumor nests as well as the peripheral less differentiated cells (#). All images are at a magnification of 200X.
(TIF)

S2 Fig Immuno-histochemical staining for CDH3 in Cd^+2^-transformed subcutaneous tumor transplants. (A-G). Staining for Cd#1, Cd#2, Cd#3, Cd#4, Cd#5, Cd#6 and Cd#7 respectively. There is no staining for CDH3 in the well-differentiated cells (*) in the center of tumor nests as well as the peripheral less differentiated cells (#). All images are at a magnification of 200X.
(TIF)

S3 Fig Immuno-histochemical staining for CD44 in As^+3^-transformed subcutaneous tumor transplants. (A-F). Staining for As#1, As#2, As#3, As#4, As#5 and As#6 respectively. There is strong membranous staining for CD44 in the less differentiated cells located at the periphery of the tumor nests (#), whereas the well differentiated cells located in the center of the tumor nests (*) show weak or no staining for CD44. All images are at a magnification of 200X.
(TIF)

S4 Fig Immuno-histochemical staining for CD44 in Cd^+2^-transformed subcutaneous tumor transplants. (A-G). Staining for Cd#1, Cd#2, Cd#3, Cd#4, Cd#5, Cd#6 and Cd#7 respectively. There is moderate to strong membranous staining for CD44 in the less differentiated cells located at the periphery of the tumor nests (#), whereas the well differentiated cells located in the center of the tumor nests show weak or no staining for CD44. All images are at a magnification of 200X.
(TIF)

S5 Fig Immuno-histochemical staining for KRT1 in As^+3^-transformed subcutaneous tumor transplants. (A-F). Staining for As#1, As#2, As#3, As#4, As#5 and As#6 respectively. The well-differentiated cells (*) in the center of tumor nests are strongly positive for CK1, whereas the peripheral less differentiated cells (#) show weaker staining of CK1. All images are at a magnification of 200X.
(TIF)

S6 Fig Immuno-histochemical staining for KRT1 in Cd^+2^-transformed subcutaneous tumor transplants. (A-G). Staining for Cd#1, Cd#2, Cd#3, Cd#4, Cd#5, CD#6 and Cd#7 respectively. The well- differentiated cells (*) in the center of tumor nests are strongly positive for CK1, whereas the peripheral less differentiated cells (#) show weaker staining of CK1. All images are at a magnification of 200X.
(TIF)

S7 Fig Immuno-histochemical staining for KRT5 in As^+3^-transformed subcutaneous tumor transplants. (A-F). Staining for As#1, As#2, As#3, As#4, As#5 and As#6 respectively. The staining for KRT5 is diffuse with strong staining in the differentiated (*) as well as less differentiated (#) areas of the tumor. All images are at a magnification of 200X.
(TIF)

S8 Fig Immuno-histochemical staining for KRT5 in Cd^+2^-transformed subcutaneous tumor transplants. (A-G). Staining for Cd#1, Cd#2, Cd#3, Cd#4, Cd#5, Cd#6 and Cd#7 respectively. The staining for KRT5 is diffuse with strong staining in the differentiated (*) as well as less differentiated (#) areas of the tumor. All images are at a magnification of 200X.
(TIF)

S9 Fig Immuno-histochemical staining for KRT6 in As^+3^-transformed subcutaneous tumor transplants. (A-F). Staining for As#1, As#2, As#3, As#4, As#5 and As#6 respectively. The staining for KRT6 is strong in the well-differentiated (*) cells located in the center of the tumor nests with squamous features, whereas the staining is weak to absent in the less differentiated cells (#) located at the periphery of the tumor nests. All images are at a magnification of 200X.
(TIF)

S10 Fig Immuno-histochemical staining for KRT6 in Cd^+2^-transformed subcutaneous tumor transplants. (A-G). Staining for Cd#1, Cd#2, Cd#3, Cd#4, Cd#5, Cd#6 and Cd#7 respectively The staining for KRT6 is strong in the well-differentiated cells located in the center of the tumor nests with squamous features, whereas the staining is weak to absent in the less differentiated cells (#) located at the periphery of the tumor nests. All images are at a magnification of 200X.
(TIF)

S11 Fig Immuno-histochemical staining for KRT14 in As^+3^-transformed subcutaneous tumor transplants. (A-F). Staining for As#1, As#2, As#3, As#4, As#5 and As#6 respectively. For As#1, As#2, As#4, and As#6, the staining for KRT14 is diffuse with strong staining in the differentiated (*) as well as less differentiated (#) area of the tumor. For As#3 and As#5, the well differentiated cells in the center of the tumor nests (*) show weak or no staining for KRT14, whereas the less differentiated peripheral cells (#) are strongly positive for KRT14. All images are at a magnification of 200X.
(TIF)

S12 Fig Immuno-histochemical staining for KRT14 in Cd^+2^-transformed subcutaneous tumor transplants. (A-G). Staining for Cd#1, Cd#2, Cd#3, Cd#4, Cd#5, Cd#6 and Cd#7 respectively. For Cd#1, the staining for KRT14 is diffuse with strong staining in the differentiated (*) as well as less differentiated (#) area of the tumor. For Cd#2, Cd#3, Cd#4, Cd#5, Cd#6 and Cd#7, the well-differentiated cells (*) in the center of the tumor nests show weak or no staining for KRT14, whereas the less differentiated peripheral cells (#) show strong staining for KRT14. All images are at a magnification of 200X.
(TIF)

S13 Fig Immuno-histochemical staining for KRT16 in As^+3^-transformed subcutaneous tumor transplants. (A-F). Staining for As#1, As#2, As#3, As#4, As#5 and As#6 respectively. The staining for KRT16 is moderate to strong in the well-differentiated cells (*) located in the center of the tumor nests with squamous features, whereas the staining is weak to absent in the less differentiated cells (#) located at the periphery of the tumor nests. All images are at a magnification of 200X.
(TIF)

S14 Fig Immuno-histochemical staining for KRT16 in Cd^+2^-transformed subcutaneous tumor transplants. (A-G). Staining for Cd#1, Cd#2, Cd#3, Cd#4, Cd#5, Cd#6 and Cd#7 respectively. The staining for KRT16 is strong in Cd#1, whereas in Cd#3, Cd#4, Cd#5, Cd#6 and Cd#7, the staining for KRT16 is moderate. In Cd#2, the staining is absent for KRT16. The staining is in the well-differentiated cells (*) located in the center of the tumor nests with squamous features, whereas the staining is absent in the less differentiated cells (#) located at the periphery of the tumor nests. All images are at a magnification of 200X.
(TIF)

S15 Fig Immuno-histochemical staining for KRT17 in As^+3^-transformed subcutaneous tumor transplants. (A-F). Staining for As#1, As#2, As#3, As#4, As#5 and As#6 respectively. The staining for KRT17 is strong in the well-differentiated cells (*) located in the center of the tumor nests with squamous features. In tumors formed by As#2 (B) and As#5(E), there is also strong staining in the less differentiated basal-like cells (#) located at the periphery of the tumor nests. All images are at a magnification of 200X.
(TIF)

S16 Fig Immuno-histochemical staining for KRT17 in Cd^+2^-transformed subcutaneous tumor transplants. (A-G). Staining for Cd#1, Cd#2, Cd#3, Cd#4, Cd#5, Cd#6 and Cd#7 respectively. The staining for KRT17 is strong in the well-differentiated cells (*) located in the center of the tumor nests with squamous features. In tumors formed by Cd#3 (C) and Cd#4 (D), there is also strong staining in the less differentiated basal-like cells (#) located at the periphery of the tumor nests. All images are at a magnification of 200X.
(TIF)

S17 Fig Immuno-histochemical staining for KRT7 in As^+3^-transformed subcutaneous tumor transplants. (A-F). Staining for As#1, As#2, As#3, As#4, As#5 and As#6 respectively. There is no staining in the tumors for As#1 and As#4 (A and D). For As#2 (B), As#3 (C) and As#6 (F), the expression is focal, whereas for As#5 (E), the staining is strong and diffuse. In all the positively stained sections, the staining was localized to the well-differentiated cells (*) in the center of the tumor nests. There is no staining in the less differentiated basal-like cells (#) located at the periphery of the tumor nests. All images are at a magnification of 200X.
(TIF)

S18 Fig Immuno-histochemical staining for KRT7 in Cd^+2^-transformed subcutaneous tumor transplants. (A-G). Staining for Cd#1, Cd#2, Cd#3, Cd#4, Cd#5, Cd#6 and Cd#7 respectively. There was no staining in the tumor for Cd#1(A). For Cd#2, Cd#3, Cd#4, Cd#5, Cd#6 and Cd#7 (B-G), there is strong staining that is diffuse and localized to the well differentiated cells in the center of the tumor nests (*). There is no staining in the less differentiated basal-like cells (#) located at the periphery of the tumor nests. All images are at a magnification of 200X.
(TIF)

S19 Fig Immuno-histochemical staining for KRT19 in As^+3^-transformed subcutaneous tumor transplants. (A-F). Staining for As#1, As#2, As#3, As#4, As#5 and As#6 respectively. The expression of KRT19 is strong in all the tumor transplants with staining mainly in the less differentiated cells (#) located at the periphery of the tumor nests whereas the well differentiated cells (#) area either weakly positive or negative for the staining of KRT19. All images are at a magnification of 200X.
(TIF)

S20 Fig Immuno-histochemical staining for KRT19 in Cd^+2^-transformed subcutaneous tumor transplants. (A-G). Staining for Cd#1, Cd#2, Cd#3, Cd#4, Cd#5, Cd#6 and Cd#7 respectively. The expression of KRT19 is strong in all the tumor transplants with staining mainly in the less differentiated cells (#) located at the periphery of the tumor nests whereas the well differentiated cells (*) are either weakly positive or negative for the staining of KRT19. All images are at a magnification of 200X.
(TIF)

S21 Fig Immuno-histochemical staining for KRT20 in As^+3^-transformed subcutaneous tumor transplants. (A-F). Staining for As#1, As#2, As#3, As#4, As#5 and As#6 respectively. There is no staining for KRT20 in the tumor transplants. * indicates the well-differentiated areas whereas # indicates the less differentiated areas of the tumor. All images are at a magnification of 200X.
(TIF)

S22 Fig Immuno-histochemical staining for KRT20 in Cd^+2^-transformed subcutaneous tumor transplants. (A-G). Staining for Cd#1, Cd#2, Cd#3, Cd#4, Cd#5, Cd#6 and Cd#7 respectively. There is no staining for KRT20 in the tumor transplants. * indicates the well-differentiated areas whereas # indicates the less differentiated areas of the tumor. All images are at a magnification of 200X.
(TIF)

S23 Fig Immuno-histochemical staining for CD24 in As^+3^-transformed subcutaneous tumor transplants. (A-F). Staining for As#1, As#2, As#3, As#4, As#5 and As#6 respectively. The staining for CD24 is granular with moderate to strong staining in the well-differentiated cells (*) located in the center of tumor nests, whereas the staining is absent in the less differentiated cells (#) located at the periphery of the tumor nests. The staining in As#4 is focal. All images are at a magnification of 200X.
(TIF)

S24 Fig Immuno-histochemical staining for CD24 in Cd^+2^-transformed subcutaneous tumor transplants. (A-G). Staining for Cd#1, Cd#2, Cd#3, Cd#4, Cd#5, Cd#6 and Cd#7 respectively. The staining for CD24 in Cd#1, Cd#2, Cd#3 and Cd#6 is focal and granular in some of the well-differentiated cells (*)in the center of the tumor nests, whereas most of the tumor cells are negative for CD24. For Cd#4,Cd#5 and Cd#7, the staining for CD24 is granular with moderate to strong staining in the well differentiated cells (*) located in the center of tumor nests, whereas the staining is absent in the less differentiated cells (#) located at the periphery of the tumor nests. All images are at a magnification of 200X.
(TIF)

S25 Fig Immuno-histochemical staining for p63 in As^+3^-transformed subcutaneous tumor transplants. (A-F). Staining for As#1, As#2, As#3, As#4, As#5 and As#6 respectively. There is moderate to strong nuclear staining for p63 in the less differentiated peripheral tumor cells (#), whereas the well-differentiated cells (*) in the center of the tumor nests showed weak or no staining for p63. For As#4 and As#6, there is strong nuclear staining in both the differentiated as well as the less differentiated peripheral tumor cells. All images are at a magnification of 200X.
(TIF)

S26 Fig Immuno-histochemical staining for p63 in Cd^+2^-transformed subcutaneous tumor transplants. (A-G). Staining for Cd#1, Cd#2, Cd#3, Cd#4, Cd#5, Cd#6 and Cd#7 respectively. In Cd#1, Cd#2, Cd#4 and Cd#6, there is moderate to strong nuclear staining of p63 in the less differentiated peripheral tumor cells, whereas the well-differentiated cells in the center of the tumor nests showed weak or no staining for p63. For Cd#5 and Cd#7, the staining is weak to moderate in the less differentiated peripheral tumor cells. All images are at a magnification of 200X.
(TIF)

S27 Fig Western blot analysis of basal genes in UROtsa parent and As3+-transformed UROtsa cell lines. (A-I). Western blots for KRT1, KRT5, KRT6, KRT14, KRT16, KRT17, CDH3, CD44 and β-actin. Integrated optical density (IOD) of each band was normalized to that of β-actin. * indicates significantly different at p < 0.05 from parent UROtsa cells.
(TIF)

S28 Fig Western blot analysis of basal genes in UROtsa parent and Cd2+-transformed UROtsa cell lines. (A-I). Western blots for KRT1, KRT5, KRT6, KRT14, KRT16, KRT17, CDH3, CD44 and β-actin. Integrated optical density (IOD) of each band was normalized to that of β-actin. * indicates significantly different at p < 0.05 from parent UROtsa cells.
(TIF)

S29 Fig Western blot analysis of luminal genes in UROtsa parent and As3+-transformed UROtsa cell lines. (A-F). Western blots for KRT7, KRT8, KRT18, KRT19, CD24 and β-actin. Integrated optical density (IOD) of each band was normalized to that of β-actin. * indicates significantly different at p < 0.05 from parent UROtsa cells.
(TIF)

S30 Fig Western blot analysis of luminal genes in UROtsa parent and Cd2+-transformed UROtsa cell lines. (A-F). Western blots for KRT7, KRT8, KRT18, KRT19, CD24 and β-actin. Integrated optical density (IOD) of each band was normalized to that of β-actin. * indicates significantly different at p < 0.05 from parent UROtsa cells.
(TIF)

S1 Table Antibodies used in Immunohistochemical studies. (DOCX)

S2 Table List of genes identified with basal and luminal type of muscle invasive bladder cancer (MIBC). (DOCX)

S3 Table Antibodies used in Western analysis. (DOCX)
