# Supplementary figures and images for: The urothelial cell line UROtsa transformed by arsenite and cadmium display basal characteristics associated with muscle invasive urothelial cancers
Source: PLoS One. 2018 Dec 14;13(12):e0207877. doi: 10.1371/journal.pone.0207877 (PMC6294394; doi:10.1371/journal.pone.0207877)

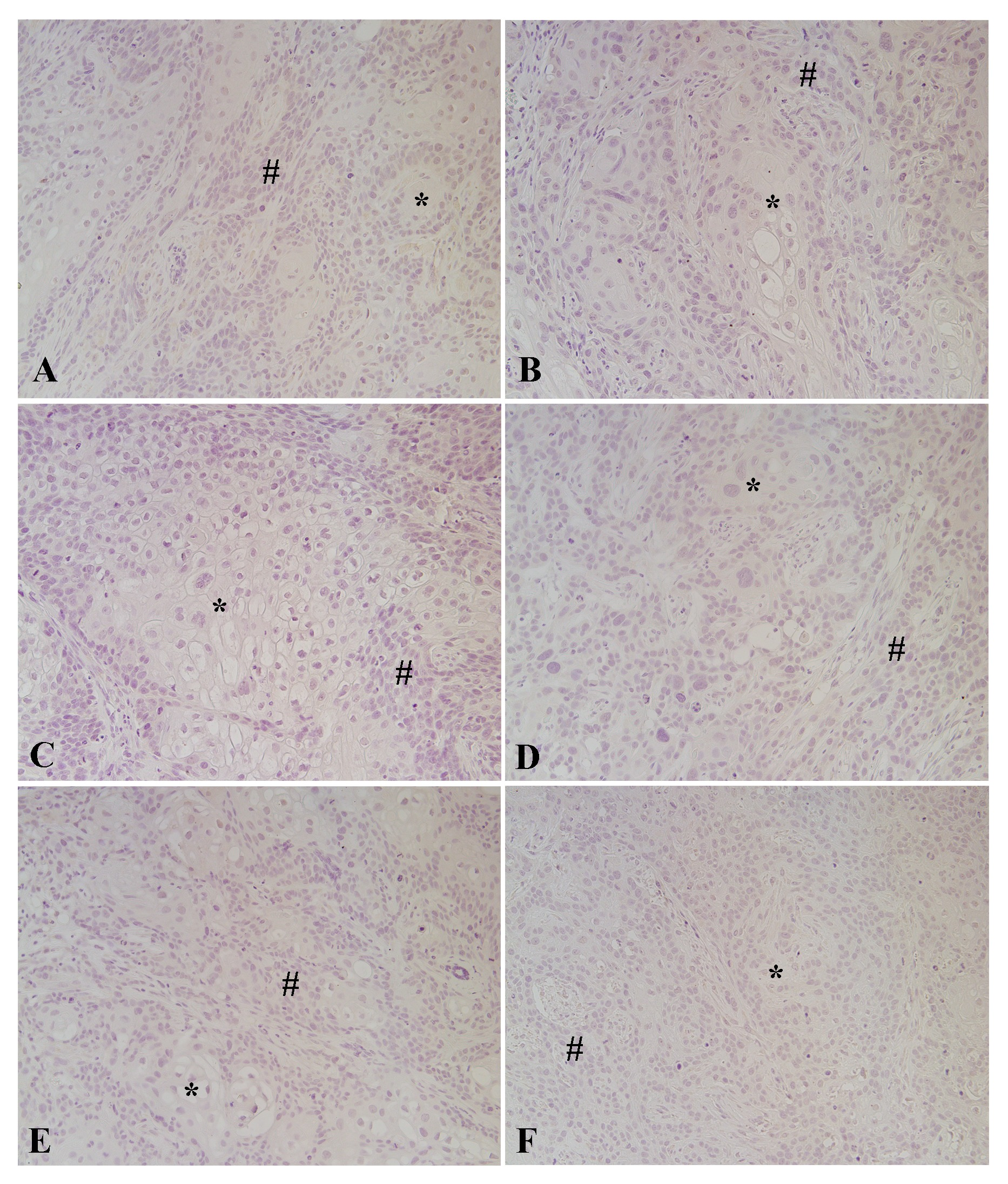

Supplement: S1 Fig — (A-F). Staining for As#1, As#2, As#3, As#4, As#5 and As#6 respectively. There is no staining for CDH3 in the well-differentiated cells (*) in the center of tumor nests as well as the peripheral less differentiated cells (#). All images are at a magnification of 200X. (TIF) [file pone.0207877.s001.tif]

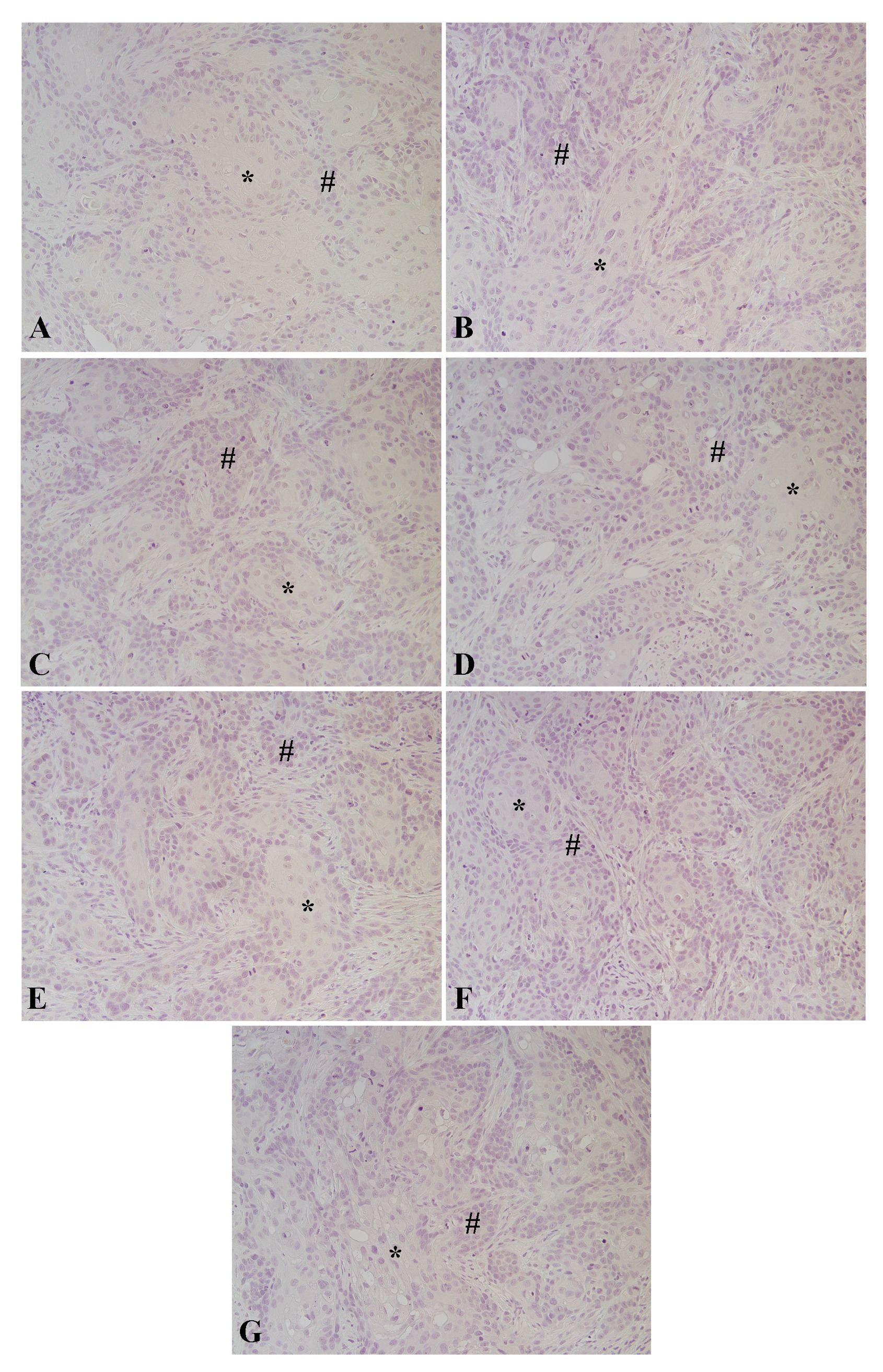

Supplement: S2 Fig — (A-G). Staining for Cd#1, Cd#2, Cd#3, Cd#4, Cd#5, Cd#6 and Cd#7 respectively. There is no staining for CDH3 in the well-differentiated cells (*) in the center of tumor nests as well as the peripheral less differentiated cells (#). All images are at a magnification of 200X. (TIF) [file pone.0207877.s002.tif]

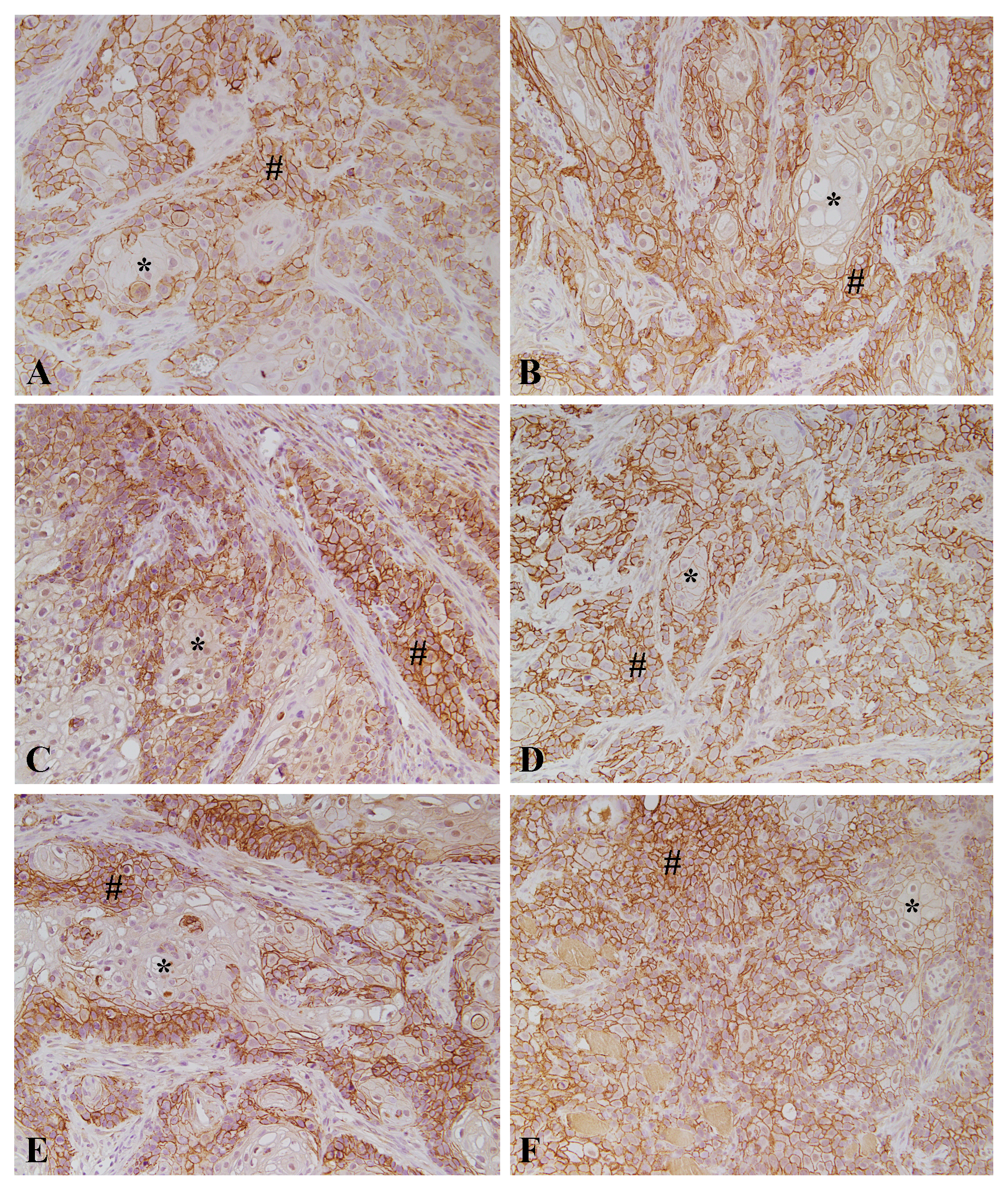

Supplement: S3 Fig — (A-F). Staining for As#1, As#2, As#3, As#4, As#5 and As#6 respectively. There is strong membranous staining for CD44 in the less differentiated cells located at the periphery of the tumor nests (#), whereas the well differentiated cells located in the center of the tumor nests (*) show weak or no staining for CD44. All images are at a magnification of 200X. (TIF) [file pone.0207877.s003.tif]

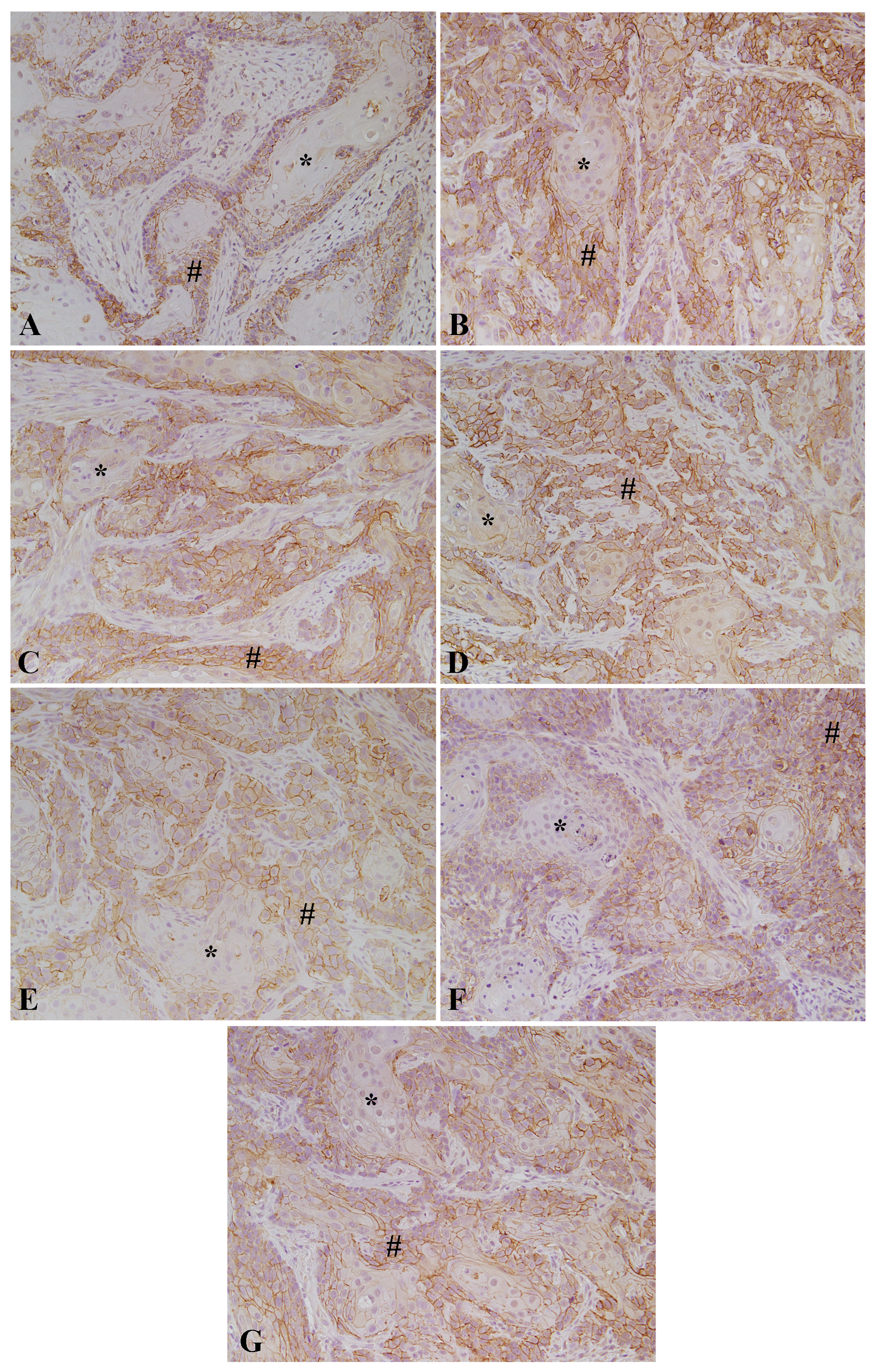

Supplement: S4 Fig — (A-G). Staining for Cd#1, Cd#2, Cd#3, Cd#4, Cd#5, Cd#6 and Cd#7 respectively. There is moderate to strong membranous staining for CD44 in the less differentiated cells located at the periphery of the tumor nests (#), whereas the well differentiated cells located in the center of the tumor nests show weak or no staining for CD44. All images are at a magnification of 200X. (TIF) [file pone.0207877.s004.tif]

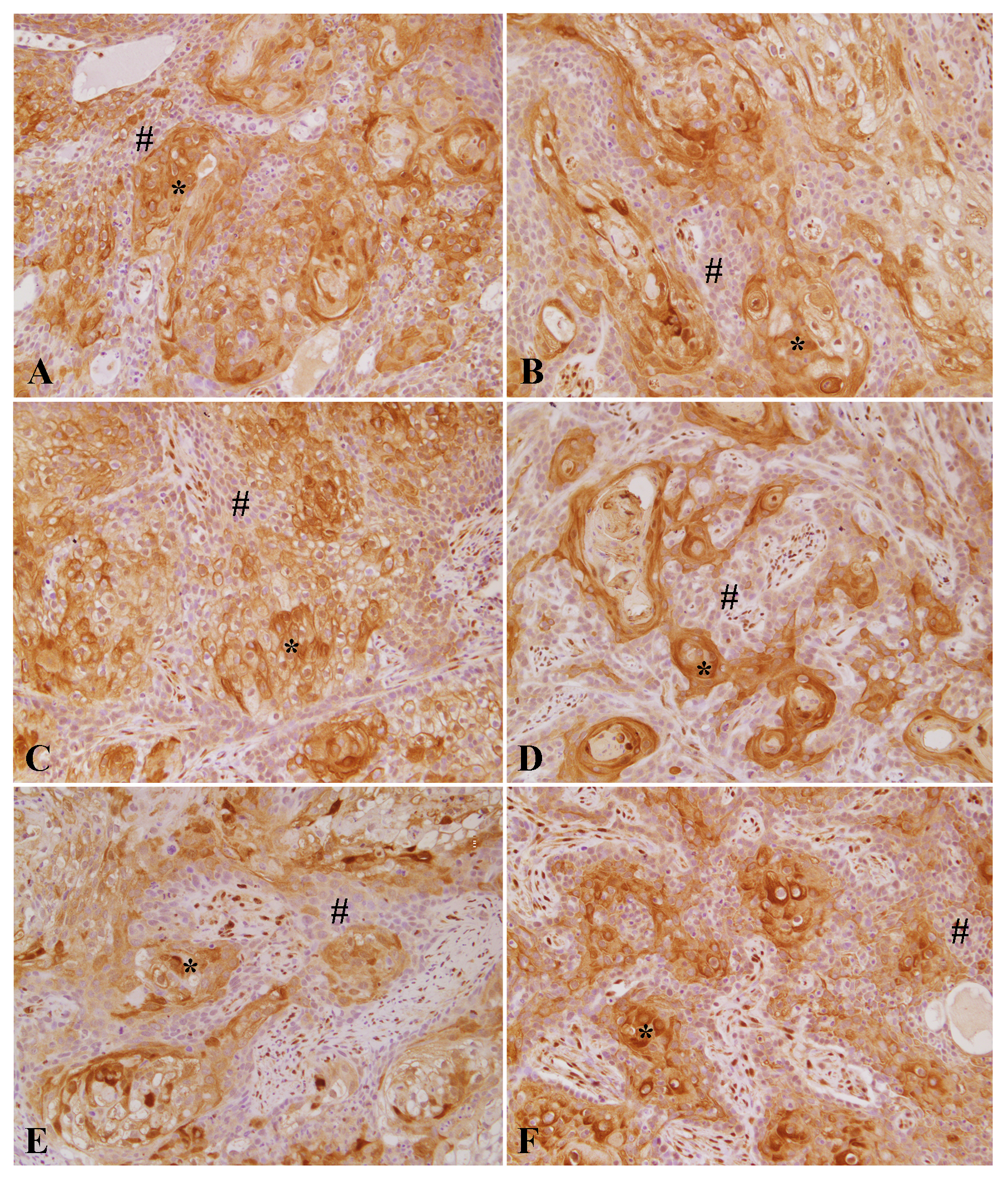

Supplement: S5 Fig — (A-F). Staining for As#1, As#2, As#3, As#4, As#5 and As#6 respectively. The well-differentiated cells (*) in the center of tumor nests are strongly positive for CK1, whereas the peripheral less differentiated cells (#) show weaker staining of CK1. All images are at a magnification of 200X. (TIF) [file pone.0207877.s005.tif]

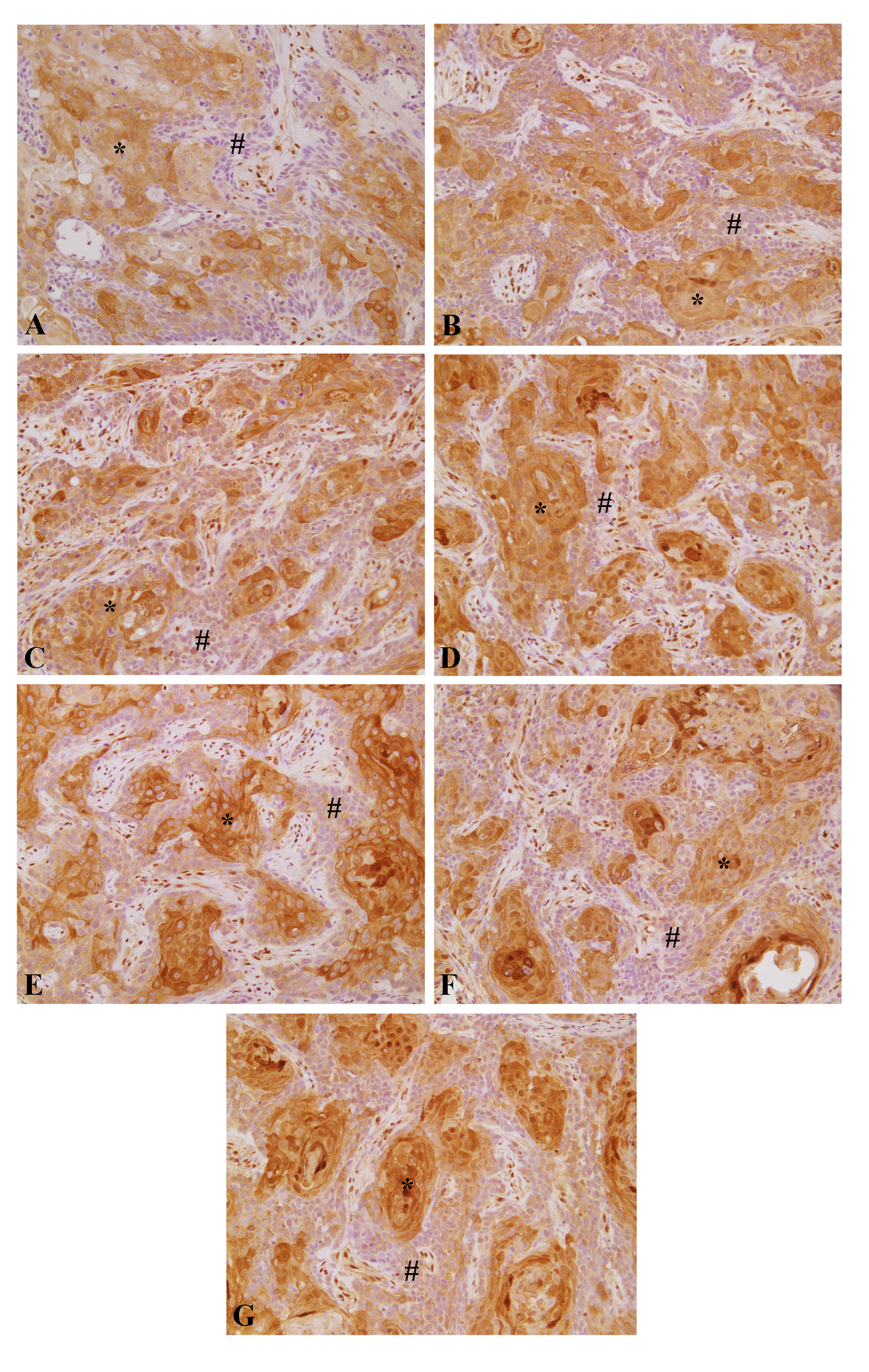

Supplement: S6 Fig — (A-G). Staining for Cd#1, Cd#2, Cd#3, Cd#4, Cd#5, CD#6 and Cd#7 respectively. The well- differentiated cells (*) in the center of tumor nests are strongly positive for CK1, whereas the peripheral less differentiated cells (#) show weaker staining of CK1. All images are at a magnification of 200X. (TIF) [file pone.0207877.s006.tif]

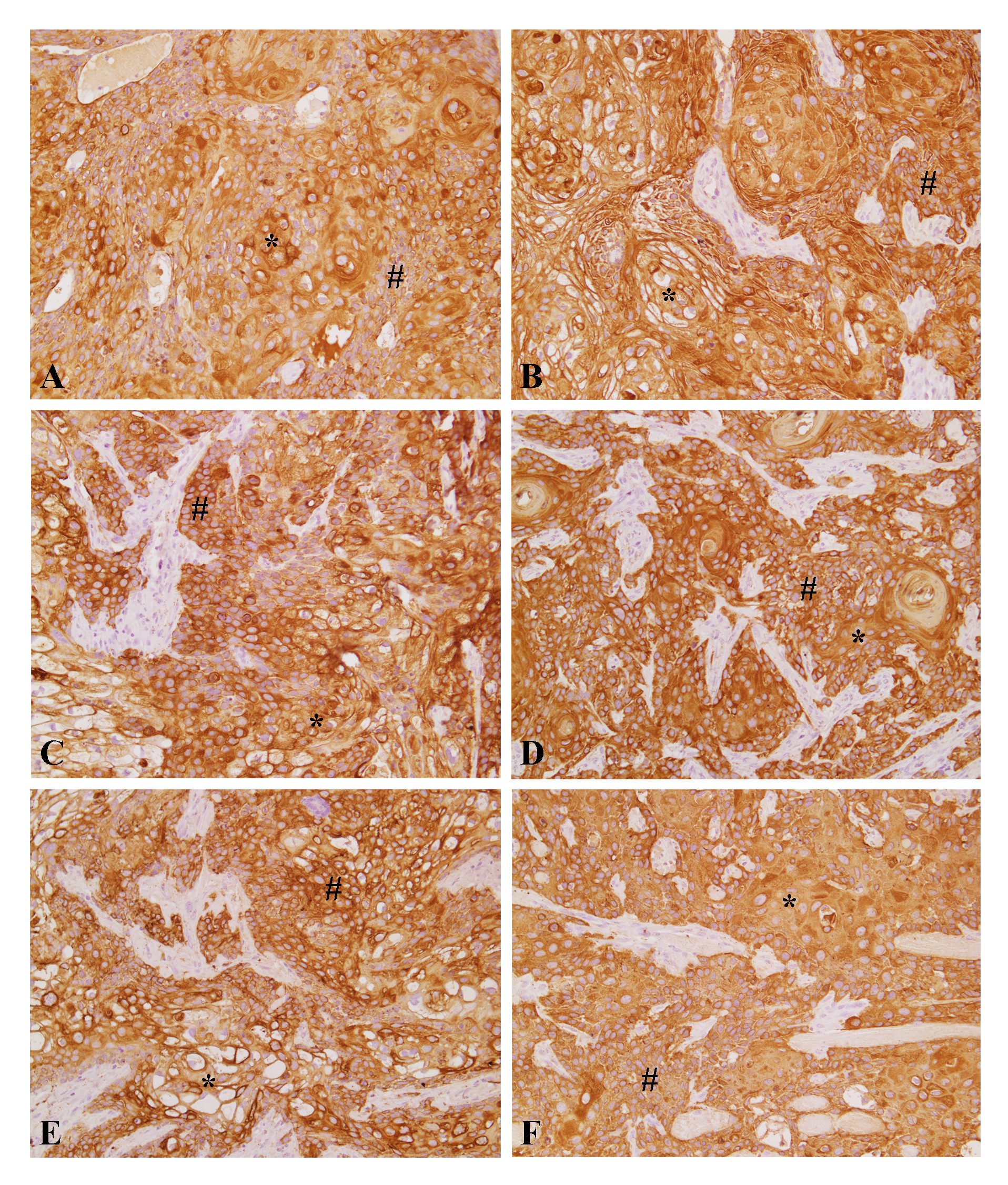

Supplement: S7 Fig — (A-F). Staining for As#1, As#2, As#3, As#4, As#5 and As#6 respectively. The staining for KRT5 is diffuse with strong staining in the differentiated (*) as well as less differentiated (#) areas of the tumor. All images are at a magnification of 200X. (TIF) [file pone.0207877.s007.tif]

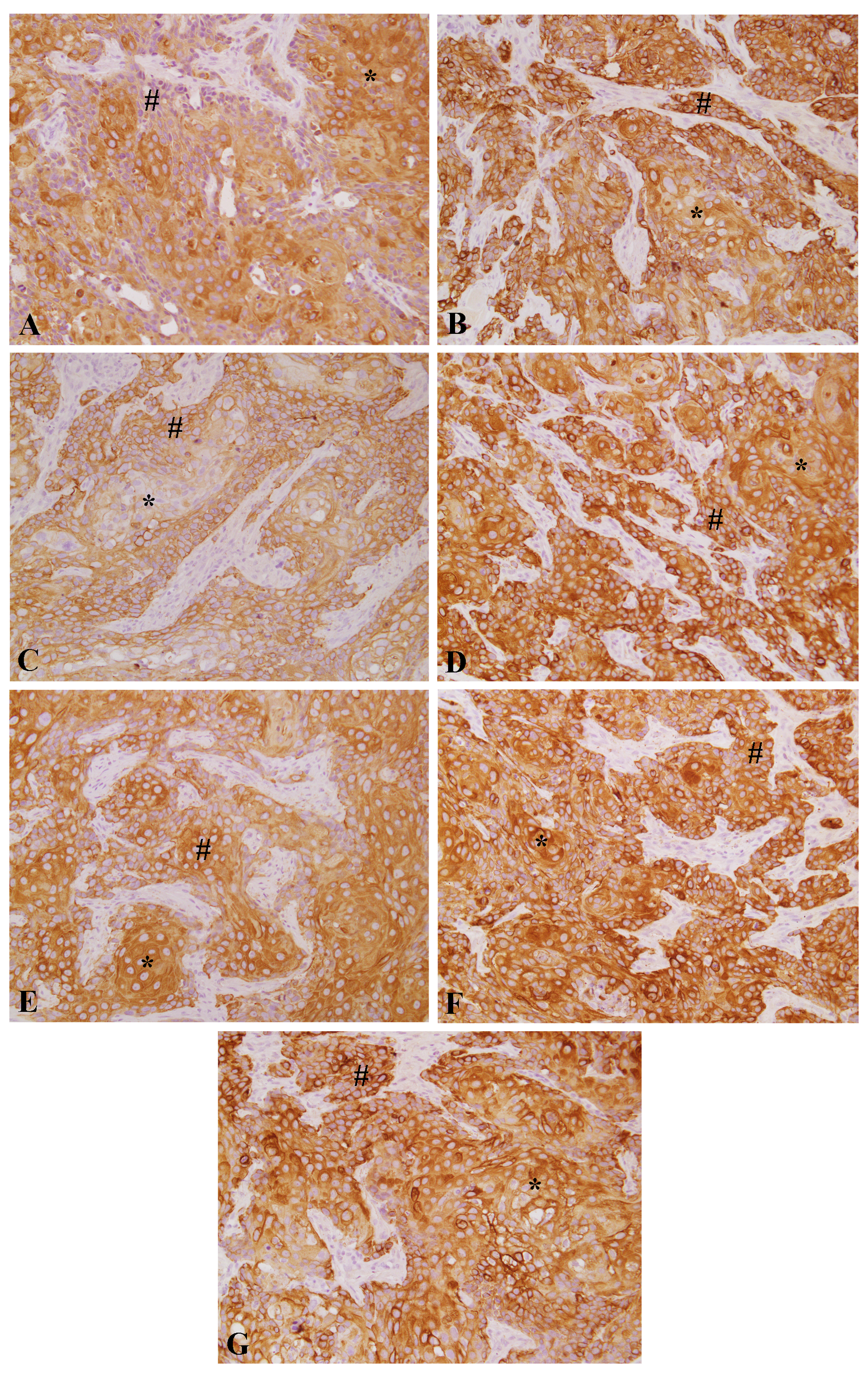

Supplement: S8 Fig — (A-G). Staining for Cd#1, Cd#2, Cd#3, Cd#4, Cd#5, Cd#6 and Cd#7 respectively. The staining for KRT5 is diffuse with strong staining in the differentiated (*) as well as less differentiated (#) areas of the tumor. All images are at a magnification of 200X. (TIF) [file pone.0207877.s008.tif]

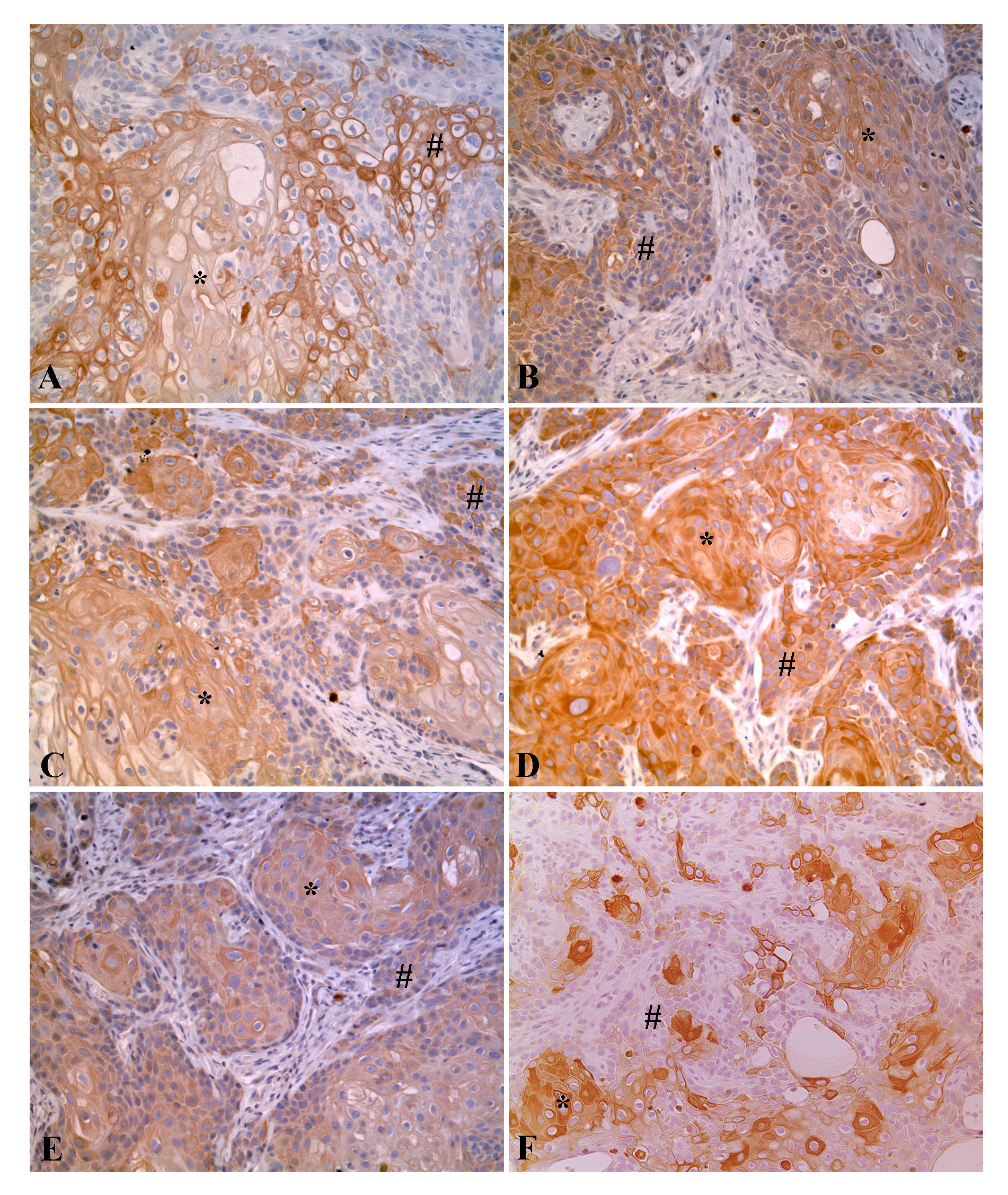

Supplement: S9 Fig — (A-F). Staining for As#1, As#2, As#3, As#4, As#5 and As#6 respectively. The staining for KRT6 is strong in the well-differentiated (*) cells located in the center of the tumor nests with squamous features, whereas the staining is weak to absent in the less differentiated cells (#) located at the periphery of the tumor nests. All images are at a magnification of 200X. (TIF) [file pone.0207877.s009.tif]

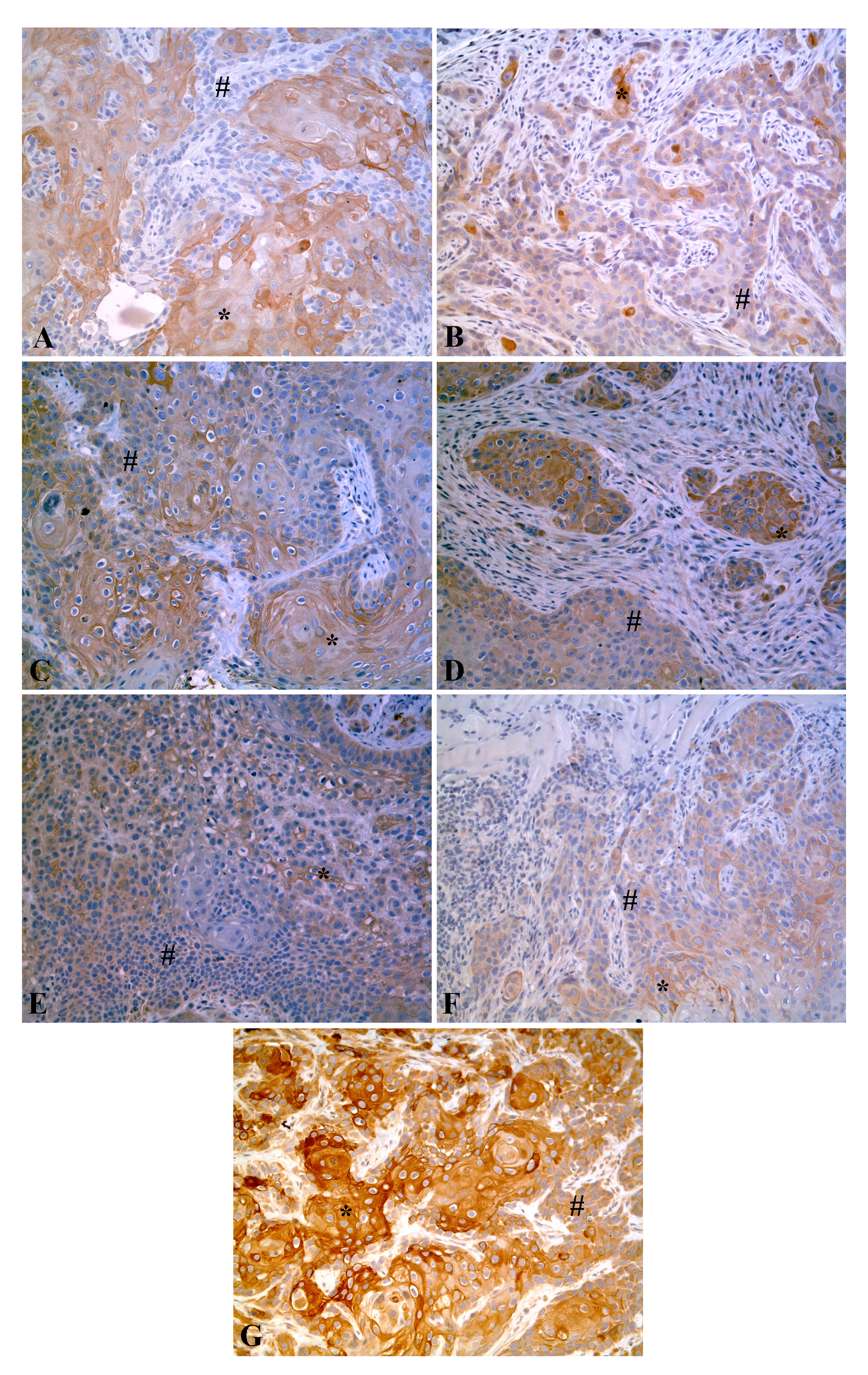

Supplement: S10 Fig — (A-G). Staining for Cd#1, Cd#2, Cd#3, Cd#4, Cd#5, Cd#6 and Cd#7 respectively The staining for KRT6 is strong in the well-differentiated cells located in the center of the tumor nests with squamous features, whereas the staining is weak to absent in the less differentiated cells (#) located at the periphery of the tumor nests. All images are at a magnification of 200X. (TIF) [file pone.0207877.s010.tif]

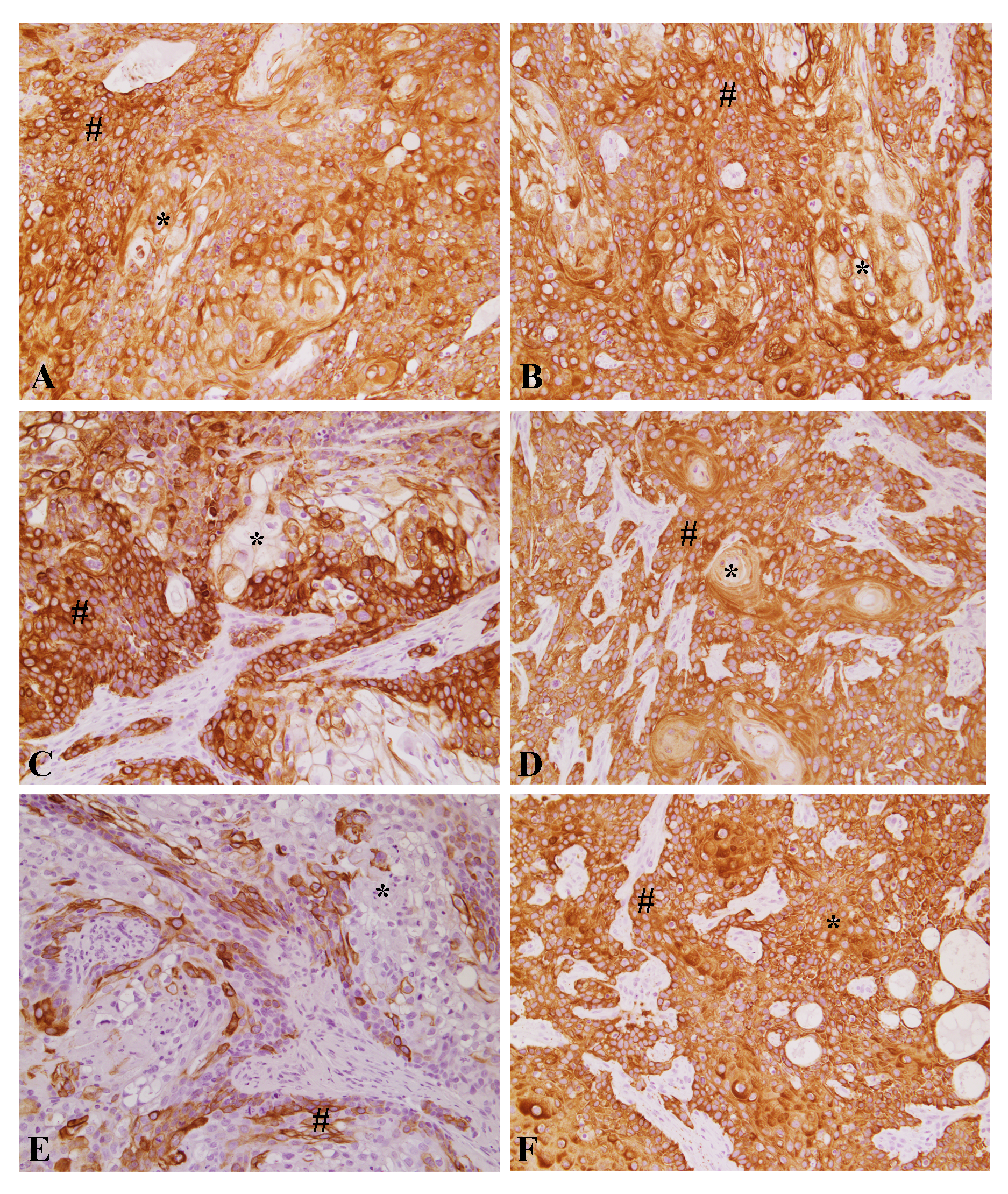

Supplement: S11 Fig — (A-F). Staining for As#1, As#2, As#3, As#4, As#5 and As#6 respectively. For As#1, As#2, As#4, and As#6, the staining for KRT14 is diffuse with strong staining in the differentiated (*) as well as less differentiated (#) area of the tumor. For As#3 and As#5, the well differentiated cells in the center of the tumor nests (*) show weak or no staining for KRT14, whereas the less differentiated peripheral cells (#) are strongly positive for KRT14. All images are at a magnification of 200X. (TIF) [file pone.0207877.s011.tif]

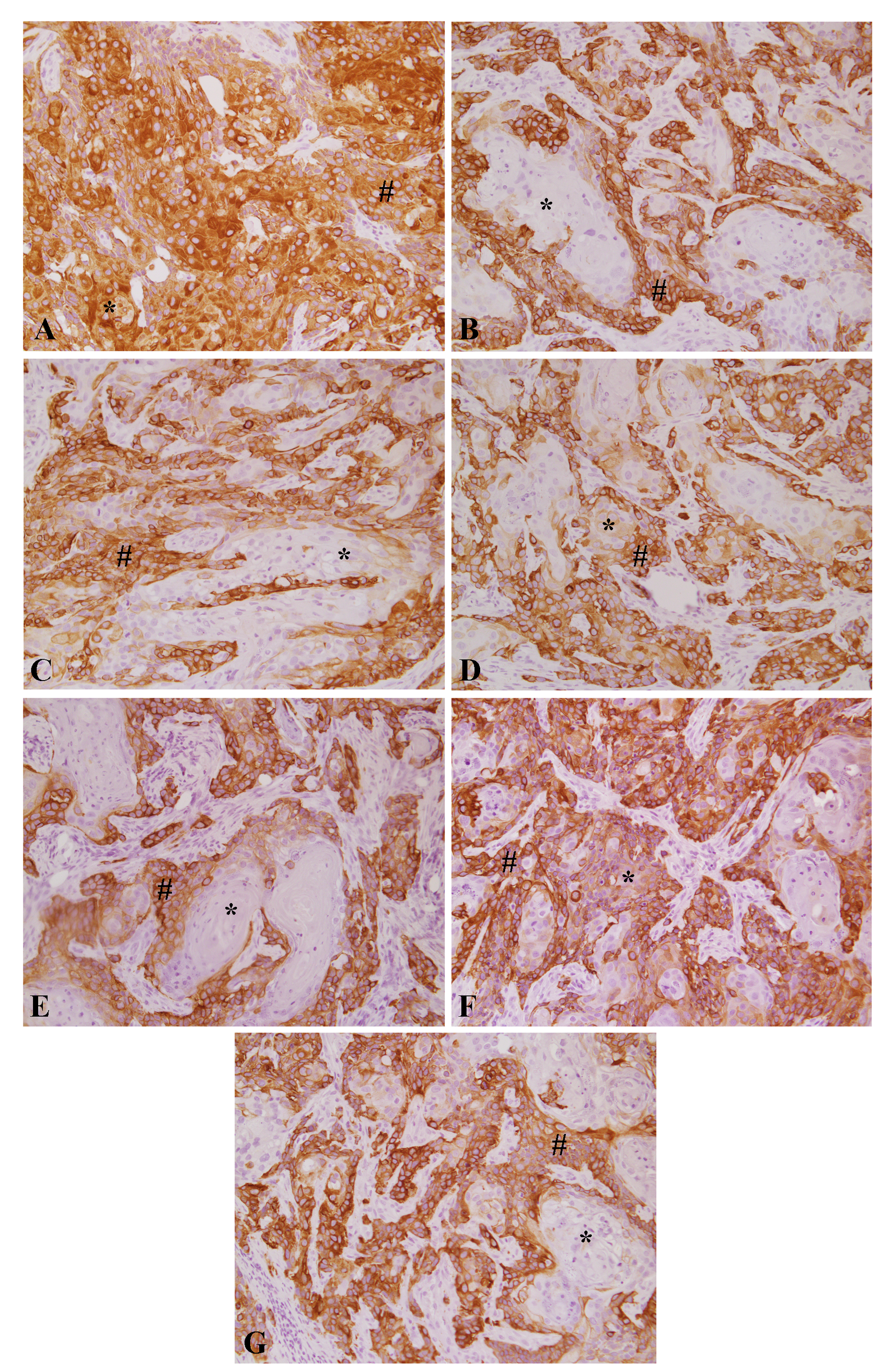

Supplement: S12 Fig — (A-G). Staining for Cd#1, Cd#2, Cd#3, Cd#4, Cd#5, Cd#6 and Cd#7 respectively. For Cd#1, the staining for KRT14 is diffuse with strong staining in the differentiated (*) as well as less differentiated (#) area of the tumor. For Cd#2, Cd#3, Cd#4, Cd#5, Cd#6 and Cd#7, the well-differentiated cells (*) in the center of the tumor nests show weak or no staining for KRT14, whereas the less differentiated peripheral cells (#) show strong staining for KRT14. All images are at a magnification of 200X. (TIF) [file pone.0207877.s012.tif]

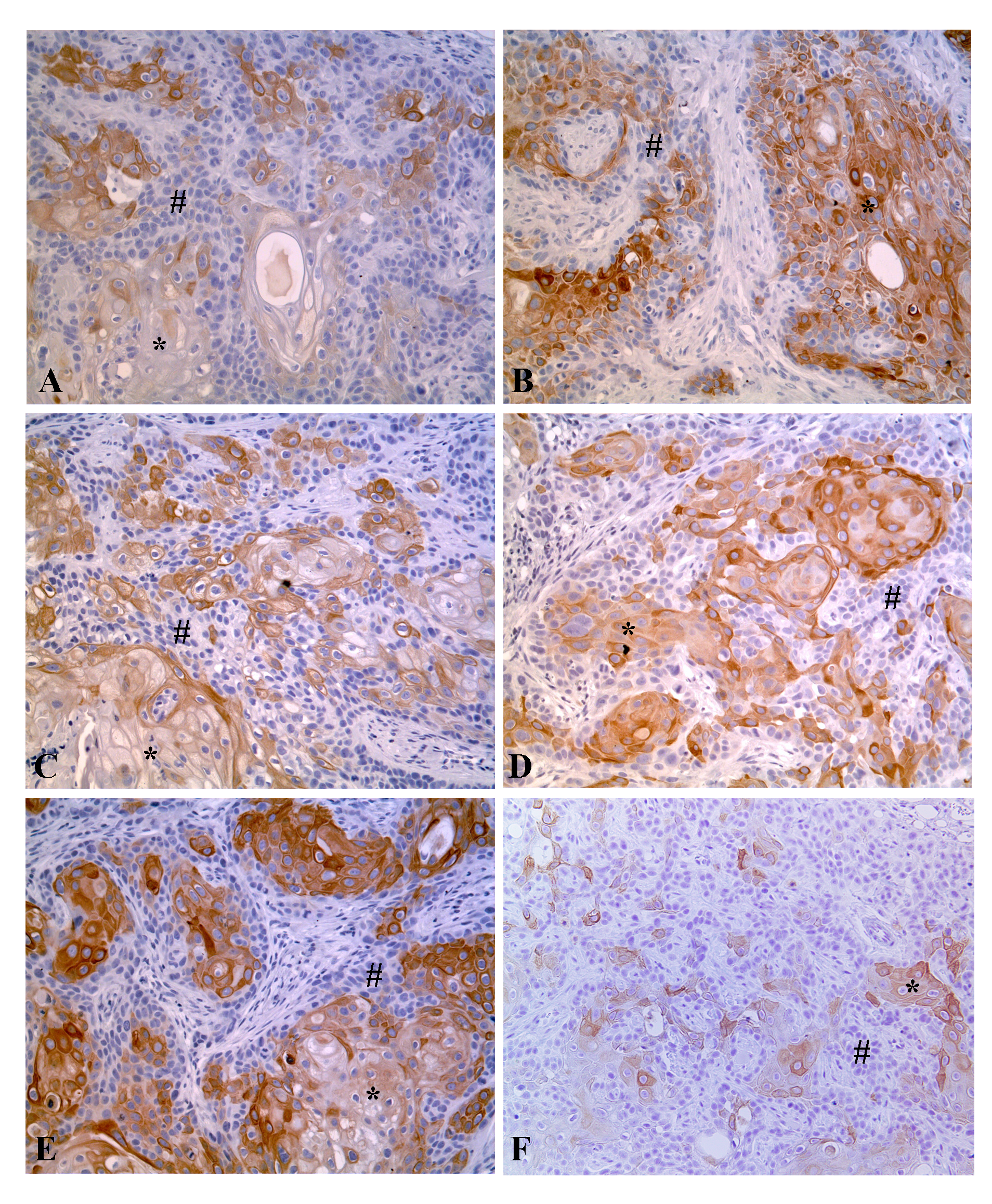

Supplement: S13 Fig — (A-F). Staining for As#1, As#2, As#3, As#4, As#5 and As#6 respectively. The staining for KRT16 is moderate to strong in the well-differentiated cells (*) located in the center of the tumor nests with squamous features, whereas the staining is weak to absent in the less differentiated cells (#) located at the periphery of the tumor nests. All images are at a magnification of 200X. (TIF) [file pone.0207877.s013.tif]

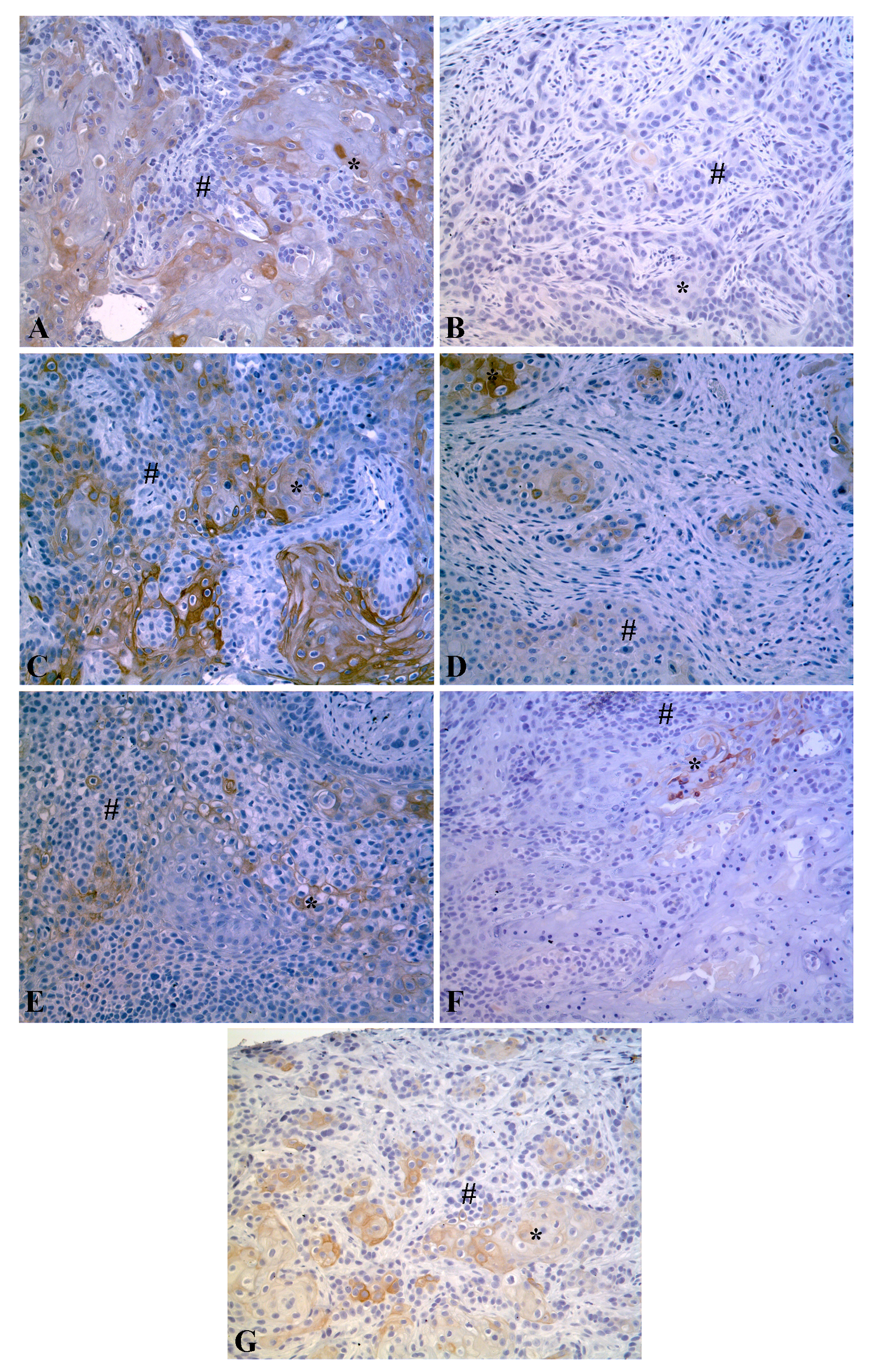

Supplement: S14 Fig — (A-G). Staining for Cd#1, Cd#2, Cd#3, Cd#4, Cd#5, Cd#6 and Cd#7 respectively. The staining for KRT16 is strong in Cd#1, whereas in Cd#3, Cd#4, Cd#5, Cd#6 and Cd#7, the staining for KRT16 is moderate. In Cd#2, the staining is absent for KRT16. The staining is in the well-differentiated cells (*) located in the center of the tumor nests with squamous features, whereas the staining is absent in the less differentiated cells (#) located at the periphery of the tumor nests. All images are at a magnification of 200X. (TIF) [file pone.0207877.s014.tif]

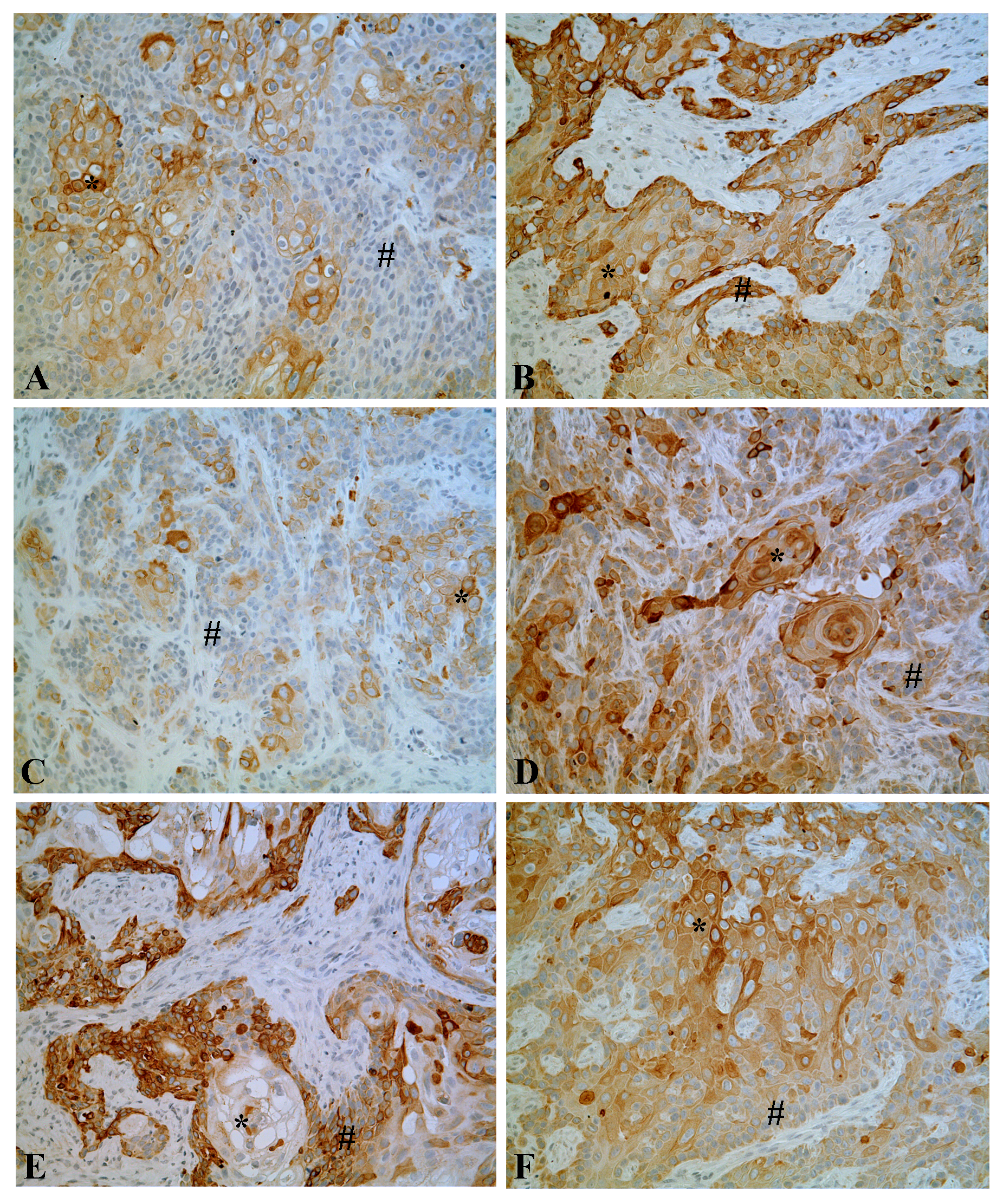

Supplement: S15 Fig — (A-F). Staining for As#1, As#2, As#3, As#4, As#5 and As#6 respectively. The staining for KRT17 is strong in the well-differentiated cells (*) located in the center of the tumor nests with squamous features. In tumors formed by As#2 (B) and As#5(E), there is also strong staining in the less differentiated basal-like cells (#) located at the periphery of the tumor nests. All images are at a magnification of 200X. (TIF) [file pone.0207877.s015.tif]

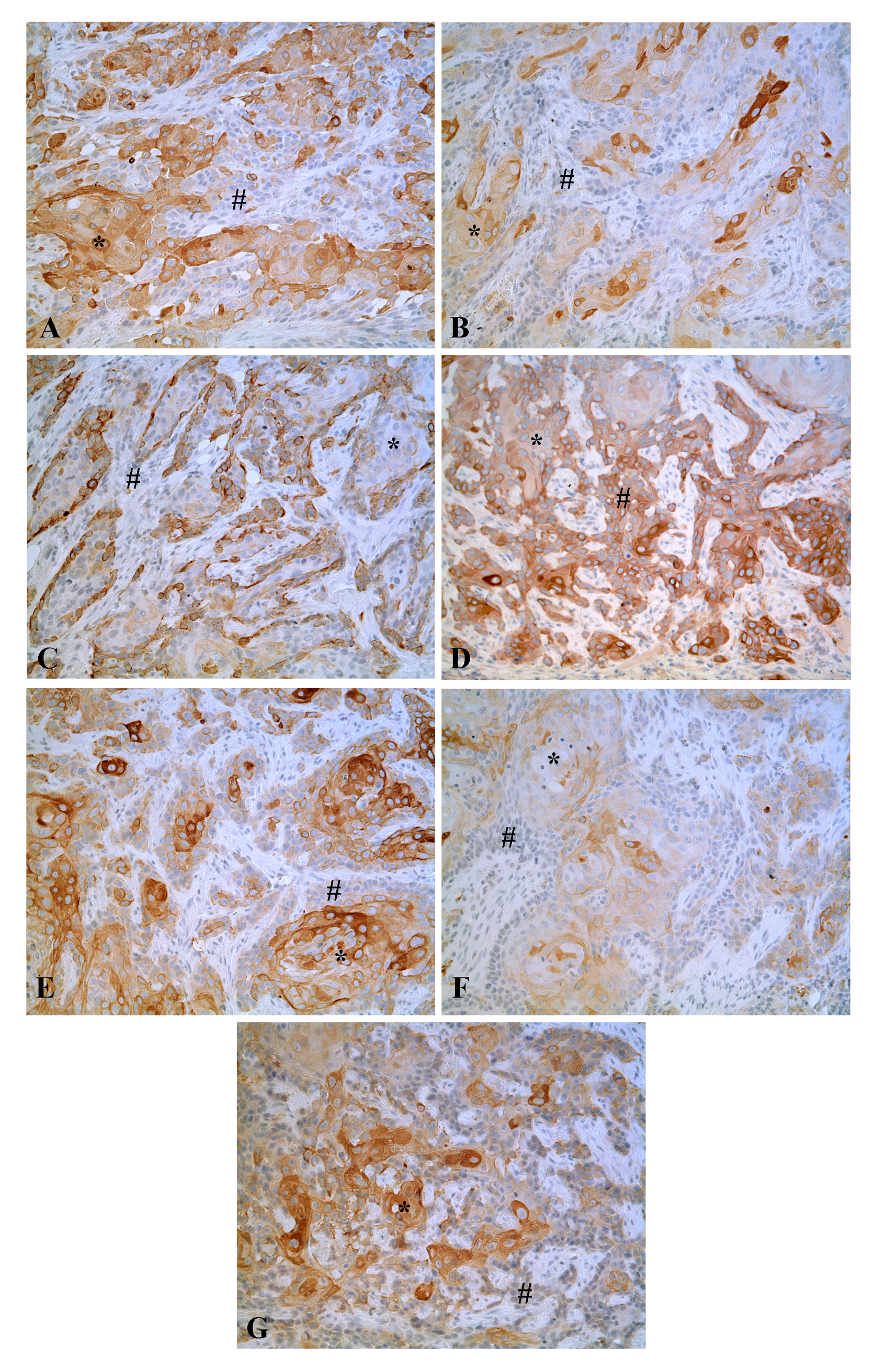

Supplement: S16 Fig — (A-G). Staining for Cd#1, Cd#2, Cd#3, Cd#4, Cd#5, Cd#6 and Cd#7 respectively. The staining for KRT17 is strong in the well-differentiated cells (*) located in the center of the tumor nests with squamous features. In tumors formed by Cd#3 (C) and Cd#4 (D), there is also strong staining in the less differentiated basal-like cells (#) located at the periphery of the tumor nests. All images are at a magnification of 200X. (TIF) [file pone.0207877.s016.tif]

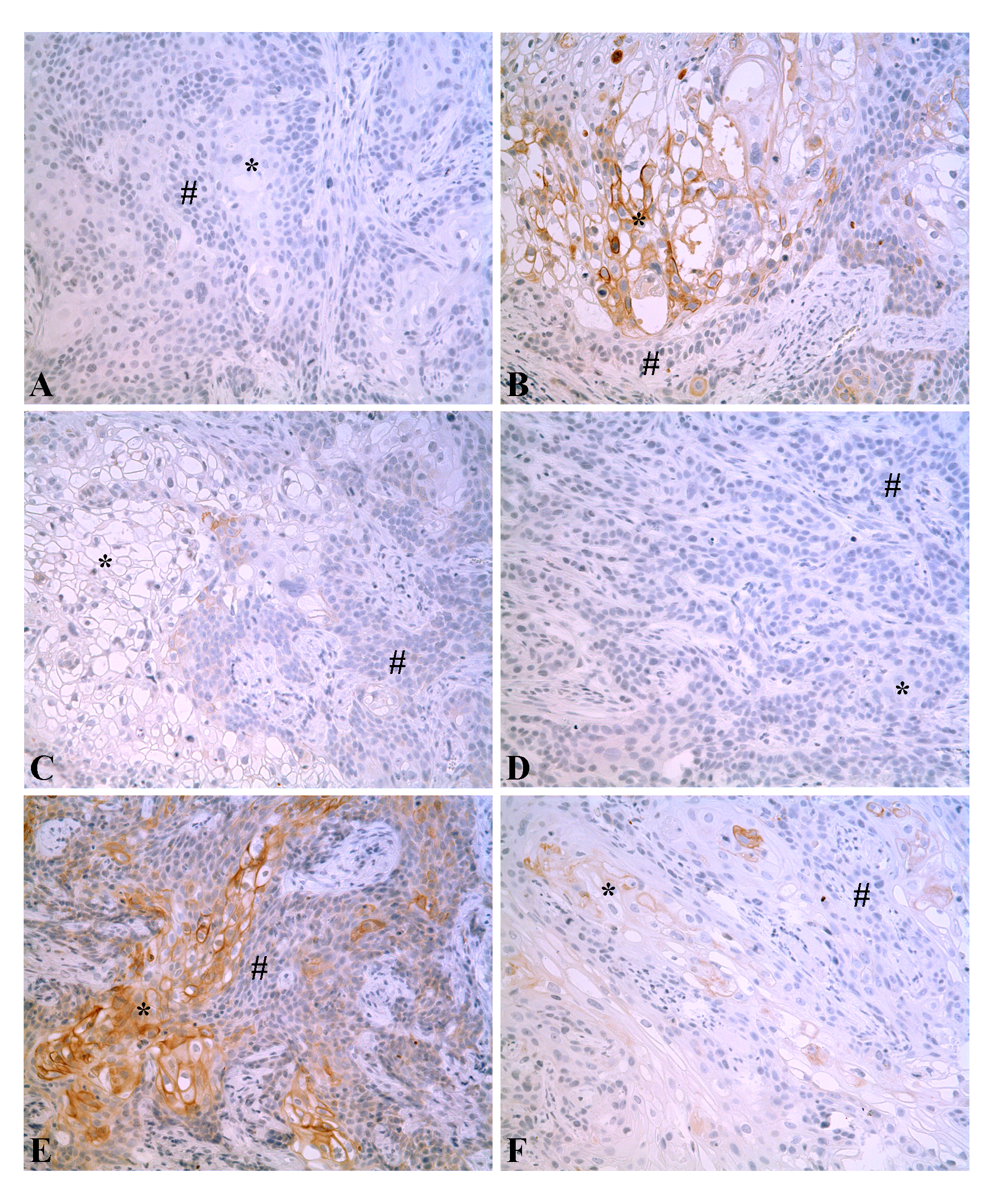

Supplement: S17 Fig — (A-F). Staining for As#1, As#2, As#3, As#4, As#5 and As#6 respectively. There is no staining in the tumors for As#1 and As#4 (A and D). For As#2 (B), As#3 (C) and As#6 (F), the expression is focal, whereas for As#5 (E), the staining is strong and diffuse. In all the positively stained sections, the staining was localized to the well-differentiated cells (*) in the center of the tumor nests. There is no staining in the less differentiated basal-like cells (#) located at the periphery of the tumor nests. All images are at a magnification of 200X. (TIF) [file pone.0207877.s017.tif]

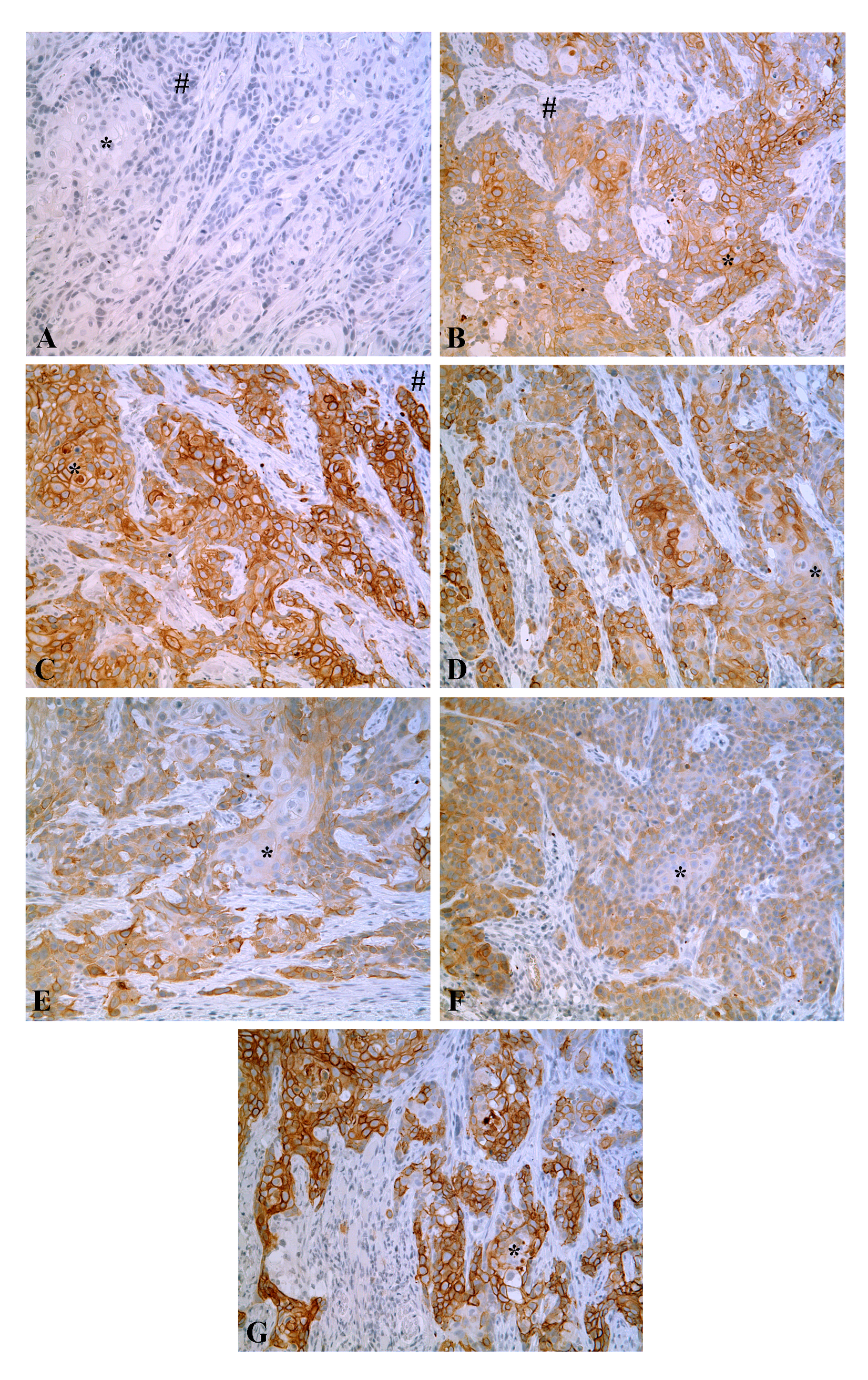

Supplement: S18 Fig — (A-G). Staining for Cd#1, Cd#2, Cd#3, Cd#4, Cd#5, Cd#6 and Cd#7 respectively. There was no staining in the tumor for Cd#1(A). For Cd#2, Cd#3, Cd#4, Cd#5, Cd#6 and Cd#7 (B-G), there is strong staining that is diffuse and localized to the well differentiated cells in the center of the tumor nests (*). There is no staining in the less differentiated basal-like cells (#) located at the periphery of the tumor nests. All images are at a magnification of 200X. (TIF) [file pone.0207877.s018.tif]

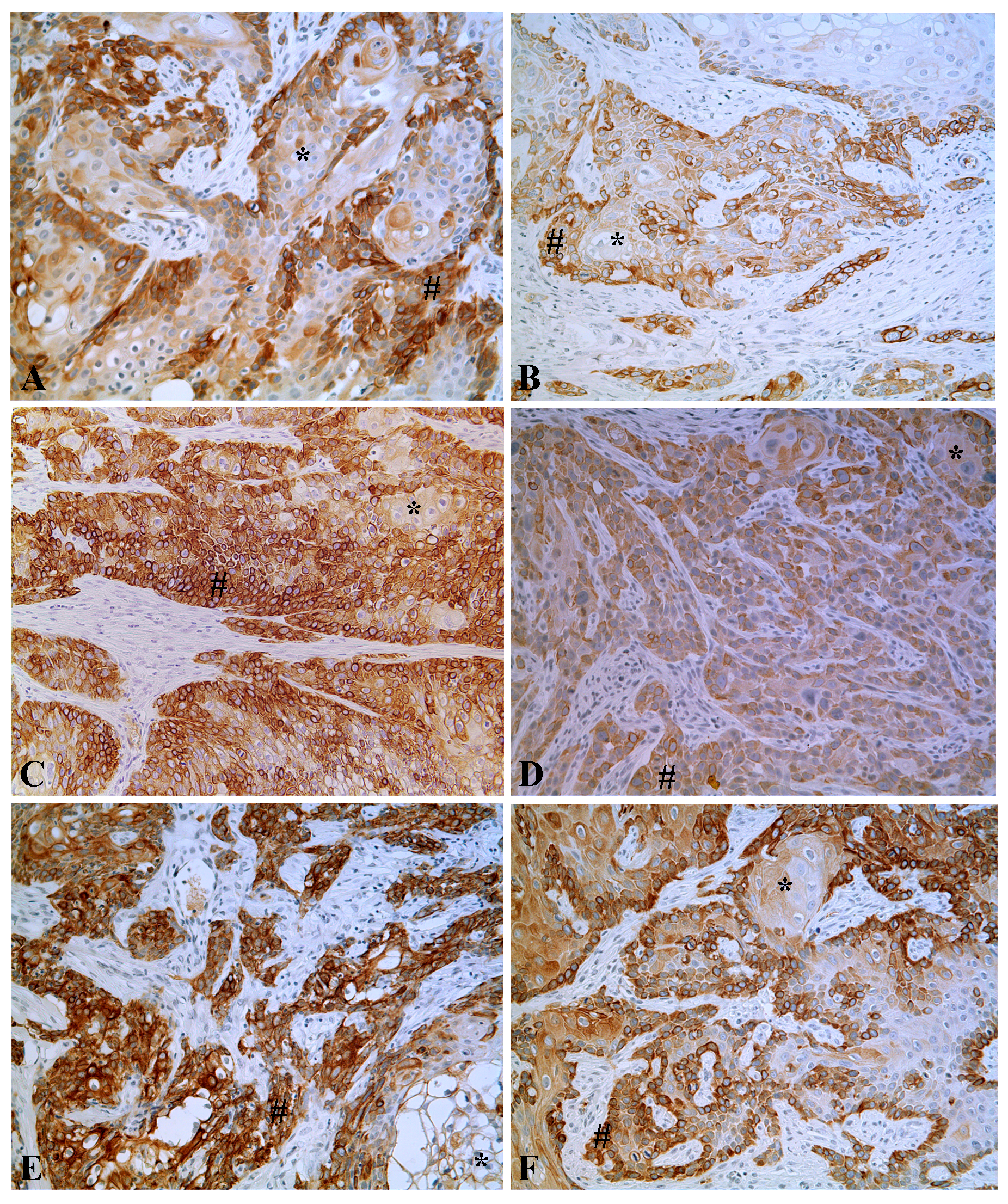

Supplement: S19 Fig — (A-F). Staining for As#1, As#2, As#3, As#4, As#5 and As#6 respectively. The expression of KRT19 is strong in all the tumor transplants with staining mainly in the less differentiated cells (#) located at the periphery of the tumor nests whereas the well differentiated cells (#) area either weakly positive or negative for the staining of KRT19. All images are at a magnification of 200X. (TIF) [file pone.0207877.s019.tif]

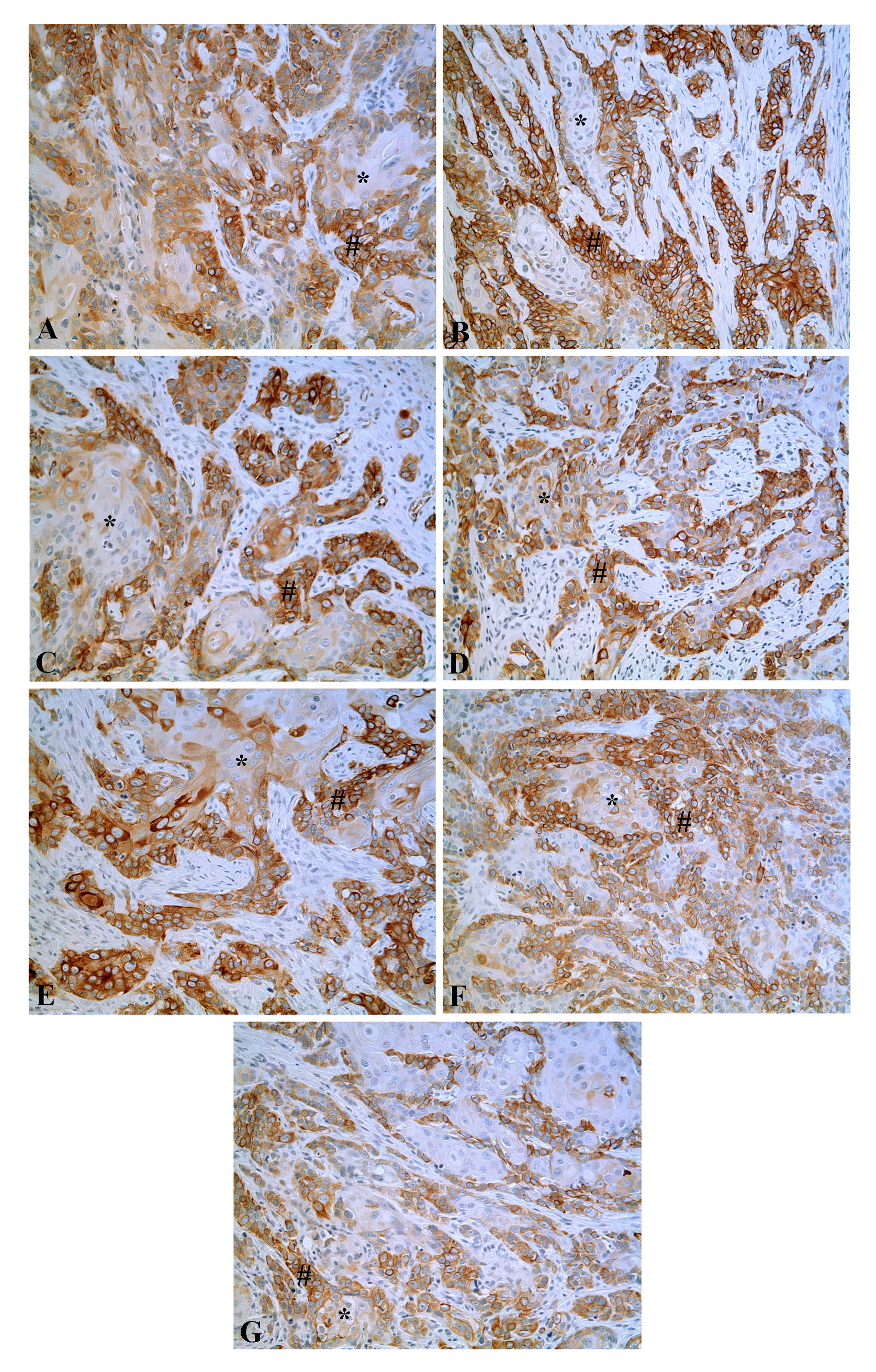

Supplement: S20 Fig — (A-G). Staining for Cd#1, Cd#2, Cd#3, Cd#4, Cd#5, Cd#6 and Cd#7 respectively. The expression of KRT19 is strong in all the tumor transplants with staining mainly in the less differentiated cells (#) located at the periphery of the tumor nests whereas the well differentiated cells (*) are either weakly positive or negative for the staining of KRT19. All images are at a magnification of 200X. (TIF) [file pone.0207877.s020.tif]

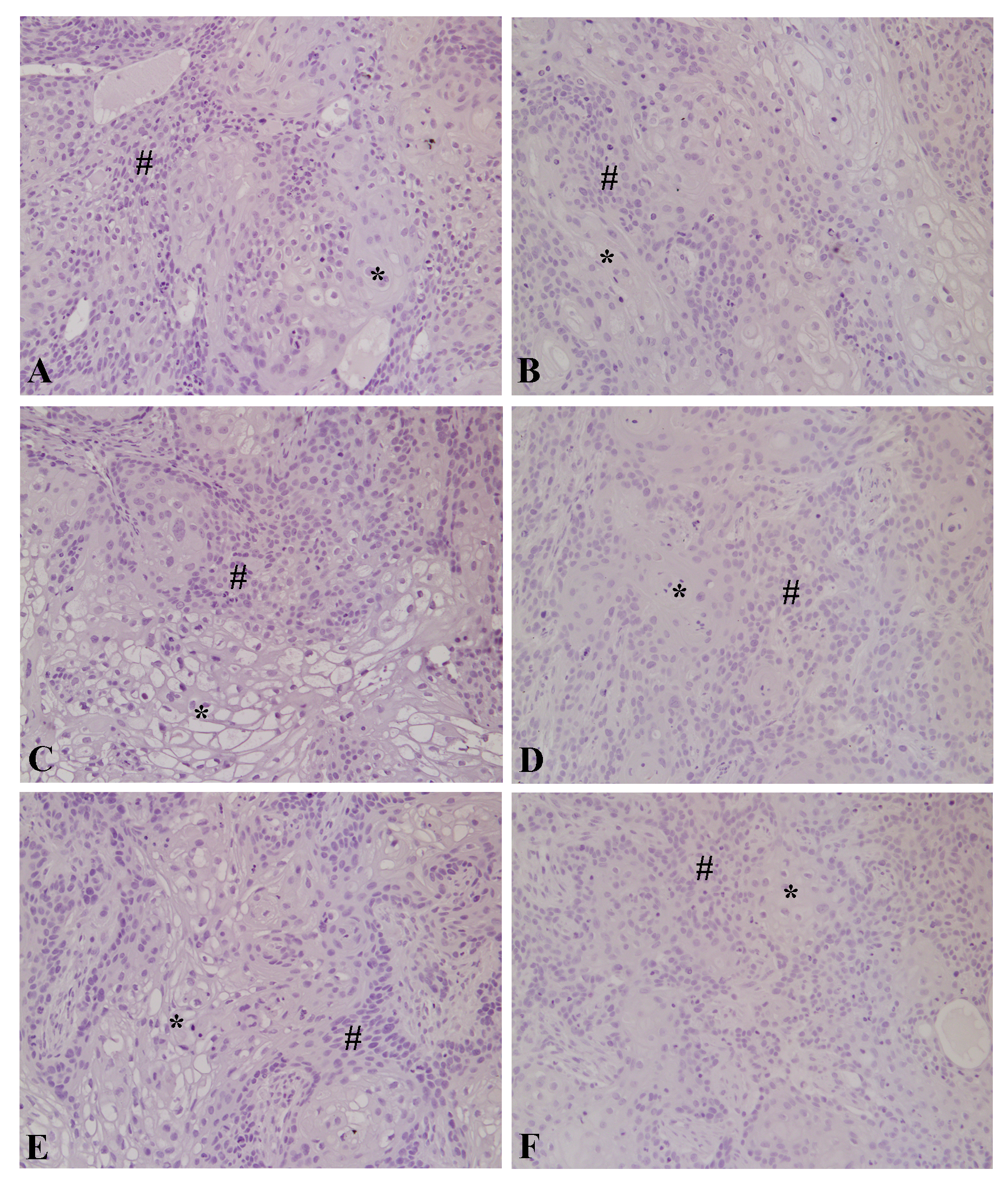

Supplement: S21 Fig — (A-F). Staining for As#1, As#2, As#3, As#4, As#5 and As#6 respectively. There is no staining for KRT20 in the tumor transplants. * indicates the well-differentiated areas whereas # indicates the less differentiated areas of the tumor. All images are at a magnification of 200X. (TIF) [file pone.0207877.s021.tif]

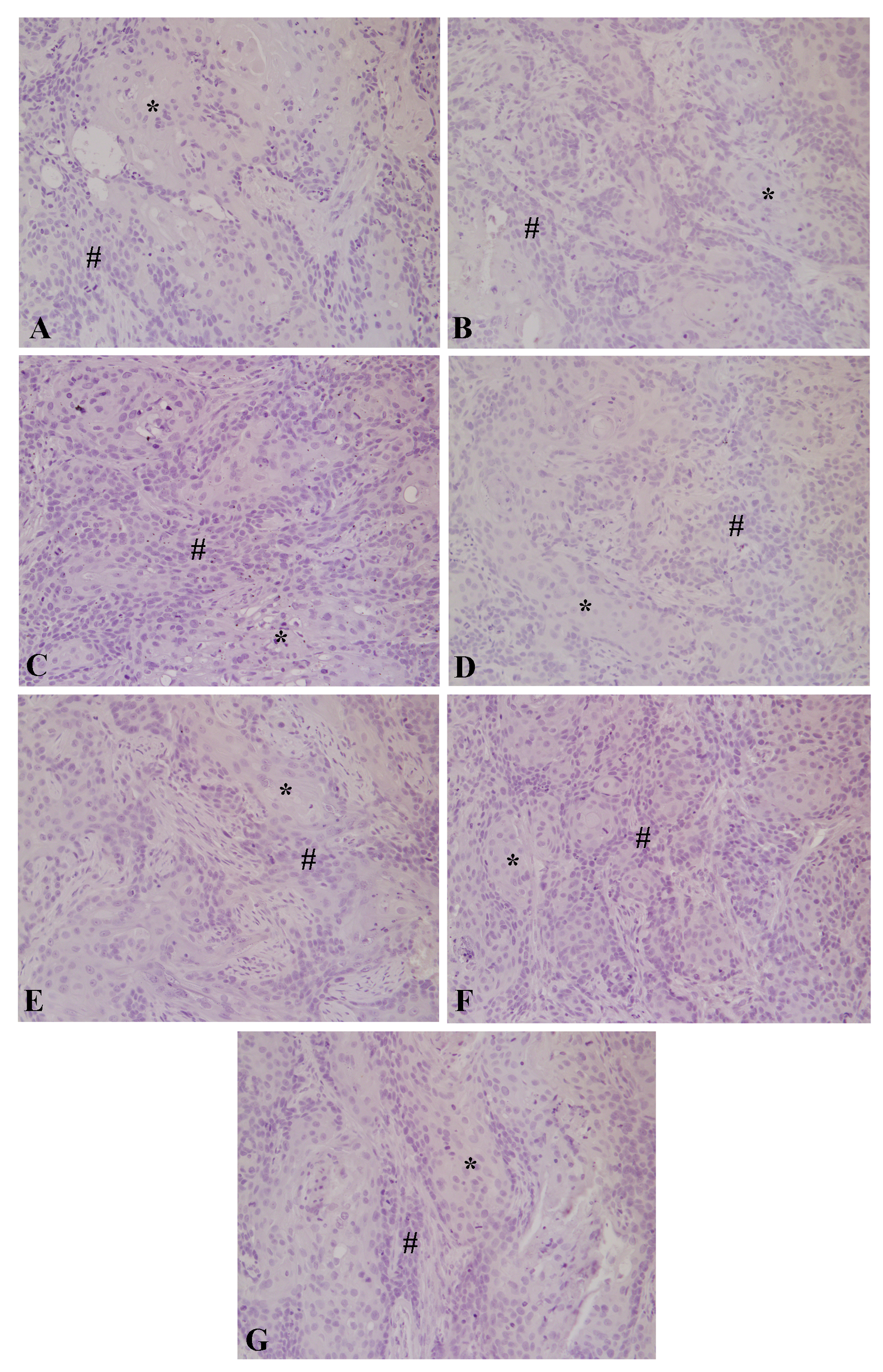

Supplement: S22 Fig — (A-G). Staining for Cd#1, Cd#2, Cd#3, Cd#4, Cd#5, Cd#6 and Cd#7 respectively. There is no staining for KRT20 in the tumor transplants. * indicates the well-differentiated areas whereas # indicates the less differentiated areas of the tumor. All images are at a magnification of 200X. (TIF) [file pone.0207877.s022.tif]

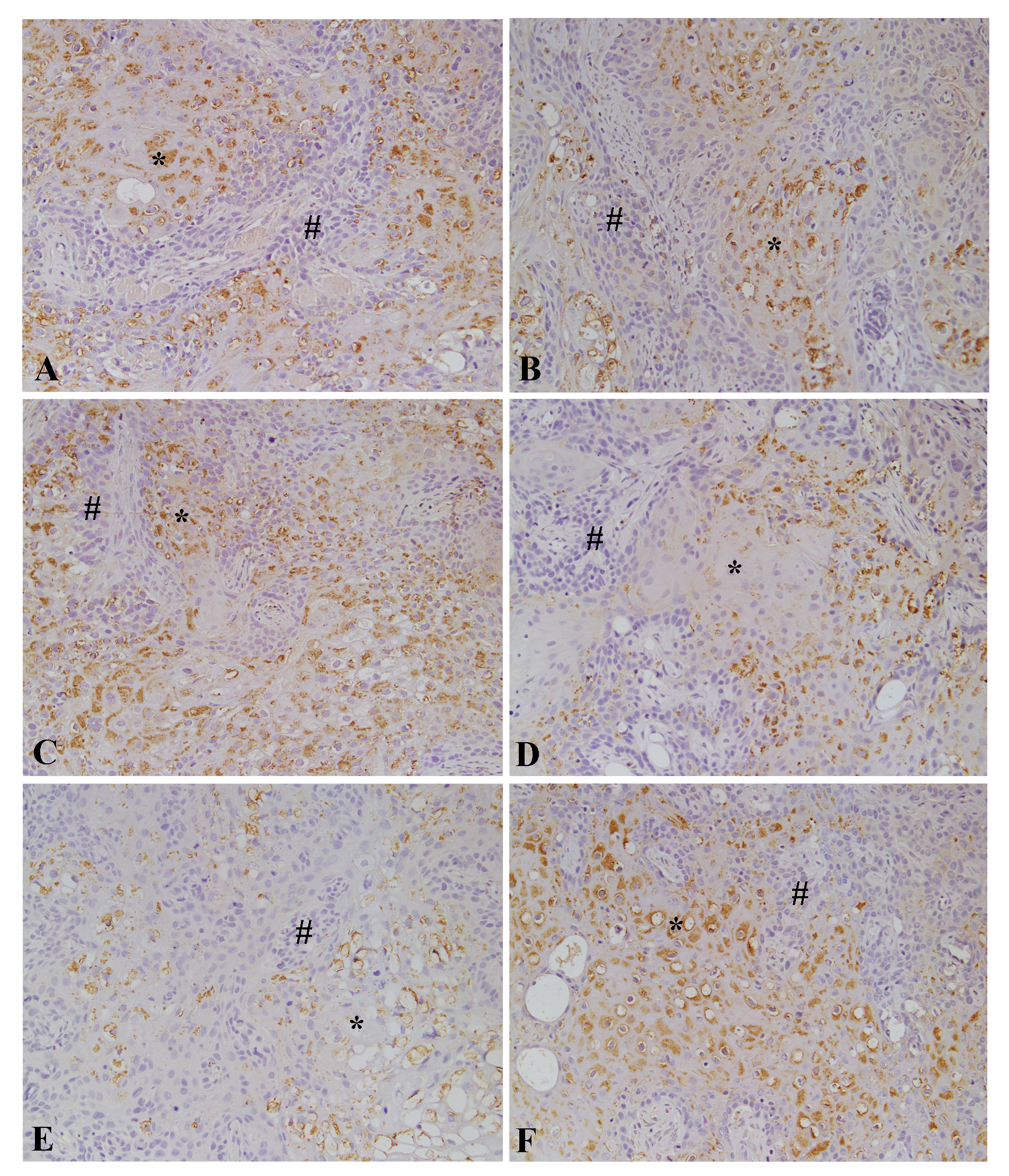

Supplement: S23 Fig — (A-F). Staining for As#1, As#2, As#3, As#4, As#5 and As#6 respectively. The staining for CD24 is granular with moderate to strong staining in the well-differentiated cells (*) located in the center of tumor nests, whereas the staining is absent in the less differentiated cells (#) located at the periphery of the tumor nests. The staining in As#4 is focal. All images are at a magnification of 200X. (TIF) [file pone.0207877.s023.tif]

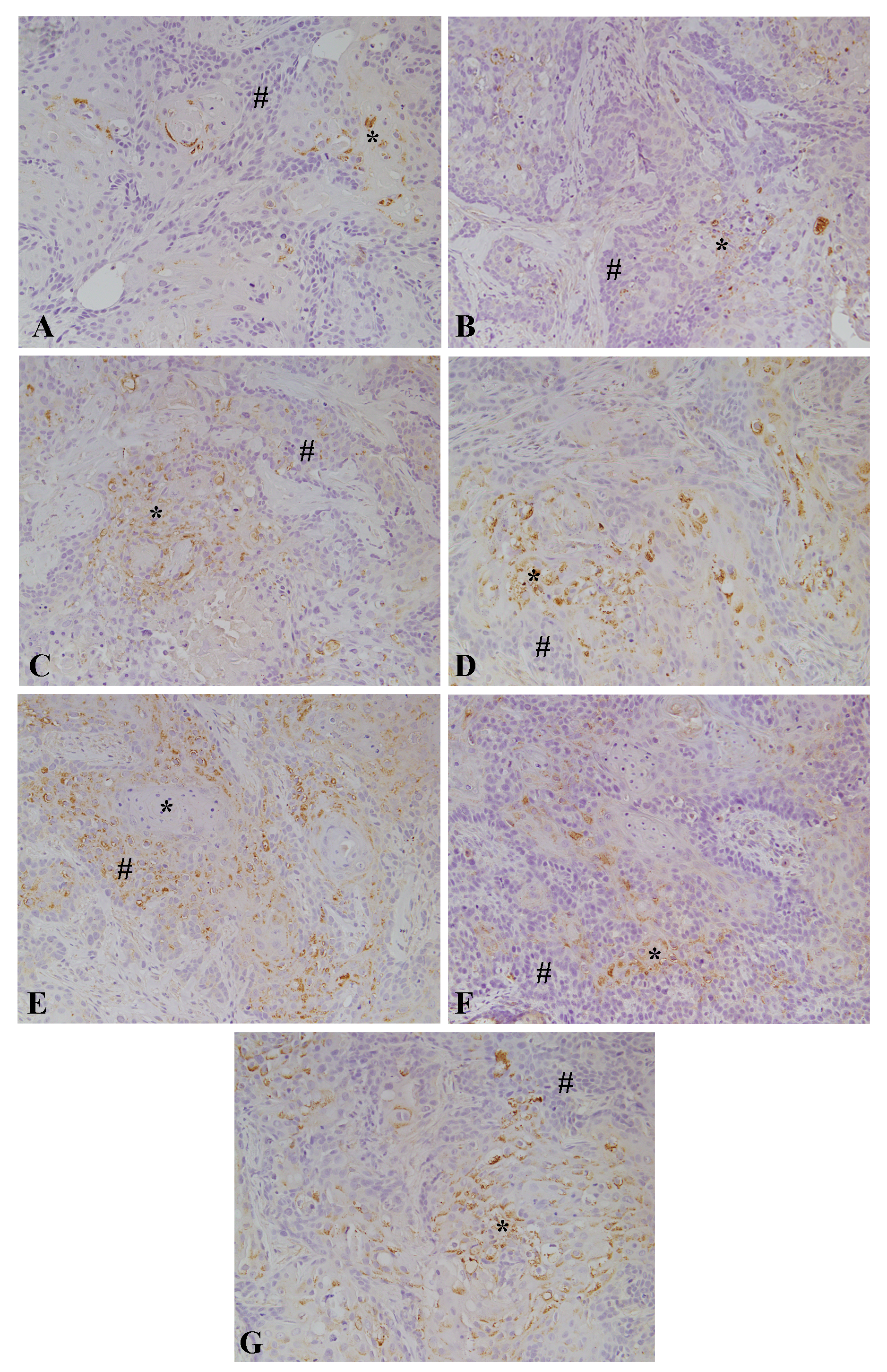

Supplement: S24 Fig — (A-G). Staining for Cd#1, Cd#2, Cd#3, Cd#4, Cd#5, Cd#6 and Cd#7 respectively. The staining for CD24 in Cd#1, Cd#2, Cd#3 and Cd#6 is focal and granular in some of the well-differentiated cells (*)in the center of the tumor nests, whereas most of the tumor cells are negative for CD24. For Cd#4,Cd#5 and Cd#7, the staining for CD24 is granular with moderate to strong staining in the well differentiated cells (*) located in the center of tumor nests, whereas the staining is absent in the less differentiated cells (#) located at the periphery of the tumor nests. All images are at a magnification of 200X. (TIF) [file pone.0207877.s024.tif]

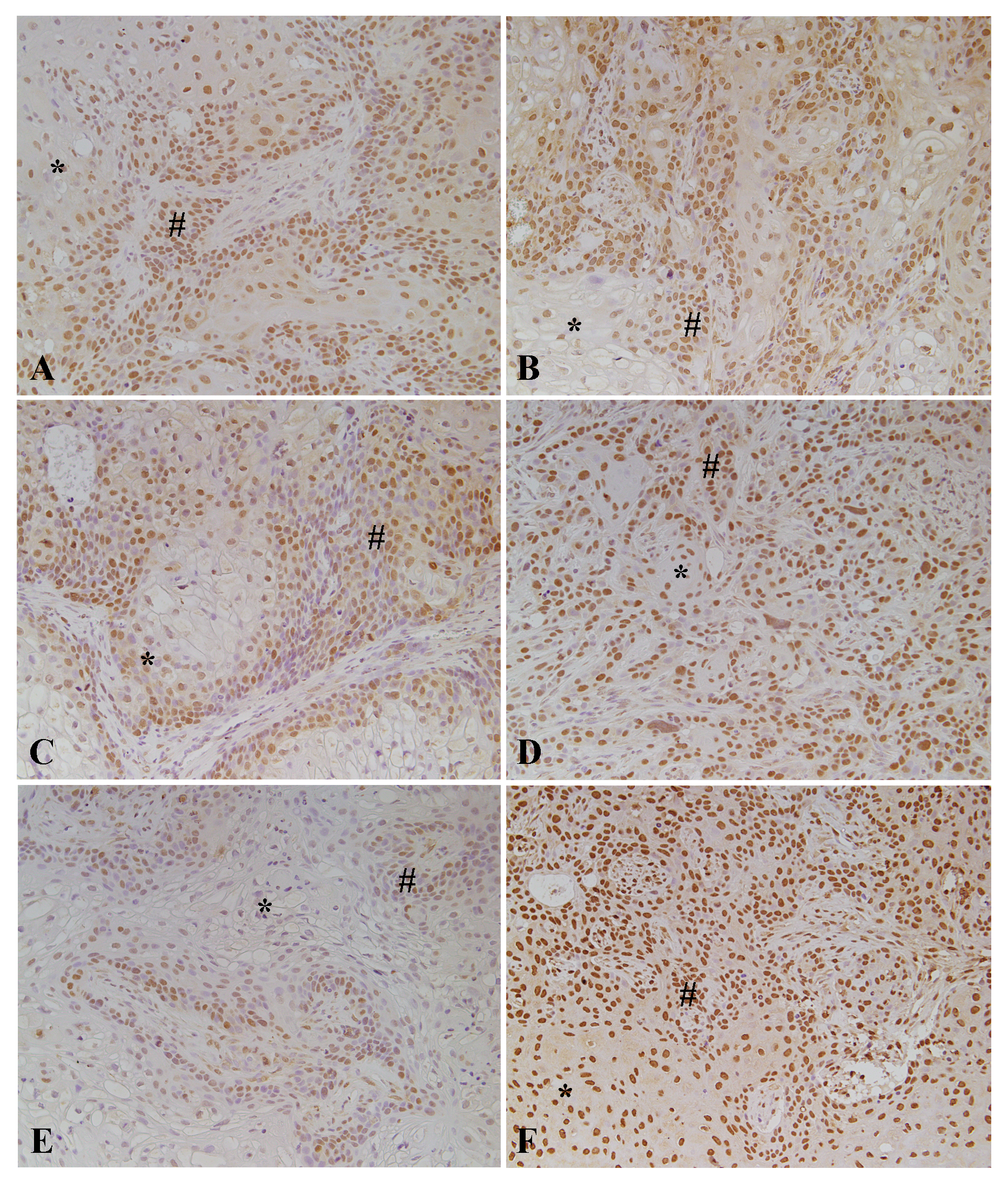

Supplement: S25 Fig — (A-F). Staining for As#1, As#2, As#3, As#4, As#5 and As#6 respectively. There is moderate to strong nuclear staining for p63 in the less differentiated peripheral tumor cells (#), whereas the well-differentiated cells (*) in the center of the tumor nests showed weak or no staining for p63. For As#4 and As#6, there is strong nuclear staining in both the differentiated as well as the less differentiated peripheral tumor cells. All images are at a magnification of 200X. (TIF) [file pone.0207877.s025.tif]

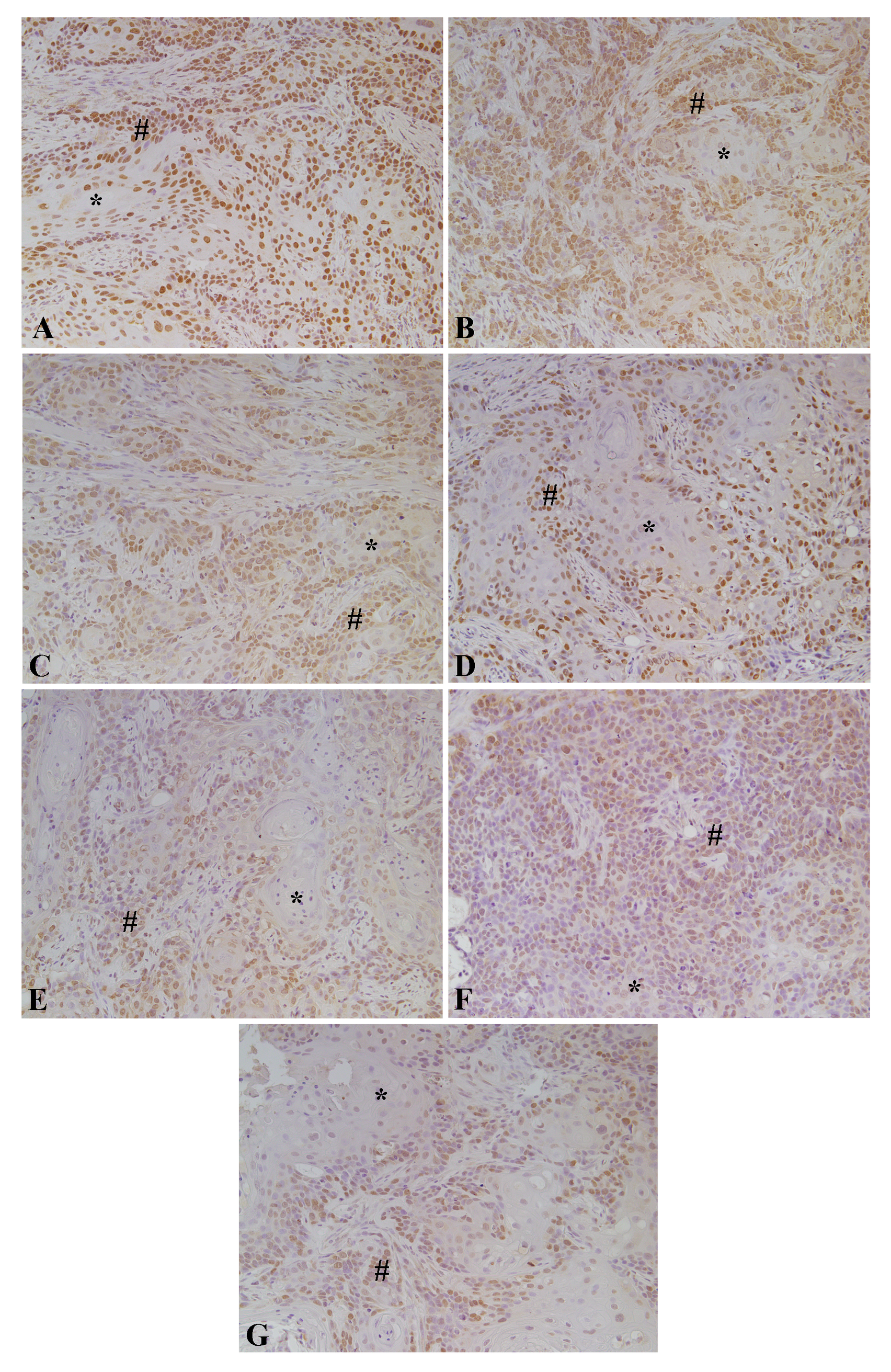

Supplement: S26 Fig — (A-G). Staining for Cd#1, Cd#2, Cd#3, Cd#4, Cd#5, Cd#6 and Cd#7 respectively. In Cd#1, Cd#2, Cd#4 and Cd#6, there is moderate to strong nuclear staining of p63 in the less differentiated peripheral tumor cells, whereas the well-differentiated cells in the center of the tumor nests showed weak or no staining for p63. For Cd#5 and Cd#7, the staining is weak to moderate in the less differentiated peripheral tumor cells. All images are at a magnification of 200X. (TIF) [file pone.0207877.s026.tif]

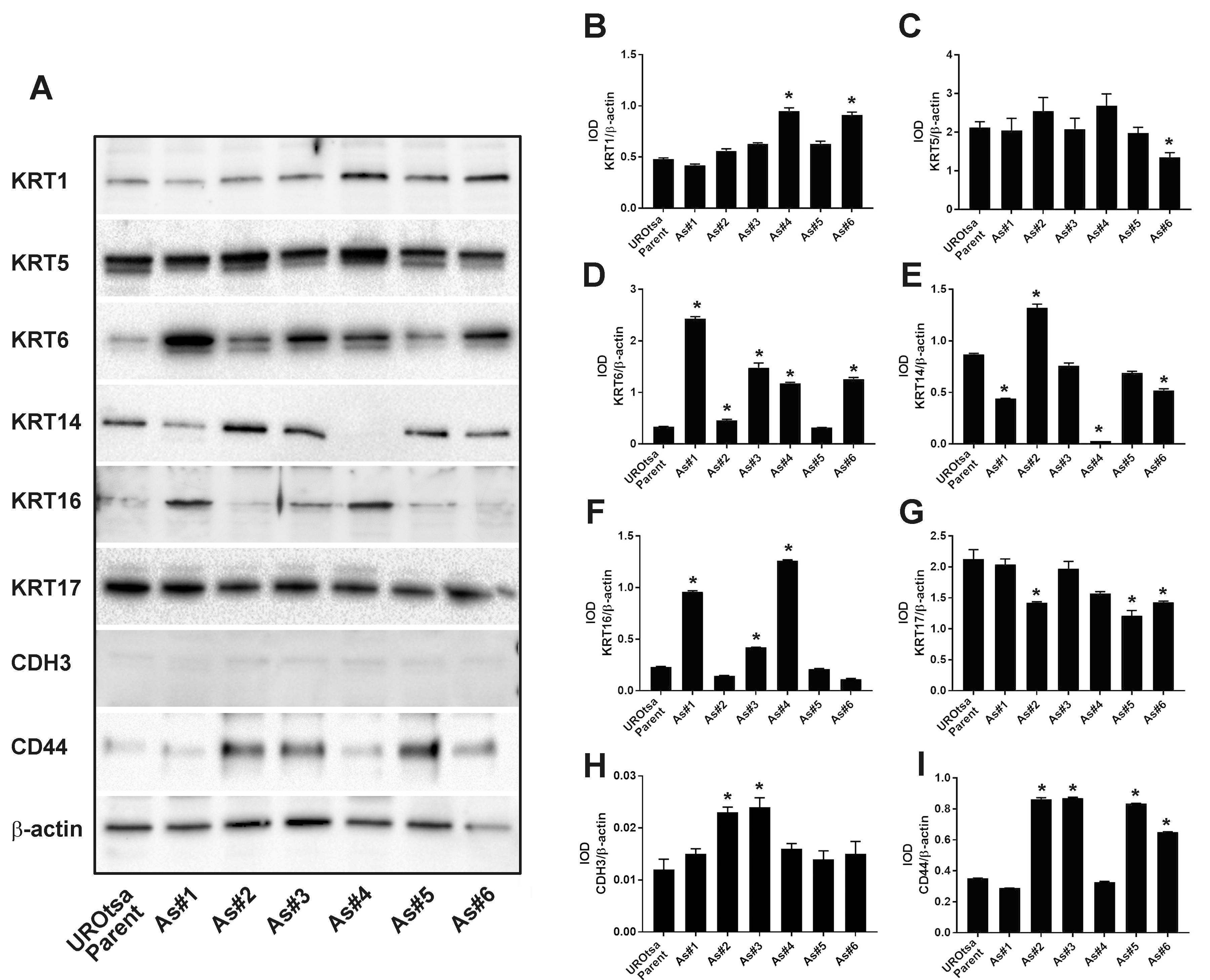

Supplement: S27 Fig — (A-I). Western blots for KRT1, KRT5, KRT6, KRT14, KRT16, KRT17, CDH3, CD44 and β-actin. Integrated optical density (IOD) of each band was normalized to that of β-actin. * indicates significantly different at p < 0.05 from parent UROtsa cells. (TIF) [file pone.0207877.s027.tif]

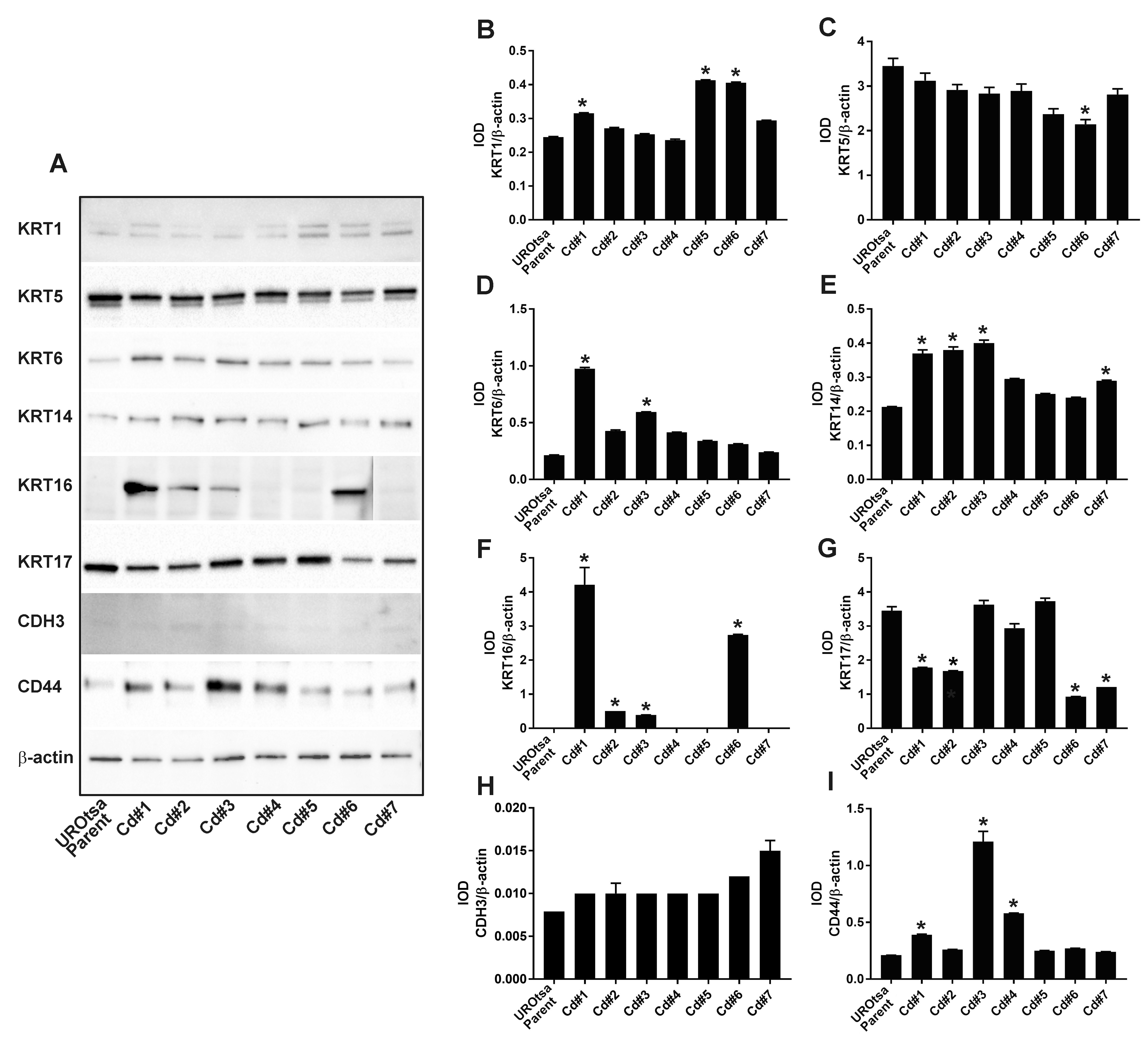

Supplement: S28 Fig — (A-I). Western blots for KRT1, KRT5, KRT6, KRT14, KRT16, KRT17, CDH3, CD44 and β-actin. Integrated optical density (IOD) of each band was normalized to that of β-actin. * indicates significantly different at p < 0.05 from parent UROtsa cells. (TIF) [file pone.0207877.s028.tif]

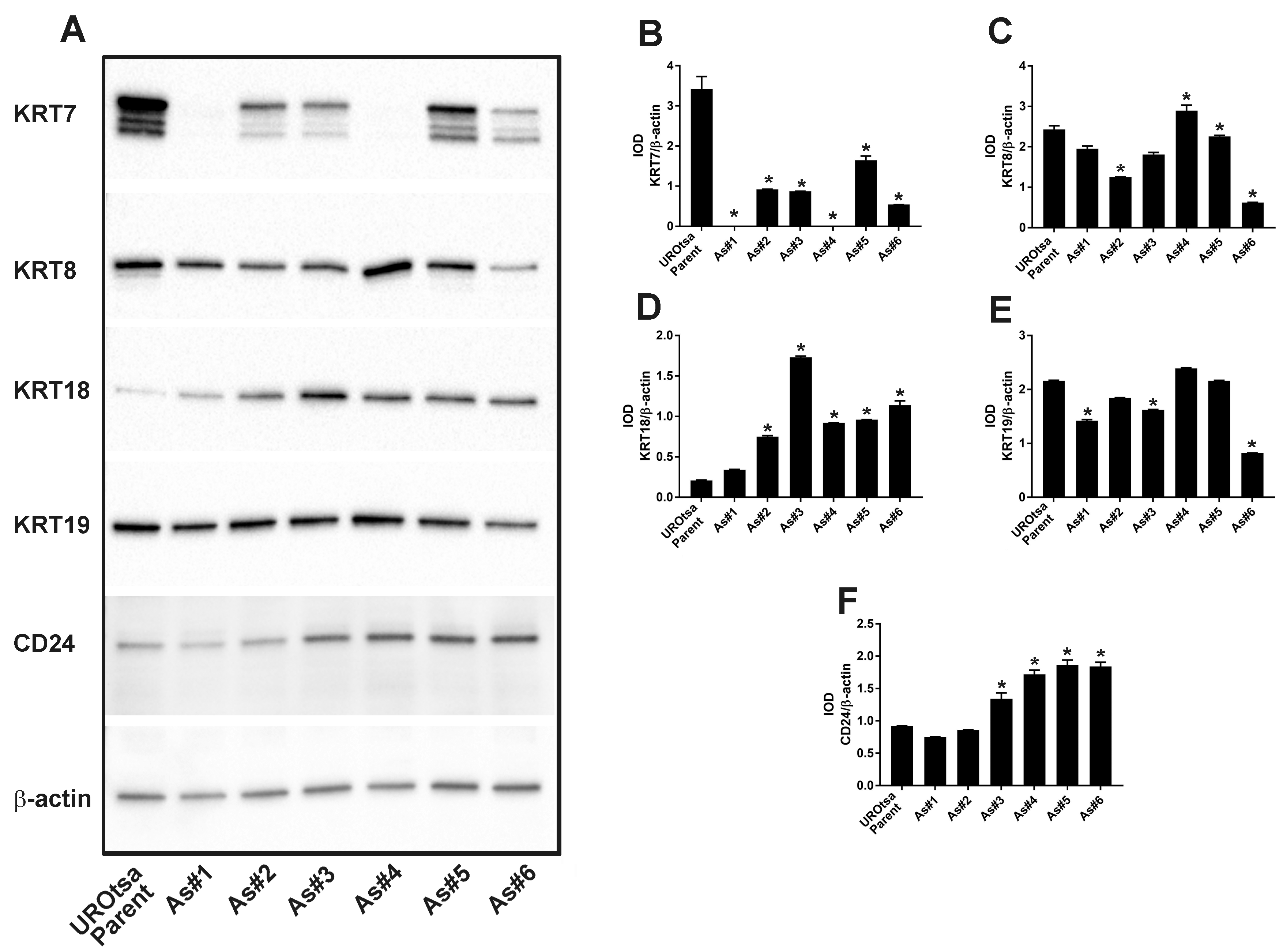

Supplement: S29 Fig — (A-F). Western blots for KRT7, KRT8, KRT18, KRT19, CD24 and β-actin. Integrated optical density (IOD) of each band was normalized to that of β-actin. * indicates significantly different at p < 0.05 from parent UROtsa cells. (TIF) [file pone.0207877.s029.tif]

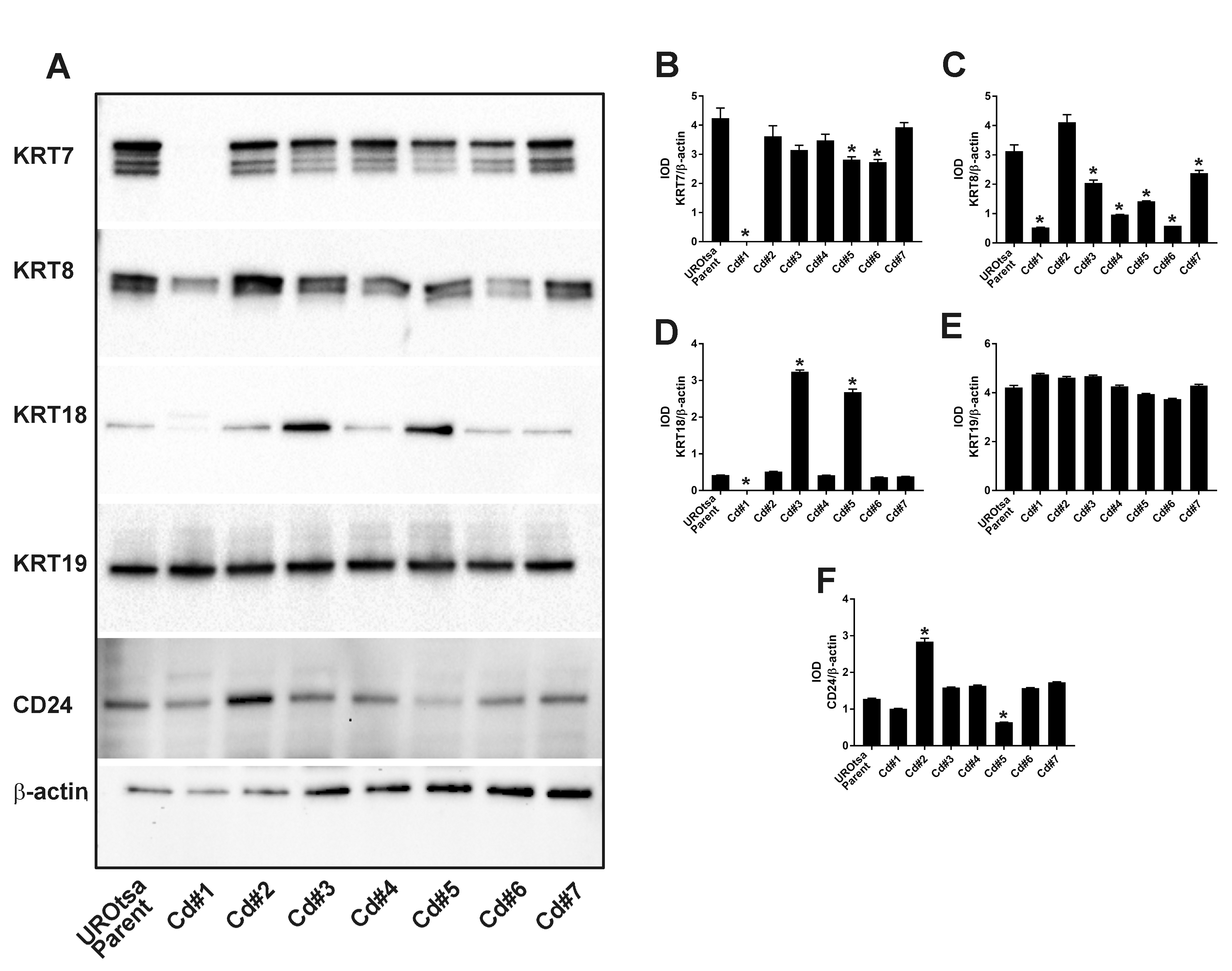

Supplement: S30 Fig — (A-F). Western blots for KRT7, KRT8, KRT18, KRT19, CD24 and β-actin. Integrated optical density (IOD) of each band was normalized to that of β-actin. * indicates significantly different at p < 0.05 from parent UROtsa cells. (TIF) [file pone.0207877.s030.tif]
