# Supplementary material for: The urothelial cell line UROtsa transformed by arsenite and cadmium display basal characteristics associated with muscle invasive urothelial cancers
Source: PLoS One. 2018 Dec 14;13(12):e0207877. doi: 10.1371/journal.pone.0207877 (PMC6294394; doi:10.1371/journal.pone.0207877)
Supplement: S1 Table — (DOCX) [file pone.0207877.s031.docx]

S1 Table. Antibodies used in Immunohistochemical studies

| Antigen | Source | Cat. No | Dilution/Concentration |
| --- | --- | --- | --- |
| Keratin 1 (KRT1) | Invitrogen | PA5-26699 | 1:100 |
| Keratin 5 (KRT5) | Invitrogen | PA5-29670 | 1:400 |
| Keratin 6 (KRT6) | Santa Cruz Biotechnology | sc-514520 | 1:500 |
| Keratin 7 (KRT7) | Dako (Agilent) | M7018 | 1:50 |
| Keratin 14 (KRT14) | Invitrogen | PA5-16722 | 1:400 |
| Keratin 16 (KRT16) | Abcam | ab8741 | 1:40 |
| Keratin 17 (KRT17) | Dako (Agilent) | M7046 | 1:20 |
| Keratin 19 (KRT19) | Abcam | ab15463 | 1:100 |
| Keratin 20 (KRT20) | Invitrogen | PA5-22125 | 1:100 |
| CD44 | R&D Systems | MAB7045 | 10 µg/ml |
| P-cadherin (CDH3) | Santa Cruz Biotechnology | sc-7893 | 1:200 |
| TP63 (p63) | Abnova | MAB10290 | 1:100 |
| CD24 | Invitrogen | MA5-11833 | 1:10 |
