# Supplementary material for: The urothelial cell line UROtsa transformed by arsenite and cadmium display basal characteristics associated with muscle invasive urothelial cancers
Source: PLoS One. 2018 Dec 14;13(12):e0207877. doi: 10.1371/journal.pone.0207877 (PMC6294394; doi:10.1371/journal.pone.0207877)
Supplement: S2 Table — (DOCX) [file pone.0207877.s032.docx]

S2 Table

List of genes identified with basal and luminal type of muscle invasive bladder cancer (MIBC)

| Genes | Molecular Subtype | Catalog No./unique Assay ID | Source |
| --- | --- | --- | --- |
| CDH3 | Basal | qHsaCID0015341 | BIO-RAD |
| CD44 | Basal | qHsaCID0013679 | BIO-RAD |
| KRT1 | Basal | qHsaCID0011275 | BIO-RAD |
| KRT5 | Basal | qHsaCID0047798 | BIO-RAD |
| KRT6A | Basal | qHsaCID0036985 | BIO-RAD |
| KRT6B | Basal | qHsaCID0003069 | BIO-RAD |
| KRT6C | Basal | qHsaCID0046713 | BIO-RAD |
| KRT14 | Basal | qHsaCID0047868 | BIO-RAD |
| KRT16 | Basal | qHsaCID0047866 | BIO-RAD |
| CD24 | Luminal | QT00216811 | QIAGEN |
| CYP2J2 | Luminal | qHsaCID0016825 | BIO-RAD |
| ERBB2 | Luminal | qHsaCID0012766 | BIO-RAD |
| ERBB3 | Luminal | qHsaCID0018397 | BIO-RAD |
| FABP4 | Luminal | qHsaCID0036778 | BIO-RAD |
| FGFR3 | Luminal | qHsaCID0042267 | BIO-RAD |
| FOXA1 | Luminal | qHsaCID0002547 | BIO-RAD |
| GATA3 | Luminal | qHsaCID0017793 | BIO-RAD |
| GPX2 | Luminal | qHsaCID0014084 | BIO-RAD |
| KRT7 | Luminal | qHsaCID0038533 | BIO-RAD |
| KRT8 | Luminal | qHsaCID0038745 | BIO-RAD |
| KRT18 | Luminal | qHsaCID0035037 | BIO-RAD |
| KRT19 | Luminal | qHsaCID0046788 | BIO-RAD |
| KRT20 | Luminal | qHsaCID0007981 | BIO-RAD |
| PPARG | Luminal | qHsaCID0044425 | BIO-RAD |
| XBP1 | Luminal | qHsaCID0010491 | BIO-RAD |
| KRT17 | - | qHsaCID0023841 | BIO-RAD |
| TP63 | - | qHsaCID0036332 | BIO-RAD |
