# Supplementary material for: The urothelial cell line UROtsa transformed by arsenite and cadmium display basal characteristics associated with muscle invasive urothelial cancers
Source: PLoS One. 2018 Dec 14;13(12):e0207877. doi: 10.1371/journal.pone.0207877 (PMC6294394; doi:10.1371/journal.pone.0207877)
Supplement: S3 Table — (DOCX) [file pone.0207877.s033.docx]

S3 Table. Antibodies used in Western Analysis

| Antigen | Source | Cat. No | Dilution/Concentration |
| --- | --- | --- | --- |
| Keratin 1 (KRT1) | Invitrogen | PA5-26699 | 1:1000 |
| Keratin 5 (KRT5) | Invitrogen | PA5-29670 | 1:20,000 |
| Keratin 6 (KRT6) | Santa Cruz Biotechnology | sc-514520 | 1:5000 |
| Keratin 7 (KRT7) | ThermoFisher Scientific | MA5-1198 | 1µg/ml |
| Keratin 14 (KRT14) | Abcam | Ab181595 | 1:20,000 |
| Keratin 16 (KRT16) | Abcam | ab8741 | 1:100 |
| Keratin 17 (KRT17) | Abcam | Ab51056 | 1:10,000 |
| Keratin 19 (KRT19) | Abcam | Ab52625 | 1:20,000 |
| CD44 | R&D Systems | MAB7045 | 1:2500 |
| P-cadherin (CDH3) | Santa Cruz Biotechnology | sc-7893 | 1:50 |
| CD24 | Santa Cruz Biotechnology | Sc-70598 | 1:200 |
